# A molecular recognition platform for the simultaneous sensing of diverse chemical weapons†

**DOI:** 10.1039/d2sc00299j

**Published:** 2022-03-23

**Authors:** Lintao Zeng, Tianhong Chen, Beitong Zhu, Seyoung Koo, Yonghe Tang, Weiying Lin, Tony D. James, Jong Seung Kim

**Affiliations:** School of Light Industry and Food Engineering, Guangxi University Nanning 530004 China; College of Chemistry, Beijing Normal University Beijing 100875 China; Department of Chemistry, Korea University Seoul 02841 Korea jongskim@korea.ac.kr; Guangxi Key Laboratory of Electrochemical Energy Materials, Institute of Optical Materials and Chemical Biology, School of Chemistry and Chemical Engineering, Guangxi University Nanning 530004 China weiyinglin2013@163.com; Department of Chemistry, University of Bath Bath BA2 7AY UK t.d.james@bath.ac.uk; School of Physics, Henan Normal University Xinxiang 453007 China

## Abstract

Chemical warfare agents (CWAs) such as phosgene and nerve agents pose serious threats to our lives and public security, but no tools can simultaneously screen multiple CWAs in seconds. Here, we rationally designed a robust sensing platform based on 8-cyclohexanyldiamino-BODIPY (BODIPY-DCH) to monitor diverse CWAs in different emission channels. *Trans*-cyclohexanyldiamine as the reactive site provides optimal geometry and high reactivity, allowing *trans*-BODIPY-DCH to detect CWAs with a quick response and high sensitivity, while *cis*-BODIPY-DCH has much weaker reactivity to CWAs due to intramolecular H-bonding. Upon reaction with phosgene, *trans*-BODIPY-DCH was rapidly converted to imidazolone BODIPY (<3 s), triggering green fluorescence with good sensitivity (LOD = 0.52 nM). *trans*-BODIPY-DCH coupled with nerve agent mimics, affording a blue fluorescent 8-amino-BODIPY tautomer. Furthermore, a portable test kit using *trans*-BODIPY-DCH displayed an instant response and low detection limits for multiple CWAs. This platform enables rapid and highly sensitive visual screening of various CWAs.

## Abstract 2

Chemical warfare agents (CWAs) such as phosgene and nerve agents pose serious threats to our lives and public security, necessitating tools that can simultaneously screen multiple CWAs in seconds.

## Graphical abstract

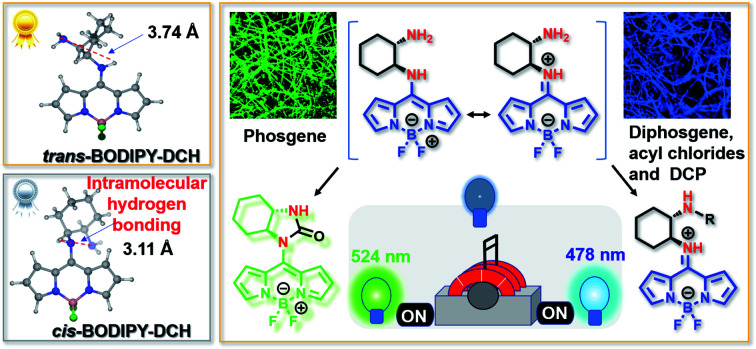

## Introduction

Public security is a global concern closely related to our health and safety. Today, public security around the world faces potential risks from COVID-19, AIDS, antimicrobial resistance, and chemical warfare agents (CWAs). CWAs are a class of highly toxic chemicals used as lethal weapons on battlefields such as World War I and World War II, slaughtering millions of soldiers and civilians.^1–3^ Moreover, some CWAs have been used by terrorists to create panic attacks over the past few decades, posing a serious threat to public security and human health.

Phosgene, a colorless and toxic gaseous substance, has been used as a CWA in several wars.^1^ Phosgene can cause serious lung and respiratory damage within 2 min, and exposure to 90 ppm for more than 30 min is fatal.^2–4^ Organophosphorus nerve agents have been identified as the most dangerous CWAs, which can readily bind with acetylcholinesterase to inhibit conduction of nerve impulses, leading to organ failure and death within seconds.^2,3^ Acyl chlorides can also cause serious burns to the skin and mucous membranes, leading to dermal and respiratory diseases.^5,6^ Nevertheless, phosgene, organophosphorus, and acyl chlorides are indispensable raw materials for industrial production of pesticides,^7^ medicines,^8^ and dyes and polymers.^9^ As such, they pose a serious threat to public security when used by terrorists as CWAs or released in accidental industrial leaks. It is therefore highly desirable to devise a simple and efficient tool to rapidly measure phosgene, nerve agents and acyl chlorides for both public security and manufacturing safety.

Conventional approaches for the detection of phosgene, nerve agents and volatile acyl chlorides rely on stationary equipment such as gas chromatographs^10^ and liquid chromatographs.^11^ These instruments are less portable and measurements take a long time, making them unsuitable for rapid security screening in subways, airports, train stations and at customs. Since fluorescent sensors are powerful optical devices capable of generating fluorescent signal changes in host–guest recognition patterns, they have been used to visually determine analytes with high selectivity and sensitivity, low cost, and easy operation.^12–16^ Recently, numerous fluorescent sensors have been developed that separately detect phosgene or nerve agents using various reaction sites.^17–36^ However, their detection limits are unsatisfactory, or the response rate is slow because the reactivity of recognition units such as pyridine, aromatic amine, and hydroxyl groups is low. Moreover, these sensors ignore various toxic acyl chlorides, such as diphosgene and acetyl chloride, which still pose a threat to public security.

Given the wide availability, broad application, and high toxicity of phosgene and acyl chlorides, the development of sensors to thoroughly screen them is urgently needed. Unfortunately, research on fluorescent sensors for acyl chlorides remains at the embryonic stage. To date, only two fluorescent sensors have been reported for oxalyl chloride,^37,38^ but these fluorescent sensors can neither detect nerve agents nor other acyl chlorides. Although great efforts have been made to prepare various sensors to individually detect each analyte, the complex procedures result in low detection efficiency and do not meet the need of rapid security screening in subways, airports, train stations, and customs.

To this end, it is highly desirable to design a versatile sensing platform for the detection of phosgene, nerve agents and various acyl chlorides using different emission channels. In this context, we propose a robust detection platform based on 8-amino-BODIPY and cyclohexanyldiamine recognition groups to simultaneously sense multiple CWAs with a fast response and high sensitivity. In addition, a portable and easy-to-use test kit was made with a melt-blown nonwoven fabric and *trans*-BODIPY-DCH for the rapid and accurate detection of phosgene, diphosgene, nerve agent mimics and other volatile acyl chlorides.

## Results and discussion

**Fig. 1:**
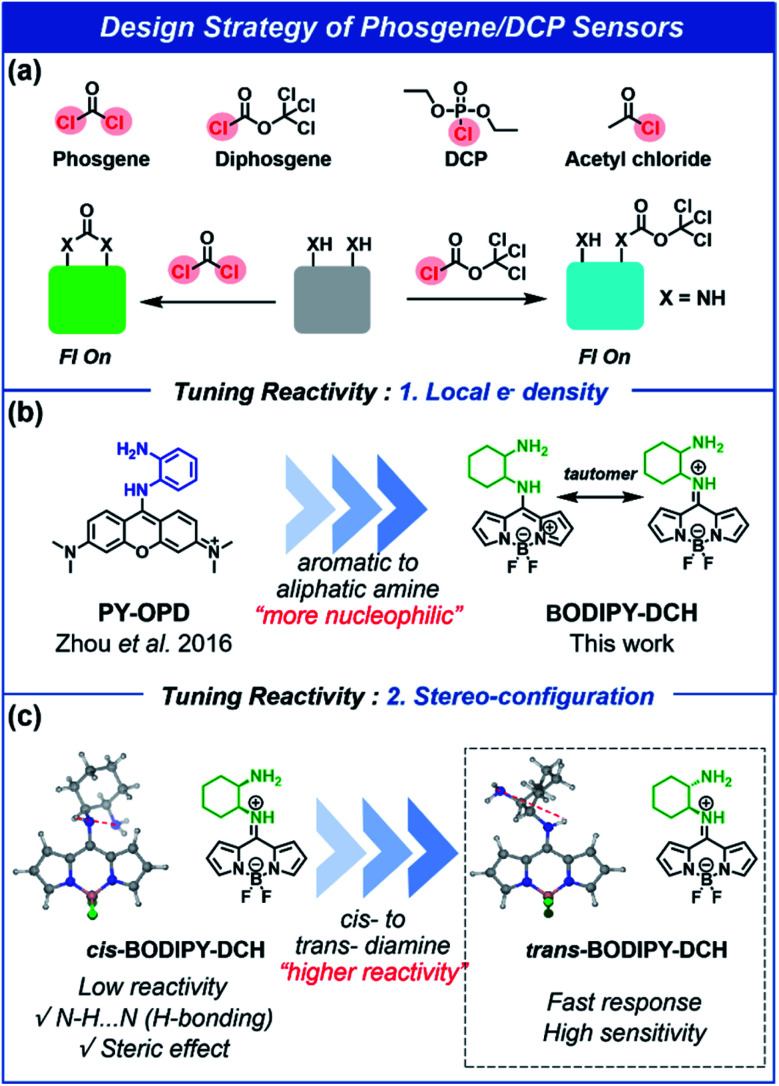
Schematic illustration of the design strategy for the sensing of phosgene, diphosgene, diethyl chlorophosphate (DCP), and acetyl chloride.

Most common acyl chloride and organophosphorus nerve agents have one reactive group in their molecular structure, whereas phosgene has two reactive units. Although *o*-phenylenediamine has been used as a recognition site to differentiate phosgene from diethyl chlorophosphate (DCP),^33^ aromatic amines have poor nucleophilic substitution reactivity towards DCP and acyl chlorides. Therefore, in this study, 1,2-diaminocyclohexane (DCH) has been used in place of an aromatic amine to improve the sensitivity and response rate. Since 1,2-diaminocyclohexane has two primary aliphatic NH_2_ with high p*K*_a_ values (p*K*_a1_ = 9.60 and p*K*_a2_ = 6.21),^39^ it exhibits high nucleophilic reactivity towards acyl chlorides, phosgene, and nerve agent mimics (Fig. S1 and S2†). 1,2-Diaminocyclohexane exists as two isomers, *trans*- and *cis*-structures, which were anchored at the 8-position of BODIPY to form 8-amino-BODIPY derivatives *trans*- or *cis*-BODIPY-DCH with one aliphatic NH_2_ and one aromatic NH_2_ for differential binding with acyl chlorides and phosgene (Fig. 1). The 8-amino-BODIPY derivatives (*trans*- or *cis*-BODIPY-DCH) exist as hemicyanine tautomers with pale blue fluorescence.^40,41^ In the presence of phosgene, the two adjacent amines in DCH combine with phosgene to form imidazolone. As a result, the BODIPY core no longer exists as a tautomer, and photo-induced electron transfer (PET) is prohibited, resulting in a strong green fluorescence along with a noticeable color change. In the presence of acyl chlorides or DCP, the primary aliphatic NH_2_ is transformed into amide that inhibits the PET process and leads to a large fluorescence enhancement in the blue channel. Therefore, these sensors (*trans*-, *cis*-BODIPY-DCH) enable the simultaneous detection of phosgene, DCP and some other acyl chlorides using different emission channels.

*trans*-BODIPY-DCH and *cis*-BODIPY-DCH were easily prepared by the nucleophilic substitution of 8-Cl-BODIPY with *trans*-cyclohexanediamine and *cis*-cyclohexanediamine at room temperature, respectively, as outlined in Fig. S3.† Their chemical structures and intermediates were characterized by ^1^H NMR, ^13^C NMR, HRMS, and X-ray diffraction of single crystals, respectively. The detailed experimental procedure and characterization data are provided in the ESI.†

**Fig. 2:**
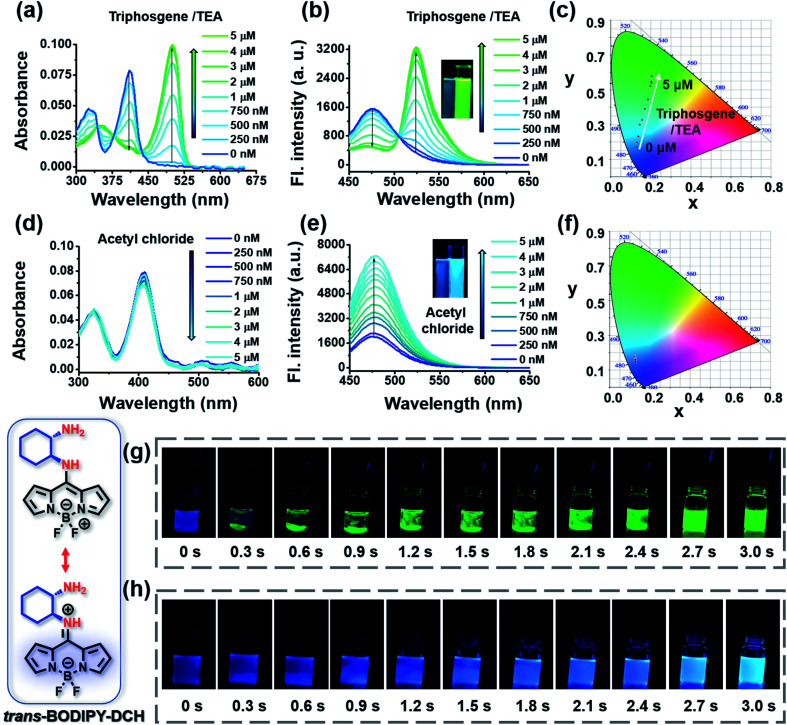
UV-vis absorption and fluorescence spectra changes of *trans*-BODIPY-DCH (5.0 μM) after the addition of (a and b) incremental triphosgene (0–5.0 μM)/TEA (100.0 μM) and (d and e) acetyl chloride (0–5.0 μM). (c and f) CIE1931 coordinates of *trans*-BODIPY-DCH (5.0 μM) from (b) and (e), respectively. Time-lapse images for the fluorescence response of *trans*-BODIPY-DCH (5.0 μM) towards (g) triphosgene (10.0 μM)/TEA or (h) acetyl chloride (10.0 μM). *λ*_ex_ = 430 nm, slits: 2.5 nm/5.0 nm.

The capability of the sensing platform to detect phosgene and acyl chlorides was investigated in chloroform, and a hypotoxic counterpart triphosgene was used to generate phosgene *in situ* in the presence of triethylamine (TEA). As shown in Fig. 2a, *trans*-BODIPY-DCH exhibited a strong absorption at 410 nm. Then, upon the addition of (0–5.0 μM)/TEA (100.0 μM), the absorption peak gradually decreased, while a bathochromic peak at 500 nm emerged and progressively increased with an isosbestic point at 435 nm, suggesting that *trans*-BODIPY-DCH exhibits high reactivity and sensitivity towards phosgene. *trans*-BODIPY-DCH exhibits a weak fluorescence band at 478 nm in CHCl_3_ (*Φ*_f_ = 0.12) due to photo-electron transfer (PET) from the 1,2-diaminocyclohexane group to the BODIPY core (Fig. 2b). Upon addition of triphosgene/TEA to *trans*-BODIPY-DCH, a new fluorescence band at 524 nm (*Φ*_f_ = 0.46) was significantly enhanced, while a remarkable fluorescence variation from faint blue to bright green was observed. The Commission International de L'Eclairage (CIE) chromaticity coordinates (Fig. 2c and Table S1†) also exhibited notable changes from blue to green with a coordinate variation from (0.1247, 0.1805) to (0.2046, 0.5793). Accordingly, a linear correlation (*R*^2^ = 0.994) was obtained between the emission intensity (*F*_524_) of *trans*-BODIPY-DCH and the concentration of triphosgene in the range of 0–3.0 μM (Fig. S4†) with a detection limit of 0.52 nM based on 3*σ*/*k*. These results demonstrated that *trans*-BODIPY-DCH could be used as a ratiometric fluorescence sensor for phosgene with ultra-sensitivity.

Next, we explored the spectral response of *trans*-BODIPY-DCH to acetyl chloride. Upon the addition of acetyl chloride, *trans*-BODIPY-DCH displayed a negligible change in the UV-vis absorption (Fig. 2d), but its initial fluorescence band at 478 nm was significantly enhanced (*Φ*_f_ = 0.62). These observations might be attributed to the stronger nucleophilicity of the free aliphatic NH_2_ (p*K*_a_ = 9.61) in DCH over the aromatic amine. An excellent linear relationship (*R*^2^ = 0.994) was established between the emission intensity at 478 nm and the concentration of acetyl chloride over a range from 0–5.0 μM (Fig. S4†) resulting in an LOD of 0.77 nM by the principle of 3*σ*/*k*. These results confirmed that *trans*-BODIPY-DCH could serve as a sensitive fluorescence sensor to detect acetyl chloride.

We also examined the spectral response of *trans*-BODIPY-DCH towards DCP and a family of other acyl chlorides including diphosgene, triphosgene, oxalyl chloride and benzoyl chloride. As shown in Fig. S5 and S6,† addition of DCP (5.0 μM) to *trans*-BODIPY-DCH (5.0 μM) induced a negligible change in UV-vis absorption spectra of the sensor, but triggered obvious fluorescence enhancement at 478 nm (*Φ*_f_ = 0.31). Likewise, *trans*-BODIPY-DCH (5.0 μM) displayed a large fluorescence enhancement at 478 nm after the addition of various acyl chlorides (Table S2†). Therefore, the *trans*-BODIPY-DCH could serve as a versatile sensor for the high throughput detection of phosgene, DCP and some other acyl chlorides.

A rapid response is critical for the on-site detection of toxic phosgene and acyl chlorides. To examine the sensing rate, the time-dependent fluorescence response of *trans*-BODIPY-DCH (5.0 μM) was recorded after the addition of analytes (10.0 μM). For phosgene (Fig. S7†), the fluorescence intensity ratio (*F*_524_/*F*_478_) of *trans*-BODIPY-DCH (5.0 μM) reached a plateau within 3 s. Likewise, the fluorescence intensity at 478 nm reached maxima within a few seconds (∼3 s) after addition of each analyte including DCP, acetyl chloride, diphosgene, oxalyl chloride, thionyl chloride, benzoyl chloride and triphosgene (Fig. S7 and Table S2†). In addition, a live video and time-lapse fluorescence images of *trans*-BODIPY-DCH (5.0 μM) upon the addition of analytes were recorded (Fig. 2g and Movie 1†). When the triphosgene was added, obvious green fluorescence emerged in the local area within 0.3 s. As triphosgene diffused and decomposed into phosgene, the green fluorescence became much brighter and diffused over the whole solution within 3 s, suggesting that *trans*-BODIPY-DCH is a rapidly responding sensor for phosgene. Upon the addition of acetyl chloride, the blue fluorescence of *trans*-BODIPY-DCH (5.0 μM) lit up immediately and reached a maximum within 3 s as well (Fig. 2h and Movie 2†). Those observations confirmed that *trans*-BODIPY-DCH could achieve a rapid response toward both phosgene and volatile acyl chlorides.

**Fig. 3:**
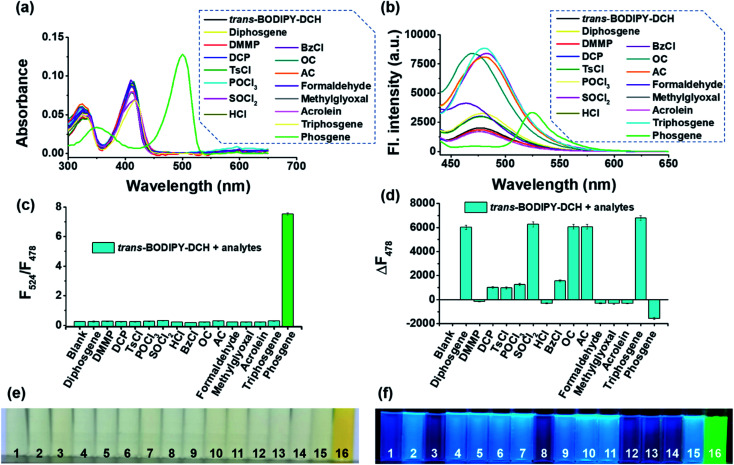
(a) Absorption and (b) fluorescence spectra of *trans*-BODIPY-DCH (5.0 μM) after the addition of different analytes in chloroform. (c) Ratio fluorescence intensity changes and (d) fluorescence intensity changes at 478 nm of *trans*-BODIPY-DCH solution (5.0 μM) after the addition of analytes (10.0 μM). (e and f) The color and fluorescence image of *trans*-BODIPY-DCH solution (5.0 μM) after the addition of analytes (10.0 μM): (1) blank, (2) diphosgene, (3) DMMP, (4) DCP, (5) TsCl, (6) POCl_3_ (7) SOCl_2_, (8) HCl, (9) BzCl, (10) OC, (11) AC, (12) FA, (13) MGO, (14) acrolein, (15) triphosgene, and (16) phosgene. *λ*_ex_ = 430 nm, slits: 2.5 nm/5.0 nm, error bars are ±SD, *n* = 3.

Next, we evaluated the fluorescence response of *trans*-BODIPY-DCH (5.0 μM) towards other species (10.0 μM) including diphosgene, dimethyl methylphosphonate (DMMP), DCP, tosyl chloride (TsCl), POCl_3_, SOCl_2_, HCl, benzoyl chloride (BzCl), oxalyl chloride (OC), acetyl chloride (AC), formaldehyde (FA), methylglyoxal (MGO), acrolein, triphosgene and phosgene. As shown in Fig. 3a–f, phosgene brought about an obvious color and ratiometric fluorescence change of *trans*-BODIPY-DCH solution. By contrast, a significant fluorescence enhancement was observed for diphosgene, DCP and other acyl chlorides. The potential interfering species like HCl, DMMP, formaldehyde, methylglyoxal and acrolein induced negligible color or fluorescence changes, confirming that this sensor could avoid interference from HCl, NO, and aldehydes, and achieve accurate and specific detection of acyl chlorides and phosgene. Therefore, *trans*-BODIPY-DCH is superior to most of the reported sensors for phosgene or DCP.^24–30^ To further check the selectivity of *trans*-BODIPY-DCH without any false positive fluorescence signal for acid and aldehydes, competitive selectivity experiments were performed. After addition of a mixed solution of triphosgene and HCl to the *trans*-BODIPY-DCH solution containing TEA (100 μM), a newly formed emission band at 524 nm was observed, which was assigned to the fluorescence band of *trans*-BODIPY-ICO. The same observation was found when the *trans*-BODIPY-DCH solution was treated with triphosgene and formaldehyde. When the mixture solution of acetyl chloride and HCl was added to the *trans*-BODIPY-DCH solution containing TEA (100 μM), the sensor only showed an enhanced fluorescence band at 478 nm (Fig. S8†). Therefore, we can conclude that *trans*-BODIPY-DCH is a robust sensor for the discriminative detection of acyl chlorides and phosgene.

To validate our design strategy, we prepared a mirror compound, *cis*-BODIPY-DCH from (1*R*,2*R*)-1,2-diaminocyclohexane and 8-Cl-BODIPY. As seen in Fig. S9,†*cis*-BODIPY-DCH displayed a strong absorption peak at 408 nm, which gradually converted to a bathochromic absorption band at 498 nm when incremental triphosgene (0–40.0 μM) was added. The initial fluorescence band at 474 nm gradually decreased, meanwhile a new fluorescence band at 522 nm progressively increased until the concentration of triphosgene was 8 equiv. with respect to the sensor, affording an obvious fluorescence change from faint blue to green (Fig. S9†). *cis*-BODIPY-DCH could also sense acetyl chloride, diphosgene, triphosgene, OC, or SOCl_2_. Upon the addition of 40 μM acetyl chloride, diphosgene, triphosgene, OC, or SOCl_2_, *cis*-BODIPY-DCH exhibited a large fluorescence enhancement at 474 nm (Fig. S10–S14†), and the LOD for acetyl chloride was determined to be 6.92 nM (Fig. S15†). A live video and time-lapse fluorescence images were also recorded. The triphosgene gave a bright green color to the *cis*-BODIPY-DCH solution within 4 s (Fig. S9 and Movie 3†), and the fluorescence intensity reached a maximum within 4 s after addition of triphosgene (Fig. S16†). The solution could also be lit up by acetyl chloride within 4 s (Movie 4†). These results demonstrated that *cis*-BODIPY-DCH could also achieve a rapid response towards phosgene and acyl chlorides with good selectivity. Nevertheless, *cis*-BODIPY-DCH was not as good as the *trans*-one in terms of sensitivity and response rate. The LODs of *cis*-BODIPY-DCH for phosgene and acetyl chloride were 23.49 nM and 6.92 nM, respectively, which are much higher than those of *trans*-BODIPY-DCH (LOD = 0.52 nM and 0.77 nM for phosgene and acetyl chloride, respectively).

**Fig. 4:**
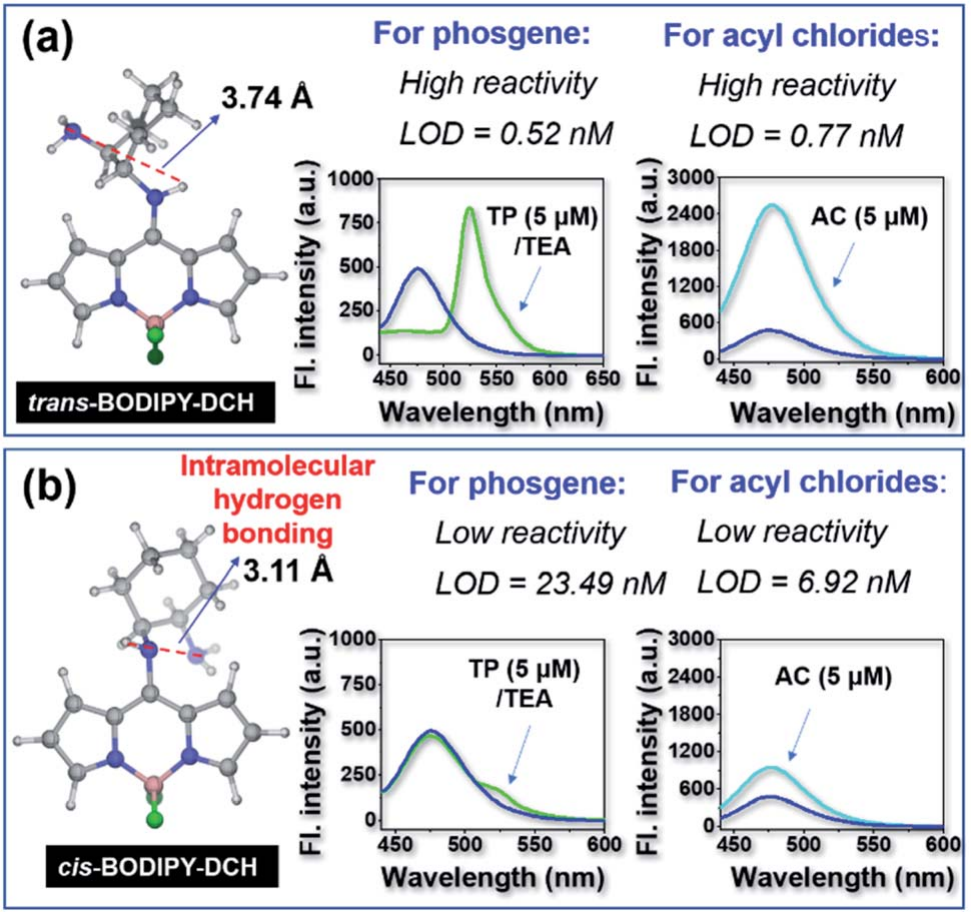
The optimized geometry and sensing performances of (a) *trans*-BODIPY-DCH and (b) *cis*-BODIPY-DCH towards phosgene and acyl chlorides.

To get insight into this observation, we performed density functional theory (DFT) calculations at the B3LYP/6-31G* level,^42^ and gained optimized geometries of *cis* and *trans*-BODIPY-DCH (Fig. 4). In *cis*-BODIPY-DCH, two amine groups were located at the same side with a dihedral angle of 68.13°, and the space distance between the hydrogen atom (in N1) and N4 was 3.11 Å. Thus, the two neighboring amines in *cis*-BODIPY-DCH are prone to form intramolecular H-bonding (Fig. 4b), which weakened the reactivity of the amines. In contrast, two amine groups were located at different sites with a dihedral angle of 168.22° in *trans*-BODIPY-DCH, and the distance between the hydrogen atom (in N1) and N4 is 3.74 Å (Fig. 4a, S18 and S19†), hindering the formation of intramolecular H-bonds. Furthermore, we measured the ^1^H NMR spectrum of *cis*- and *trans*-BODIPY-DCH in CDCl_3_ and DMSO-*d*_6_, respectively. In DMSO-*d*_6_, the proton signal of the aromatic NH in *cis*-BODIPY-DCH was located at 5.66 ppm (Fig. S20†), which shifted to 8.48 ppm in CDCl_3_ (Fig. S21†), confirming the stable intramolecular H-bonding (IMHB) of N–H⋯N.^43^ In this case, IMHB in *cis*-BODIPY-DCH would form a stable five member ring to weaken the reactivity of two amino groups for phosgene and acyl chlorides. In contrast, IMHB does not form for *trans*-BODIPY-DCH according to the ^1^HNMR spectra in CDCl_3_ where the proton signal of the aromatic NH has completely disappeared (Fig. S21†), confirming that the aliphatic NH_2_ in *trans*-BODIPY-DCH could quickly react with both phosgene and acyl chlorides.

**Fig. 5:**
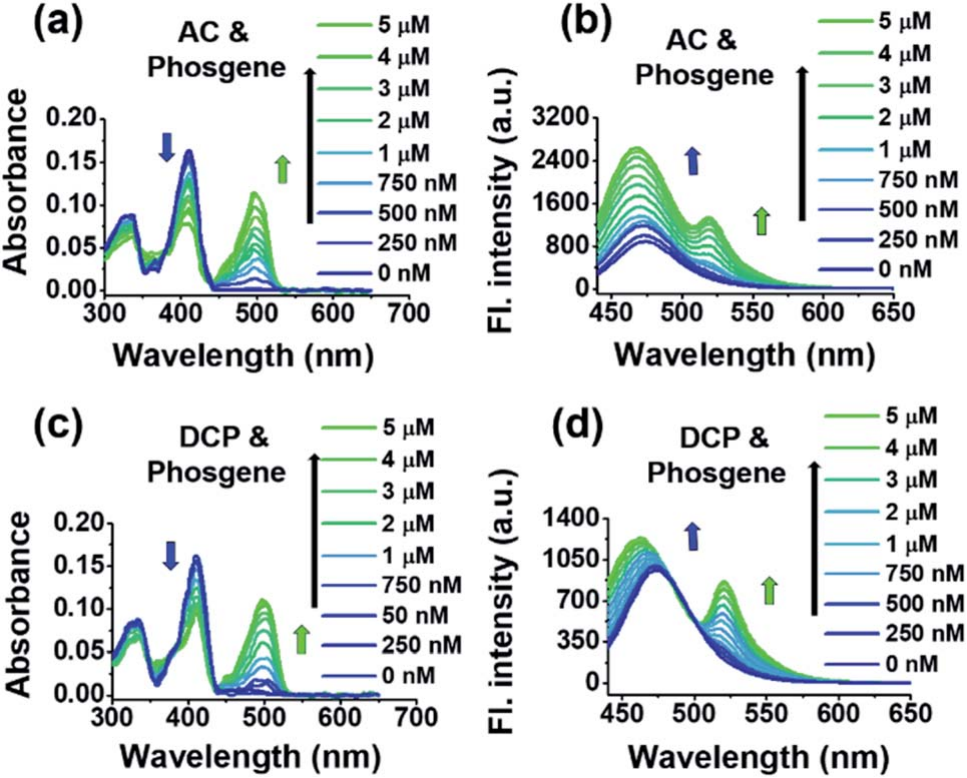
UV-vis absorption spectra and fluorescence spectra changes of the *trans*-BODIPY-DCH solution (5.0 μM) containing TEA (100.0 μM) after the addition of incremental analytes (0–5.0 μM), including: (a and b) AC and phosgene, (c and d) DCP and phosgene. *λ*_ex_ = 430 nm, slits: 2.5 nm/2.5 nm.

We then used *trans*-BODIPY-DCH to simultaneously detect phosgene and DCP, as well as some volatile acyl chlorides. When a mixture of triphosgene and acetic chloride in a 1/3 molar fraction was added to the *trans*-BODIPY-DCH solution, a bathochromic peak appeared at 500 nm, while the initial absorption peak at 410 nm decreased to half. By carefully examining the fluorescence spectra changes (Fig. 5b), we found that *trans*-BODIPY-DCH displayed two emission bands at 524 and 478 nm, respectively. Similar results were also observed when the mixture of triphosgene and some other acyl chlorides including DCP, OC, BzCl, and SOCl_2_ was added to *trans*-BODIPY-DCH (Fig. 5c, d and S22†), demonstrating that *trans*-BODIPY-DCH has a good capability for the simultaneous detection of phosgene, DCP and acyl chlorides.

**Fig. 6:**
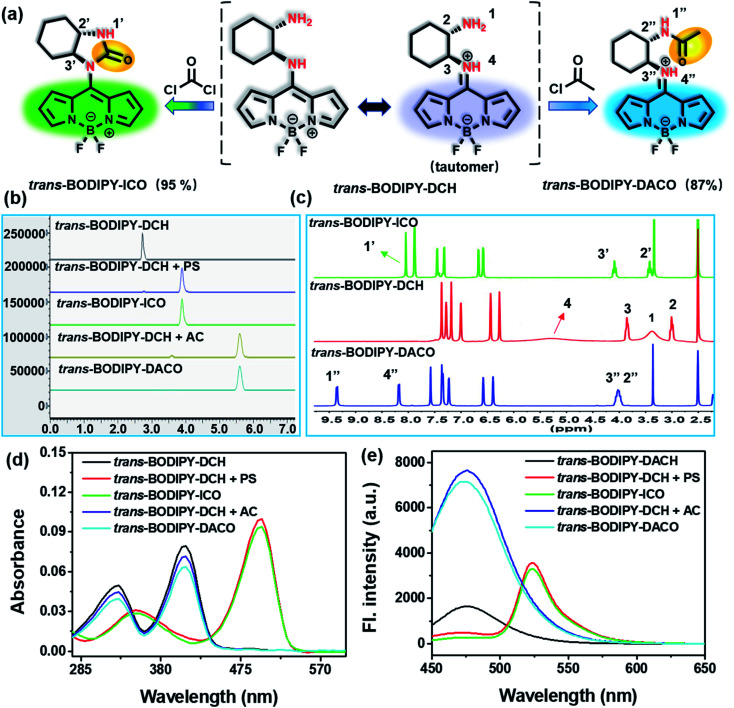
(a) Proposed working mechanism of *trans*-BODIPY-DCH. (b) HPLC profiles of *trans*-BODIPY-DCH, the reaction mixture of *trans*-BODIPY-DCH and triphosgene/TEA, *trans*-BODIPY-ICO, and the reaction mixture of *trans*-BODIPY-DCH and acetyl chloride, *trans*-BODIPY-DACO. (c) ^1^H NMR spectrum of *trans*-BODIPY-DCH, *trans*-BODIPY-ICO and *trans*-BODIPY-DACO in DMSO-*d*_6_. (d) UV-vis absorption spectra and (e) fluorescence spectra of *trans*-BODIPY-DCH, the reaction mixture solution of *trans*-BODIPY-DCH and triphosgene/TEA, *trans*-BODIPY-ICO, and the reaction mixture solution of *trans*-BODIPY-DCH and acetyl chloride, *trans*-BODIPY-DACO. *λ*_ex_ = 430 nm, slits: 2.5 nm/5 nm.

To investigate the interaction mode, we employed high performance liquid chromatography (HPLC) to analyze the reaction mixture of *trans*-BODIPY-DCH with phosgene and acetyl chloride, respectively. As shown in Fig. 6a and b, a predominant elution peak appeared at 3.9 min in the HPLC profile, attributed to the reaction of *trans*-BODIPY-DCH with phosgene in a specific manner. We then isolated the major product *trans*-BODIPY-ICO from reaction mixtures and analyzed the NMR and HR-MS spectra. As revealed in Fig. 6c, the chemical shifts of NH_2_ (H1) and NH (H4) in *trans*-BODIPY-DCH were located at 3.38 and 5.25 ppm, respectively. After reaction with phosgene, the proton signal of H1 shifted from 3.38 to 8.05 ppm, and the proton signal of H4 disappeared. In addition, the proton signals of two adjacent CH groups at 3.00 and 3.85 ppm slightly shifted downfield. Moreover, a significant carbonyl signal at 159.4 ppm appeared in the ^13^C NMR spectra (Fig. S23†), which was assigned to the carbonyl group of *trans*-BODIPY-ICO. HR-MS also confirmed the structure of *trans*-BODIPY-ICO, where a predominant peak at *m*/*z* = 331.1538 was attributed to [C_16_H_17_BF_2_N_4_O]^+^ (calc. 331.1536) (Fig. S24†). UV-vis absorption and fluorescence spectra (Fig. 6d and e) further verified that the major product was *trans*-BODIPY-ICO. These results clearly demonstrated that two adjacent amino groups in *trans*-BODIPY-DCH have coupled with phosgene to form imidazolone, which resulted in a red-shift of the UV-vis absorption and fluorescence spectra.

For acetyl chloride, a predominant elution peak emerged at 5.6 min, exhibiting 87% of the total peak area in the HPLC profile (Fig. 6b). The major product *trans*-BODIPY-DACO was isolated from the reaction mixture, and characterized by NMR and HR-MS spectra. The initial proton signals of NH_2_ (H1) and NH (H4) located at 3.38 and 5.25 ppm, respectively, shifted to 9.36 and 8.19 ppm after reaction with acetyl chloride (Fig. 6c). Additionally, the proton signal of the adjacent CH (H2) significantly shifted downfield (Fig. 6c and S25†). Moreover, an important carbon signal at 171.9 ppm was observed in the ^13^C NMR spectrum (Fig. S26†), corresponding to the C=O group of *trans*-BODIPY-DACO. The HR-MS spectra also confirmed the structure of *trans*-BODIPY-DACO, where a peak at *m*/*z* = 347.1853 was ascribed to [C_17_H_21_BF_2_N_4_O]^+^ (calc. 347.1849) (Fig. S27†). These results confirmed that acetyl chloride had coupled with the aliphatic amino group to produce an obvious fluorescence enhancement in the blue channel (Fig. 6e).

**Fig. 7:**
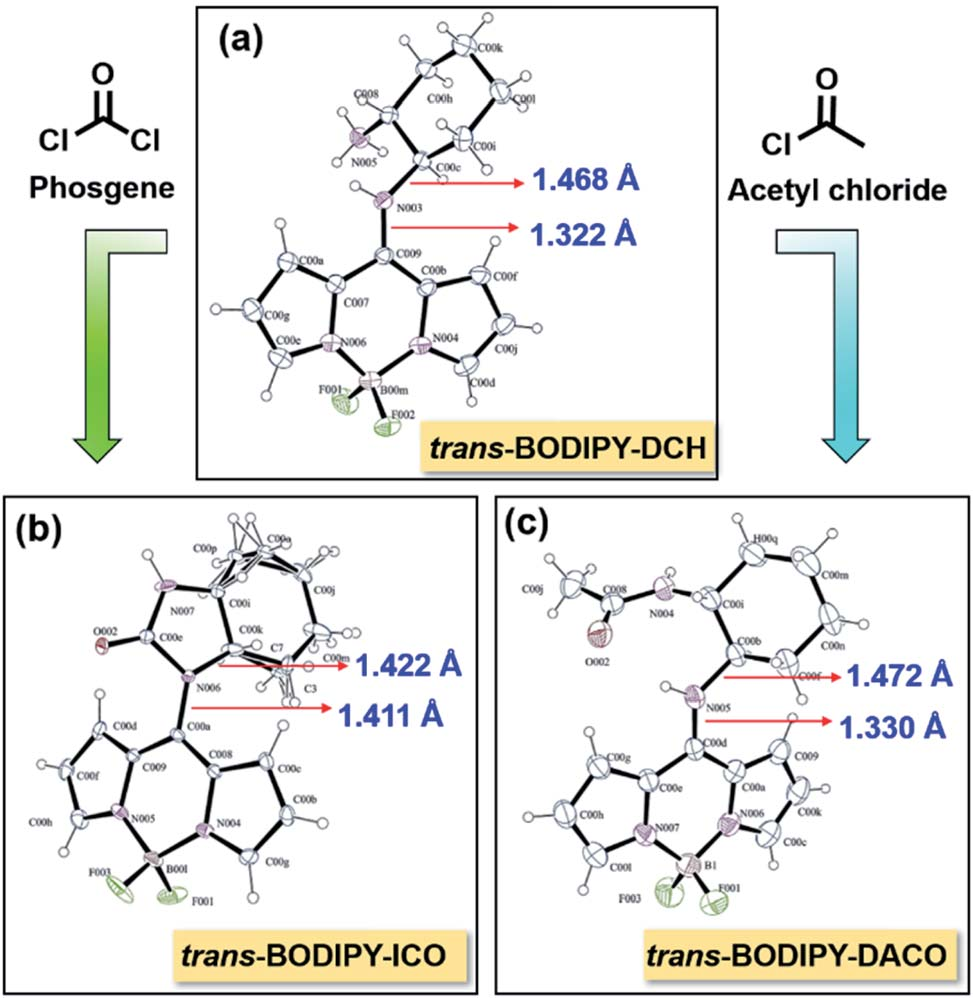
X-ray single crystallography by ORTEP drawing of (a) *trans*-BODIPY-DCH, (b) *trans*-BODIPY-ICO and (c) *trans*-BODIPY-DACO with 30% probability thermal ellipsoids.

We successfully obtained single crystals of *trans*-BODIPY-DCH, *trans*-BODIPY-ICO and *trans*-BODIPY-DACO from dichloromethane and *n*-hexane, respectively. In *trans*-BODIPY-DCH, the DCH is perpendicular to the BODIPY plane with a dihedral angle of 88.2° (Fig. 7a, Tables S3 and S4†), and the bond length between N003 and C009 is 1.322 Å, much shorter than the ordinary bond length of the N–C single bond (N003–C00C, 1.468 Å, Table S5†). This confirms that the *trans*-BODIPY-DCH exists as a tautomer with C=N as the dominant resonance structure (Fig. 7a). The *trans*-BODIPY-DCH tautomer was a cross-conjugated system, in which the push–pull interaction of the pyrrole nitrogen atoms was interrupted, which would expand the HOMO–LUMO band gap and lead to a significant blue-shift in the UV-vis absorption and fluorescence spectra. Hence, the *trans*-BODIPY-DCH tautomer exhibited an absorption band at 410 nm and a weak fluorescence band at 478 nm. After reaction with acetic chloride, the aliphatic NH_2_ with higher p*K*_a_ in DCH was transformed to an amide in *trans*-BODIPY-DACO, but the aromatic NH was unaltered, which is evident from the single crystal structure in Fig. 7c. The bond length between N005 and C00D was 1.330 Å (Table S6†), shorter than the ordinary bond length of the N–C single bond (N005–C00b, 1.472 Å), suggesting that the *trans*-BODIPY-DACO existed as a resonance tautomer (Fig. S28†). By contrast, the two adjacent amines reacted with phosgene to form imidazolone, as can be seen in the single crystal structure in Fig. 7b. In *trans*-BODIPY-ICO, a bond like that of a normal N–C single bond (N006–C00K, 1.422 Å) (Fig. 7b and Table S7†), implying that its tautomer was transformed to normal BODIPY after reaction with phosgene (Fig. S29†).

Density function theory (DFT) calculations revealed that the HOMOs of *trans*- and *cis*-BODIPY-DCH are enriched in the pyrrole ring, while the LUMO is mainly located at the BODIPY core and part of DCH (Fig. S30 and S31†). Thus, the PET process from NH_2_ quenched the intrinsic blue fluorescence of BODIPY-DCH to some extent. After reacting with phosgene, the tautomer was transformed to the normal BODIPY structure, resulting in a large red shift in the absorption and fluorescence spectra. In contrast, the LUMO energy of the tautomer increased along with a slight reduction of the band gap after the *trans*-BODIPY-DCH reacted with acetyl chloride. In this case, the PET process of *trans*-BODIPY-DACO in the excited state was prohibited, and thus a significant fluorescence enhancement was observed in the blue channel.

**Fig. 8:**
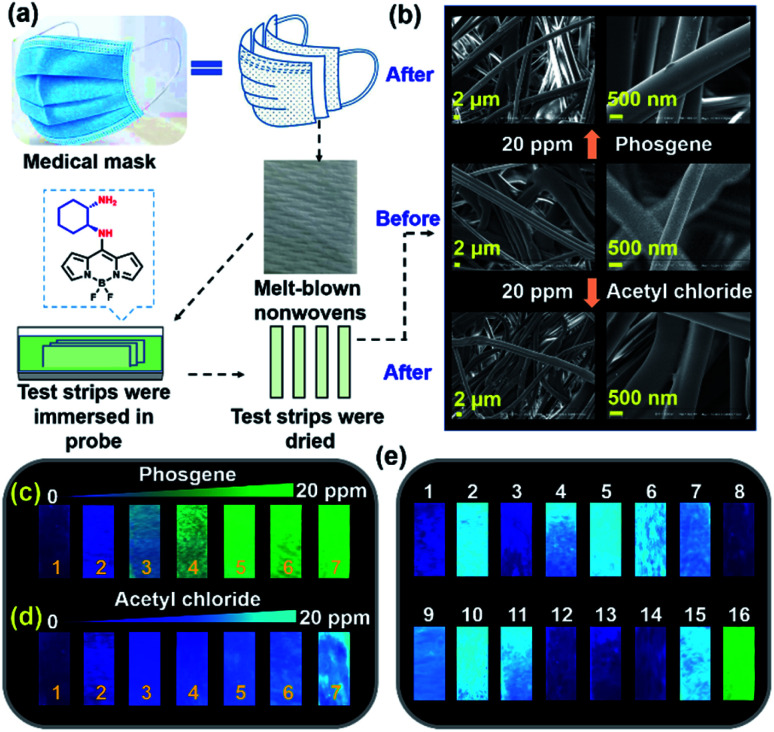
(a) Schematic diagram of the fabrication of *trans*-BODIPY-DCH test strips. (b) SEM micrographs of *trans*-BODIPY-DCH test strips before and after exposure to phosgene and acetyl chloride vapors. Fluorescence images of *trans*-BODIPY-DCH test strips after exposure to (c) phosgene and (d) acetyl chloride vapor at the concentrations of 0–20 ppm: (1) 0, (2) 0.5, (3) 1, (4) 2.5, (5) 5, (6) 10, and (7) 20 ppm. (e) Fluorescence images of *trans*-BODIPY-DCH test strips after exposure to 20 ppm of phosgene and various nerve agent mimics and acyl chlorides. All fluorescence images were acquired under a 365 nm lamp: (1) blank, (2) diphosgene, (3) DMMP, (4) DCP, (5) TsCl, (6) POCl_3_ (7) SOCl_2_, (8) HCl, (9) BzCl, (10) OC, (11) AC, (12) FA, (13) MGO, (14) acrolein, (15) triphosgene, and (16) phosgene.

We evaluated the cytotoxicity of *trans*-BODIPY-DCH by using a CCK-8 assay. L929 cells and HeLa cells exhibited 90% and 92% cell survival rates after incubation with 20 μM *trans*-BODIPY-DCH for 24 h (Fig. S32†), respectively, demonstrating that *trans*-BODIPY-DCH exhibits good biocompatibility. We then fabricated test strips for visualizing phosgene, DCP and gaseous acyl chlorides using *trans*-BODIPY-DCH. Herein, we used melt-blown nonwovens as a matrix to fabricate test strips because nanofibers exhibit high surface to volume ratios suitable for the adsorption of the sensor and analytes, which is especially beneficial for the detection of gaseous compounds. After exposure to 20 ppm of phosgene or acetyl chloride, the nanofibers still kept their original smooth morphology without exhibiting any damage (Fig. 8b).

We next employed them for the visual detection of phosgene, DCP and acyl chlorides in the gas phase. As shown in Fig. 8c, *trans*-BODIPY-DCH-loaded nonwoven test strips emitted faint blue fluorescence under 365 nm light. Upon exposure to various concentrations of phosgene (0.5–20 ppm) under ambient conditions for 1 min, a significant fluorescence transition from faint blue to strong green fluorescence was observed. By comparison, the test strips exhibited a noticeable blue fluorescence enhancement with acetyl chloride (Fig. 8d). Likewise, DCP, diphosgene, and some other acyl chlorides (*i.e.* triphosgene, OC and BzCl) also stimulated the test strips to produce strong blue fluorescence (Fig. S33†). The *trans*-BODIPY-DCH-loaded nonwoven test strips exhibited good capability for the high throughput determination of phosgene, DCP and acyl chlorides, and this sensing platform based on melt-blown nonwovens is superior to some conventional systems.^44,45^

To further assess the selectivity, *trans*-BODIPY-DCH-loaded nonwoven test strips were exposed to various analytes (20 ppm), including phosgene, diphosgene, DCP, DMMP, TsCl, POCl_3_, SOCl_2_, HCl, BzCl, OC, AC, formaldehyde, methylglyoxal, acrolein, and triphosgene. As shown in Fig. 8e, the phosgene resulted in a significant green fluorescence being observed for the test strip. On the other hand, DCP, diphosgene and other acyl chlorides caused a remarkable blue fluorescence enhancement, while DMMP, HCl, formaldehyde, methylglyoxal and acrolein led to negligible fluorescence changes.

**Fig. 9:**
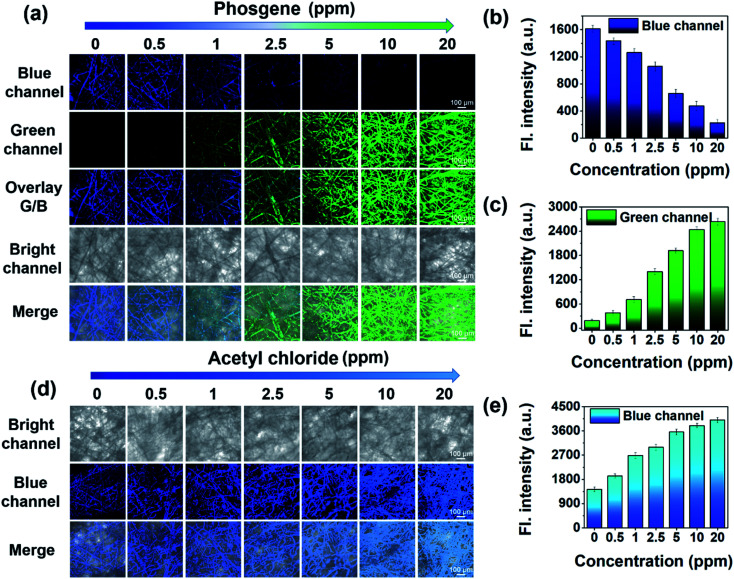
Fluorescence images of *trans*-BODIPY-DCH-loaded test strips after exposure to (a) phosgene and (d) acetyl chloride vapor. Fluorescence intensity in (b) blue and (c) green channel correlated with the level of phosgene. (e) Fluorescence intensity in the blue channel correlates with the level of acetyl chloride. For the blue channel, *λ*_ex_ = 402 nm, *λ*_em_ = 425−475 nm; for the green channel, *λ*_ex_ = 488 nm, *λ*_em_ = 500−530 nm. Error bars represent ±SD, *n* = 5, scale bar: 100 μm.

To visualize analytes on the test strips, we utilized laser confocal fluorescence microscopy to record the fluorescence images of *trans*-BODIPY-DCH-loaded nonwoven test strips before and after addition of analytes. *trans*-BODIPY-DCH-loaded nonwoven test strips themselves exhibited filamentous fibers with faint fluorescence in the blue channel (Fig. 9). After exposure to phosgene (0.5–20 ppm), an obvious fluorescence enhancement was observed from the green channel, and the initial blue fluorescence intensity diminished. Such obvious fluorescence color changes enable us to use our naked eyes to detect toxic phosgene. While DCP, diphosgene, acetyl chloride, triphosgene, OC, BzCl or SOCl_2_ resulted in a remarkable fluorescence enhancement of the test strips in the blue channel (Fig. 9d, S34 and S39†).

## Conclusions

In summary, we have successfully developed a robust sensing platform for the selective detection of phosgene, DCP, and other acyl chlorides using different emission channels based on *trans*- and *cis*-BODIPY-DCH. From UV/fluorescence titrations, single-crystal structure analysis, and DFT calculations, we found that they exhibited similar sensing mechanisms towards CWAs while *trans*-BODIPY-DCH exhibited better detection performance than the *cis*-version due to the intramolecular H-bonding of the *cis*-version. *trans*-BODIPY-DCH underwent nucleophilic substitution with phosgene, resulting in a noticeable and rapid fluorescence change from weak blue fluorescence to bright green fluorescence (<3 s), with a low detection limit (0.52 nM). In contrast, acyl chlorides and DCP resulted in a rapid highly sensitive fluorescence enhancement with *trans*-BODIPY-DCH with a low detection limit (0.77 nM). Furthermore, it could be conveniently loaded onto melt-blown nonwovens to serve as a device for the high throughout and visible detection of gaseous phosgene, diphosgene, DCP, and other acyl chlorides. Significantly, *trans*-BODIPY-DCH-loaded nonwoven test strips could be used for the highly sensitive and visual detection of toxic acyl chlorides at the sub-ppm levels.

GC-MS is the most used technique that can identify chemical warfare agents at concentrations ranging from low ppb to ppm (w/w). HPLC can also be used to determine the derivatization products of phosgene, nerve agents and aryl chlorides at sub-ppm levels, but requires appropriate pro-derivatization procedures. In addition, GC-MS and HPLC analyses require several minutes to complete the measurement, and the instruments are non-portable. In contrast, our portable *trans*-BODIPY-DCH-loaded nonwoven test strips can rapidly determine sub-ppm levels of chemical warfare agents (within 3 seconds), which is particularly suitable for in-field determination of chemical warfare agents without the need for bulky instrumentation.

In summary, this research not only results in a fluorescent platform for the selective detection of phosgene and volatile acyl chlorides, but also provides a promising configuration-based sensing strategy to develop a “one-for-multiple” chemosensor strategy.

## Data availability

All data supporting this study are provided as ESI,† which includes characterization data, crystal data, simultaneous sensing of diverse analytes with *trans*-BODIPY-DCH, the spectral response of *cis*-BODIPY-DCH towards some analytes, X-ray single crystallography, density functional theory calculations, cytotoxicity, test strips for detection of analyte vapor, confocal fluorescence images of test strips for analyte vapor, ^1^H NMR, ^13^C NMR and HR-MS spectra. CCDC numbers: 2094769; 2094607; 2094606.

## Author contributions

T. D. J. and J. S. K. supervised the project. L. Z. contributed to the project design and along with B. Z. carried out experiments on synthesis and characterization of compounds. T. C. and S. K. cultured the single crystals, analyzed the crystal structure, and investigated the sensing mechanism of the sensor and its application. W. L. made conceptualization. Y. T. conducted theoretical calculations. All authors prepared and edited the manuscript.

## Conflicts of interest

There are no conflicts to declare.

## Supplementary Material

SC-013-D2SC00299J-s001

SC-013-D2SC00299J-s002

SC-013-D2SC00299J-s003

SC-013-D2SC00299J-s004

SC-013-D2SC00299J-s005

SC-013-D2SC00299J-s006
