# Supplementary material for: A molecular recognition platform for the simultaneous sensing of diverse chemical weapons
Source: Chem Sci. 2022 Mar 23;13(16):4523–32. doi: 10.1039/d2sc00299j (PMC9020178; doi:10.1039/d2sc00299j)
Supplement: SC-013-D2SC00299J-s001 [file SC-013-D2SC00299J-s001.pdf]

Electronic Supplementary Material (ESI) for Chemical Science.
This journal is © The Royal Society of Chemistry 2022

Electronic Supplementary Information

Molecular recognition platform for the simultaneous sensing of diverse chemical weapons

Lintao Zeng,[a#] Tianhong Chen,[b#] Beitong Zhu,[a] Seyoung Koo,[c] Yonghe Tang,[d] Weiying Lin,*[d] Tony D James*[e, f] and Jong Seung Kim*[c]

[a] School of Light Industry and Food Engineering, Guangxi University, Nanning, 530004, China.

[b] College of Chemistry, Beijing Normal University, Beijing, 100875, China.

[c] Department of Chemistry, Korea University, Seoul, 02841, Korea. E-mail: jongskim@korea.ac.kr (J.S. Kim)

[d] Guangxi Key Laboratory of Electrochemical Energy Materials, Institute of Optical Materials and Chemical Biology, School of Chemistry and Chemical Engineering, Guangxi University, Nanning, 530004, China. E-mail: weiyinglin2013@163.com

[e] Department of Chemistry, University of Bath, Bath, BA2 7AY, UK. E-mail: t.d.james@bath.ac.uk (T.D. James)

[f] School of Physics, Henan Normal University, Xinxiang 453007, China.

[#] These authors contributed equally to this work.

Contents

1. Experimental section ··· S3

2. The pK_a prediction of ***trans*-BODIPY-DCH** ·· S8

3. Simultaneous sensing of diverse analytes with **trans-BODIPY-DCH** ····················· S10

4. The spectral response of ***cis*-BODIPY-DCH** towards some analytes ······················ S14

5. X-ray single crystallography ·· S18

6. Density functional theory (DFT) calculations ·· S25

7. Cytotoxicity of ***trans*-BODIPY-DCH** ··· S26

8. ***trans*-BODIPY-DCH**-loaded test strips for detection of analytes vapor ··················· S27

9. Confocal fluorescence images of test strips for analytes vapor ······························ S27

10. Structure characterization ··· S30

11. References ··· S41

1. Experimental section

1.1 Materials and instruments

All reagents were purchased from Sigma-Aldrich and used directly without further purification. Melt-blown nonwovens (component: polypropylene) were purchased from Sinopec Shanghai Petrochemical. Co., LTD, China. ^{1}H and ^{13}C NMR spectra were measured on a Bruker AV spectrometer by using tetramethylsilane (TMS) as the internal standard. High-resolution mass spectra (HRMS) were recorded on a HP-1100 LC-MS spectrometer. UV−vis absorption and fluorescence spectra were obtained by a Hitachi UV-3310 spectrometer and a FL-4500 fluorometer, respectively. Scanning electron microscope (SEM) images were acquired by ZEISS MERLIN compact (Germany). Relative fluorescence quantum yields were determined by using fluorescein (Φ = 0.90 in 0.1 M NaOH) and quinine sulfate dihydrate (Φ = 0.55 in 0.5 M H_2SO_4) as references. [1-3]

1.2 Synthetic procedures and characterization data

1.2.1 Synthesis of *trans*-BODIPY-DCH

Under N_2 atmosphere, 8-chloro-BODIPY[4] (200 mg, 883.2 μmol), (1R, 2R)-(-)-1,2-diaminocyclohexane (0.325 mL, 2.65 mmol) and 15.0 mL of anhydrous ethanol were placed in a round-bottom flask, then the reaction mixture was stirred at room temperature for 0.5 h. After reaction was completed, the resulting mixture was cooled down to room temperature, and the solvent was removed by vacuo. The residues were purified by chromatography on silica gel (DCM/methanol, v/v = 60:1) to give ***trans*-BODIPY-DCH** as light yellow solid (183 mg, yield: 68.1%). ^{1}H-NMR (500 MHz, DMSO-d_6) δ 7.39-7.38 (d, *J* = 4.0 Hz, 1H, Pyrrole-*H*), 7.33-7.32 (d, *J* = 4.0 Hz, 1H, Pyrrole-*H*), 7.21-7.20 (d, *J* = 4.0 Hz, 1H, Pyrrole-*H*), 7.05-7.04 (d, *J* = 4.0 Hz, 1H, Pyrrole-*H*), 6.46-6.44 (t, *J* = 4.0 Hz, 1H, Pyrrole-*H*), 6.30-6.28 (t, *J* = 4.0 Hz, 1H, Pyrrole-*H*), 5.15 (br s, 1H, N*H*-BODIPY), 3.88-3.85 (m, 1H, DCH-*H*), 3.02-2.99 (m, 1H, DCH-*H*), 2.12-2.07 (m, 1H, DCH-*H*), 1.95-1.93 (d, *J* = 8.0 Hz, 1H, DCH-*H*), 1.79-1.74 (m, 2H, DCH-*H*), 1.47-1.43 (m, 2H, DCH-*H*), 1.36-1.29 (m, 2H, DCH-*H*). ^{1}H-NMR (500 MHz, $CDCl_3$) δ 7.62 (s, 2H, Pyrrole-*H*), 7.07 (s, 2H, Pyrrole-*H*), 6.48 (s, 2H, Pyrrole-*H*), 3.70 (s, 1H, DCH-*H*), 2.75 (s, 1H, DCH-*H*), 2.45 (s, 1H, DCH-*H*), 2.07 (s, 1H, DCH-*H*), 1.87 (s, 2H, DCH-*H*), 1.43 (s, 2H, DCH-*H*), 1.35-1.32 (t, *J* = 8.0 Hz, 2H, DCH-*H*). ^{13}C-NMR (125 MHz, DMSO-d_6) δ/ppm 149.5, 130.9, 128.5, 128.2, 123.0, 120.7, 115.5, 113.4, 112.2, 62.6, 54.5, 33.3, 30.4, 24.8, 24.4. HR-MS (ESI): calculated for $[C_{15}H_{19}BF_2N_4 + H]^+$ 305.1744, found 305.1748.

1.2.2 Synthesis of *cis*-BODIPY-DCH

According to the above method, (1R,2S)-(-)-1,2-diaminocyclohexane (0.234 mL, 1.99 mmol) reacted with 8-chloro-BODIPY (200 mg, 883.2 μmol) affording to **cis-BODIPY-DCH** as yellow solid (121 mg, yield: 60.0%). ^{1}H-NMR (500 MHz, DMSO-d_6) δ 7.34-7.33 (d, J = 4.0 Hz, 1H, Pyrrole-*H*), 7.16-7.15 (d, J = 4.0 Hz, 1H, Pyrrole-*H*), 7.08-7.07 (d, J = 4.0 Hz, 1H, Pyrrole-*H*), 6.98-6.97 (d, J = 4.0 Hz, 1H, Pyrrole-*H*), 6.43-6.41 (t, J = 4.0 Hz, 1H, Pyrrole-*H*), 6.28-6.26 (t, J = 4.0 Hz, 1H, Pyrrole-*H*), 5.62 (br s, 1H, N*H*-BODIPY), 4.26-4.23 (m, 1H, DCH-*H*), 3.24–3.21 (t, J = 4.0 Hz, 1H, DCH-*H*), 1.92 – 1.88 (m, 1H, DCH-*H*), 1.83–1.78 (m, 1H, DCH-*H*), 1.75–1.71 (m, 2H, DCH-*H*), 1.65–1.60 (m, 2H, DCH-*H*), 1.46–1.41 (m, 2H, DCH-*H*). ^{1}H-NMR (500 MHz, $CDCl_3$) δ 8.48 (s, 1H, N*H*-BODIPY), 7.68 (s, 1H, Pyrrole-*H*), 7.52 (s, 1H, Pyrrole-*H*), 7.08 (s, 1H, Pyrrole-*H*), 7.02 (s, 1H, Pyrrole-*H*), 6.55 (s, 1H, Pyrrole-*H*), 6.43 (s, 1H, Pyrrole-*H*), 4.07-4.03 (m, 1H, DCH-*H*), 3.20-3.18 (t, J = 4.0 Hz, 1H, DCH-*H*), 2.19-2.15 (m, 1H, DCH-*H*), 1.89-1.88 (m, 1H, DCH-*H*), 1.77-1.71 (m, 2H, DCH-*H*), 1.54-1.50 (m, 2H, DCH-*H*), 1.46-1.42 (m, 2H, DCH-*H*). ^{13}C-NMR (125 MHz, DMSO-d_6) δ/ppm 147.9, 129.8, 129.4, 127.2, 123.7, 119.4, 113.3, 113.0, 111.8, 56.3, 50.1, 29.3, 26.1, 23.1, 20.3. HR-MS (ESI): calculated for $[C_{15}H_{19}BF_2N_4 + H]^+$ 305.1744, found 305.1739.

1.3 Spectral sensing properties of *trans*-BODIPY-DCH and *cis*-BODIPY-DCH in Solution.

To avoid direct usage of high toxic and uncontrollable gaseous phosgene, a steerable triphosgene was employed to *in situ* generate phosgene under the catalysis of triethylamine (TEA). ***Trans*-BODIPY-DCH** or ***cis*-BODIPY-DCH** stock solution (1.0 mM) were prepared in chloroform (HPLC grade). Before optical measurements, ***trans*-BODIPY-DCH** or ***cis*-BODIPY-DCH** solution was diluted to 5.0 μM in a 1.0 cm quartz cell with cover. First, we investigated the sensing performance of ***trans*-BODIPY-DCH** for phosgene. The assays were carried out by addition of triphosgene (0 − 5.0 μM) into ***trans*-BODIPY-DCH** solution (5.0 μM) with TEA (100.0 μM) at 25 °C. The UV-vis absorption and fluorescence spectra of ***trans*-BODIPY-DCH** solution (5.0 μM) were recorded after the addition of 0 – 5.0 μM of acetyl chloride (AC), triphosgene, diphosgene, oxalyl chloride (OC), benzoyl chloride (BzCl), $SOCl_2$, or diethyl chlorophosphate (DCP), respectively. In the same way, triphosgene or acyl chlorides were added to ***cis*-BODIPY-DCH** (5.0 μM) solution with TEA (100.0 μM) to explore its sensing performance for phosgene and acyl chlorides.

1.4 Selectivity test

We further studied the selectivity of ***trans*-BODIPY-DCH** (5.0 μM) for 10.0 μM diphosgene, dimethyl methylphosphonate (DMMP), diethyl chlorophosphate (DCP), tosyl chloride (TsCl), $POCl_3$, $SOCl_2$, HCl, benzoyl chloride (BzCl), oxalyl chloride (OC), acetyl chloride (AC), formaldehyde, methylglyoxal, acrolein, triphosgene and phosgene, respectively. The UV-vis absorption and fluorescence spectra of ***trans*-BODIPY-DCH** were obtained at room temperature (λ_{ex} = 430 nm). The fluorescence images were recorded under 365 nm UV light. For ***cis*-BODIPY-DCH**, selectivity assays were carried out by addition of above compounds (50.0 μM) into ***cis*-BODIPY-DCH** (5.0 μM) solution, respectively. The UV-vis absorption and fluorescence spectra of ***trans*-BODIPY-DCH** were measured in the same way. The fluorescence images were recorded under 365 nm UV light.

1.5 Time-dependent fluorescence response

We investigated the time-resolved fluorescence response of ***trans*-BODIPY-DCH** towards acyl chlorides, nerve agent mimics or phosgene in chloroform. Triphosgene (10.0 μM) was quickly added to the ***trans*-BODIPY-DCH** solution (5.0 μM) containing TEA (100.0 μM), and the fluorescence intensity was recorded and the ratio F_{524}/F_{478} was plotted as a function of time. In the same way, time-dependent fluorescence intensity of ***trans*-BODIPY-DCH** (5.0 μM) for other analytes (10.0 μM) were measured, including acetyl chloride (AC), triphosgene, diphosgene, oxalyl chloride (OC), benzoyl chloride (BzCl), $SOCl_2$, and diethyl chlorophosphate (DCP). Besides, we recorded continuous time-lapsed fluorescence images of ***trans*-BODIPY-DCH** for phosgene and acetyl chloride, respectively. Triphosgene (10.0 μM) was added to ***trans*-BODIPY-DCH** solution (5.0 μM) containing TEA (2.0 μL) in a glass bottle, and the fluorescence images were continuously captured using a mobile phone camera under 365 nm UV light. In the same manner, we also recorded the time-lapsed fluorescence images of ***trans*-BODIPY-DCH** (5.0 μM) in the presence of acetyl chloride (10.0 μM). The movies dealing with response rates of ***trans*-BODIPY-DCH** for phosgene and acetyl chloride are provided as movie 1. mp4; movie 2. mp4.

The sensing response rate of ***cis*-BODIPY-DCH** for phosgene and acetyl chloride (AC) were also investigated, respectively. After addition of triphosgene (50.0 μM) or acetyl chloride (50.0 μM), the time-lapsed fluorescence images of ***cis*-BODIPY-DCH** (5.0 μM) containing TEA (2.0 μL) were recorded by using a mobile phone camera under 365 nm UV light, which were shown in movie 3. mp4; movie 4. mp4.

1.6 Simultaneous sensing of phosgene and acyl chlorides.

A series of mixture solutions was prepared in HPLC grade chloroform, including AC (1.0 mmol)/triphosgene (0.33 mmol), DCP (1.0 mmol)/triphosgene (0.33 mmol), BzCl (1.0 mmol)/triphosgene (0.33 mmol) and $SOCl_2$ (1.0 mmol)/triphosgene (0.33 mmol). To simultaneously determine AC and phosgene, AC (0 - 5.0 μM) and triphosgene (0 – 1.7 μM) mixed solutions were added into ***trans*-BODIPY-DCH** (5.0 μM) solution with TEA (100.0 μM), and the UV-vis absorption and fluorescence spectra were recorded. Note, 0 - 1.7 μM of triphosgene can be catalyzed by triethylamine to form 0 - 5 μM phosgene. The test methods for other analytes are consistent with the above mentioned procedure.

1.7 Fabrication of test strips.

Melt-blown nonwovens (component: polypropylene) were cut into test strips (size: 1.0 × 2.0 cm). These test strips were immersed into ***trans*-BODIPY-DCH** (5.0 mM) solution, which were ultrasonicated for 15 min, and then suspended for 30 min. Finally, the test strips were taken out and dried naturally at room temperature to give ***trans*-BODIPY-DCH** -loaded nonwoven test strips.

Triphosgene stock solutions with different concentrations (6.8, 14.0, 34.0, 68.0, 135.0 and 270.0 μM) were prepared in chloroform. The stock solutions of AC, triphosgene, diphosgene, OC, BzCl, $SOCl_2$, and DCP were also prepared in chloroform, respectively. ***trans*-BODIPY-DCH** test strips were placed inside a bottle, and 10 μL of the above stock solution was added to 10 mL glass bottles, then the bottles were stored at 40 ^oC for 10 min to make the analytes vaporise. After exposure to gaseous analytes for 1 min, test strips were taken out in fume hood for recording fluorescence images by using a mobile phone camera under a 365 nm UV lamp, meanwhile 2.0 mL ethanol was immediately added to the glass bottle to destroy the residual analytes.

1.8 Fluorescence imaging by Using Confocal Microscopy.

***trans*-BODIPY-DCH** -loaded nonwoven test strips were exposed to analytes vapors (0-20 ppm) in 10 mL glass bottles for 1 min, and then taken out for imaging by using Nikon A1 laser-scanning confocal microscope. For blue channel, λ_{ex} = 402 nm, λ_{em} = 425−475 nm; For green channel, λ_{ex} = 488 nm, λ_{em} = 500−530 nm.

1.9 Cytotoxicity Assay.

Cytotoxicity of ***trans*-BODIPY-DCH** in L929 and HeLa cells were evaluated by using the Cell Counting Kit-8. L929 and HeLa cells were seeded 5000 cells per well in 96-well plates (Corning),

respectively. Cells were adherent-cultured in wells for 24 h and washed with 100 μL/well PBS, and then incubated with various concentrations of ***trans*-BODIPY-DCH** (2.5, 5.0, 10.0, 15.0, 20.0, and 25.0 μM) for 24 h. These cells were washed with serum-free DMEM twice and incubated with 100 μL/well serum-free DMEM containing 10% CCK-8 for 1 h. The absorbance at 450 nm was recorded by a plate reader to calculate the cell viability rate according to literature. [S5]

1.10 Investigation of sensing mechanism.

1.10.1 Synthesis of *trans*-BODIPY-ICO.

***trans*-BODIPY-DCH** (50 mg, 164 μmol), 8 μL triethylamine (55 μmol) and 10.0 mL of anhydrous chloroform were placed in a round bottom flask with a rubber stopper. Then, triphosgene (32 mg, 108 μmol) in 2.0 mL chloroform was added, and the mixture was stirred for 30 min at room temperature. After the reaction finished, 10 mL distilled water was added, and the solution was stirred at room temperature for 1 h. Then, the solution was suspended for 15 min, and the organic layer was collected. The solvent was evaporated under reduced pressure, and the residue was purified by silica gel column chromatography (eluent: CH_2Cl_2/ethanol = 100/1, v/v) to give ***trans*-BODIPY-ICO** as red solid (45 mg, yield: 83%). ^{1}H NMR (400 MHz, DMSO-*d*$_6$) δ 8.05 (s, 1H), 7.89-7.88 (d, *J* = 4.0 Hz, 2H), 7.46-7.45 (d, *J* = 4.0 Hz, 1H), 7.33-7.32 (d, *J* = 4.0 Hz, 1H), 6.68-6.66 (t, *J* = 4.0 Hz, 1H), 6.60-6.57 (t, *J* = 6.0 Hz, 1H), 4.12-4.06 (m, 1H), 3.45-3.40 (m, 1H), 2.00-1.94 (t, *J* = 12.0 Hz, 2H), 1.79-1.71 (t, *J* = 16.0 Hz, 2H), 1.54-1.46 (m, 2H), 1.43-1.33 (m, 2H). ^{13}C NMR (100 MHz, DMSO-*d*$_6$) δ/ppm 159.4, 143.3, 142.2, 141.6, 132.2, 130.7, 129.7, 128.1, 118.3, 117.7, 67.6, 59.0, 29.0, 27.2, 23.9, 23.6. HR-MS (ESI): calculated for $[C_{16}H_{17}BF_2N_4O + H]^+$ 331.1536, found 331.1538.

1.10.2 Synthesis of *trans*-BODIPY-DACO.

***trans*-BODIPY-DCH** (60 mg, 197 μmol) and 10.0 mL anhydrous chloroform were placed in a round bottom flask with a rubber stopper. Then, acetyl chloride (17 μL, 236 μmol) was added, and the mixture was stirred for 15 min at room temperature. After the reaction finished, 10 mL distilled water was added, and the solution was stirred at room temperature for 1 h. Then, the solution was suspended for 15 min, and the organic layer was collected. The solvent was evaporated under reduced pressure, and the residue was purified by silica gel column chromatography using CH_2Cl_2/ethyl acetate (40:1, v/v) as eluent to give ***trans*-BODIPY-DACO** as faint yellow solid (58 mg, yield: 85%). ^{1}H NMR (400 MHz, DMSO-*d*$_6$) δ 9.36-9.35 (d, *J* = 4.0 Hz, 1H), 8.19-8.17 (d, *J* = 8.0 Hz, 1H), 7.58 (s, 1H), 7.37-7.36 (d, *J* = 4.0 Hz, 1H), 7.35-7.34 (d, *J* = 4.0 Hz, 1H), 7.24-7.23 (d, *J* = 4.0 Hz, 1H), 6.59-6.57 (t, *J* = 4.0 Hz, 1H), 6.40-6.38 (t, *J* = 4.0 Hz, 1H), 4.08-3.96 (m, 2H), 2.22-2.19 (d, *J* = 6.0 Hz, 2H), 1.92-1.89

(d, J = 6.0 Hz, 2H), 1.78 (s, 2H), 1.74 (s, 3H), 1.66-1.55 (m, 2H), 1.45-1.32 (m, 2H). ^{13}C NMR (100 MHz, DMSO-d_6) δ/ppm 171.0, 148.3, 133.8, 131.2, 125.5, 123.0, 121.5, 117.4, 114.9, 113.5, 61.6, 52.0, 31.6, 29.9, 24.8, 23.9, 23.0 . HR-MS (ESI): calculated for $[C_{17}H_{21}BF_2N_4O + H]^+$ 347.1849, found 347.1853.

1.11 X-ray single crystallography

The single crystals of ***trans*-BODIPY-DCH**, *trans*-BODIPY-ICO and *trans*-BODIPY-DACO were obtained in dichloromethane and n-hexane by slowly evaporating solvent from the saturated solution. The detail structures information were uploaded to The Cambridge Crystallographic Data Centre (CCDC) and the data can be obtained free of charge via www.ccdc.cam.ac.uk/structures. ***trans*-BODIPY-DCH** (CCDC number: 2094769), *trans*-BODIPY-ICO (CCDC number: 2094607) and *trans*-BODIPY-DACO (CCDC number: 2094606).

2. The pK_a prediction of *trans*-BODIPY-DCH

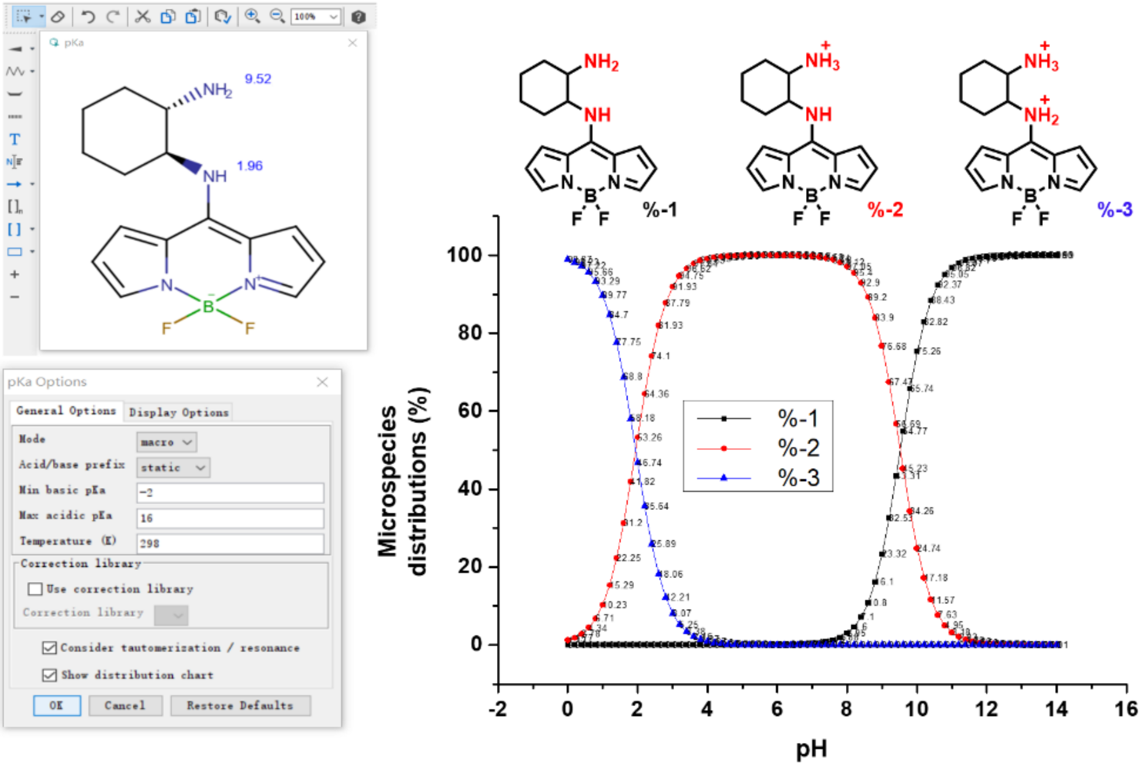

Figure S1. The pK_a prediction of ***trans*-BODIPY-DCH** by using MarvinSktech 20.8 with the micro mode.

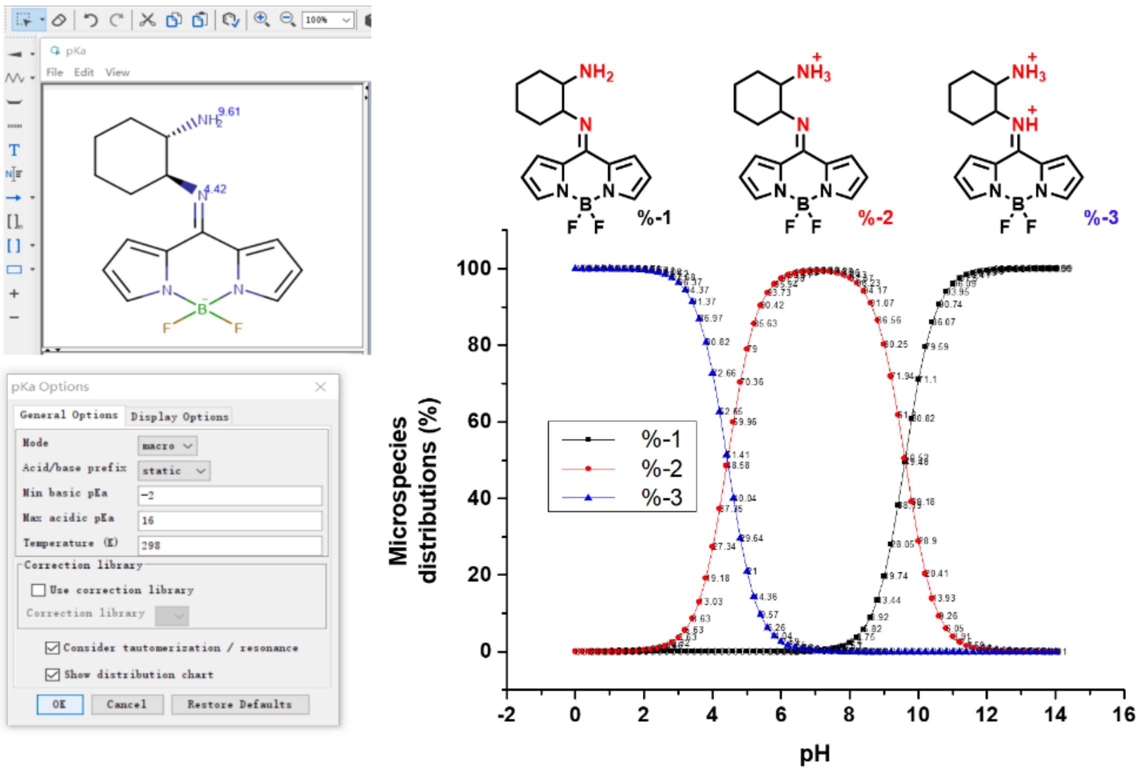

Figure S2. The pK_a prediction of ***trans*-BODIPY-DCH** tautomer by using MarvinSktech 20.8 with the macro mode.

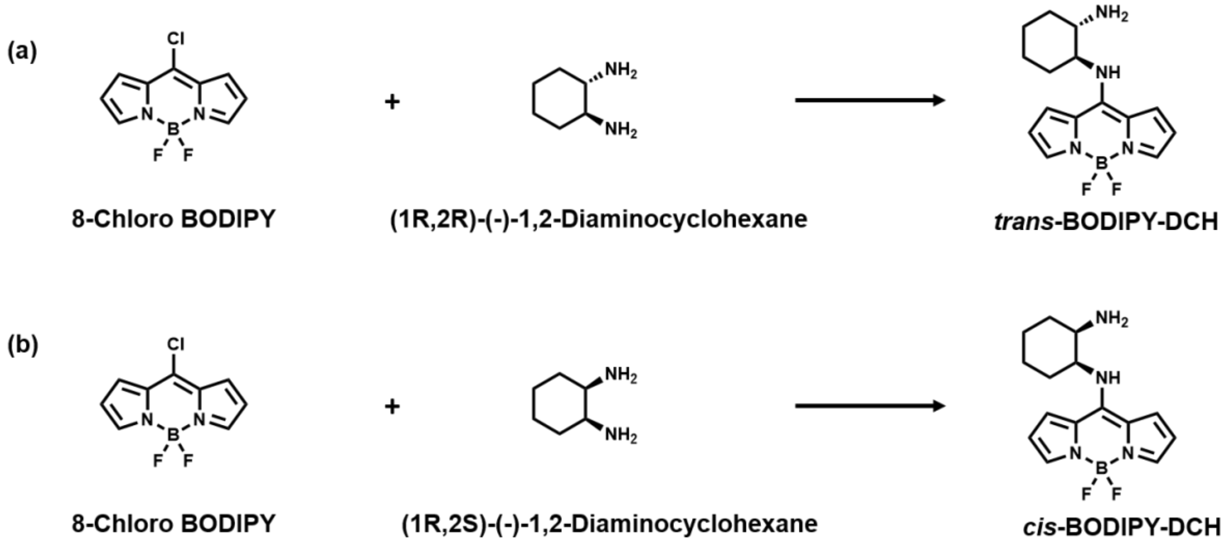

Figure S3. Synthesis of ***trans*-BODIPY-DCH** and ***cis*-BODIPY-DCH**.

3. Simultaneous sensing of diverse analytes with trans-BODIPY-DCH

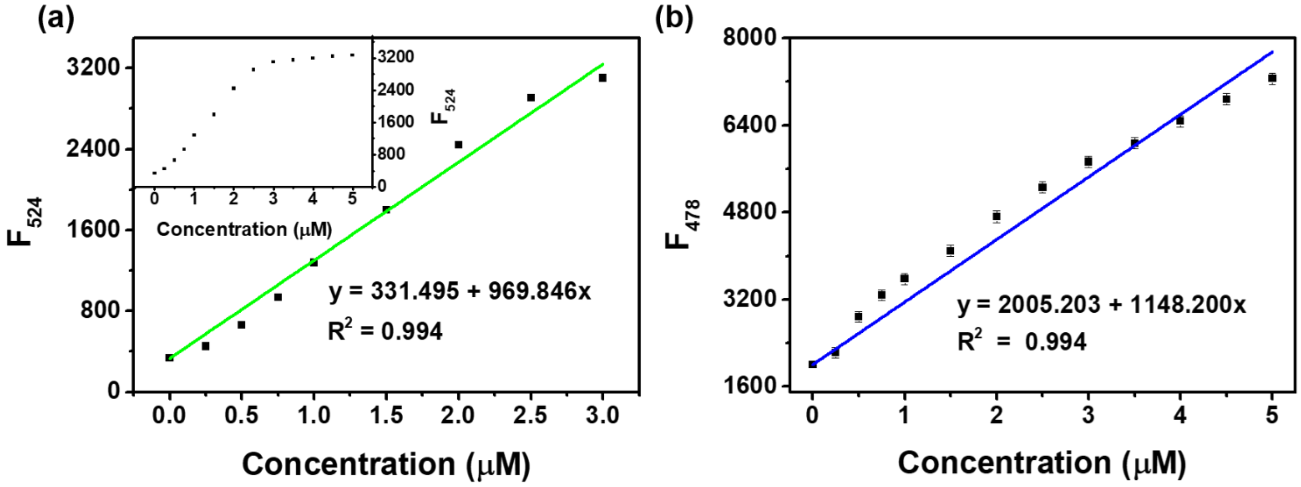

Figure S4. Fluorescence intensity linear correlation between ***trans*-BODIPY-DCH** (5.0 μM) and concentrations of (a) triphosgene (0 - 5.0 μM)/TEA (100.0 μM) and (b) acetyl chloride (5.0 μM) in $CHCl_3$. λ_{ex} = 430 nm, slits: 2.5 nm/5.0 nm, error bars are ± SD, n=3.

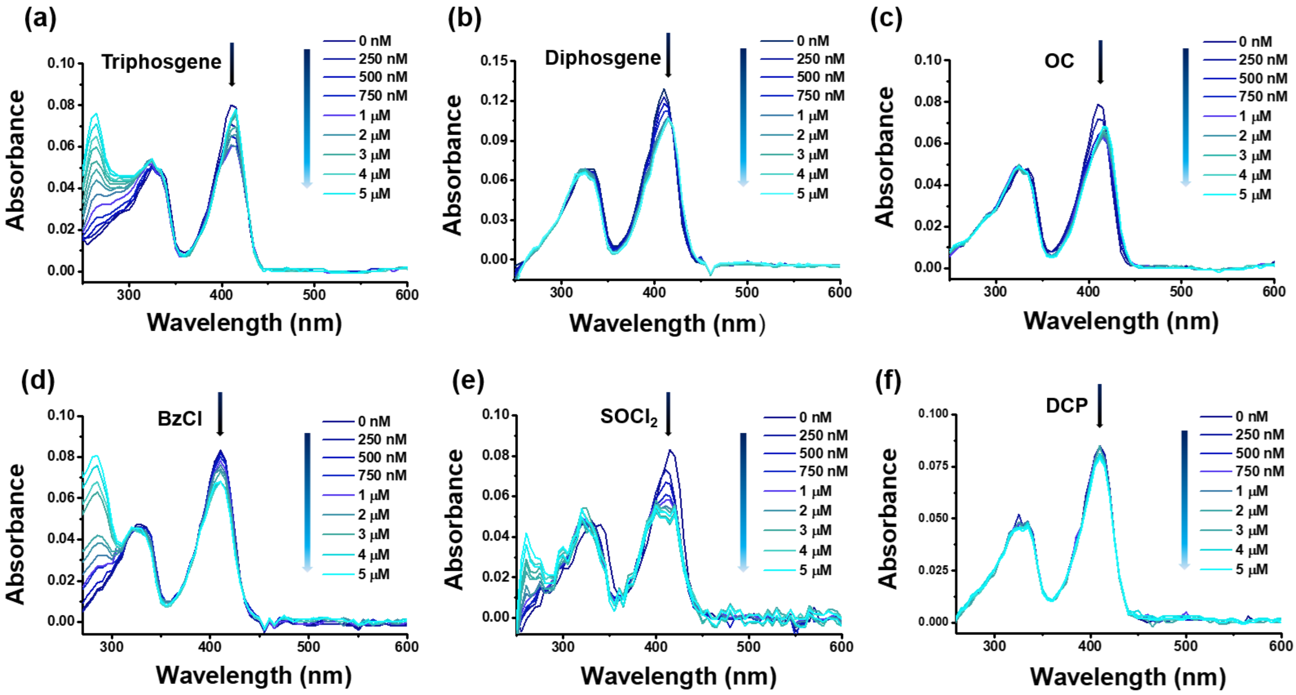

Figure S5. UV-vis absorption spectra responses of ***trans*-BODIPY-DCH** (5.0 μM) towards incremental analytes (0 - 5.0 μM), including: (a) triphosgene, (b) diphosgene, (c) OC, (d) BzCl, (e) $SOCl_2$ and (f) DCP in chloroform.

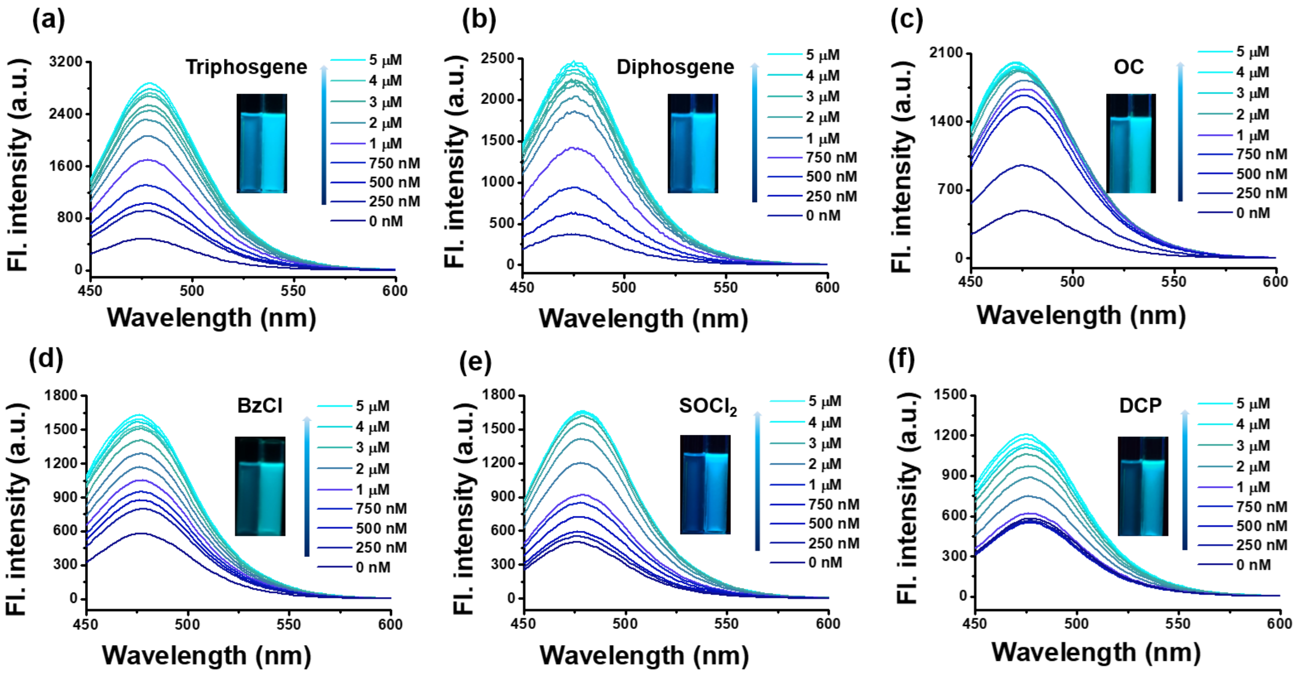

Figure S6. Fluorescence spectra changes of ***trans*-BODIPY-DCH** (5.0 μM) after addition of incremental analyte (0 - 5.0 μM): (a) triphosgene, (b) diphosgene, (c) OC, (d) BzCl, (e) $SOCl_2$ and (f) DCP in chloroform. Inset: fluorescence images of ***trans*-BODIPY-DCH** solution (5.0 μM) in the presence of analyte (5.0 μM) under 365 nm UV light. λ_{ex} = 430 nm, slits: 2.5 nm/2.5 nm.

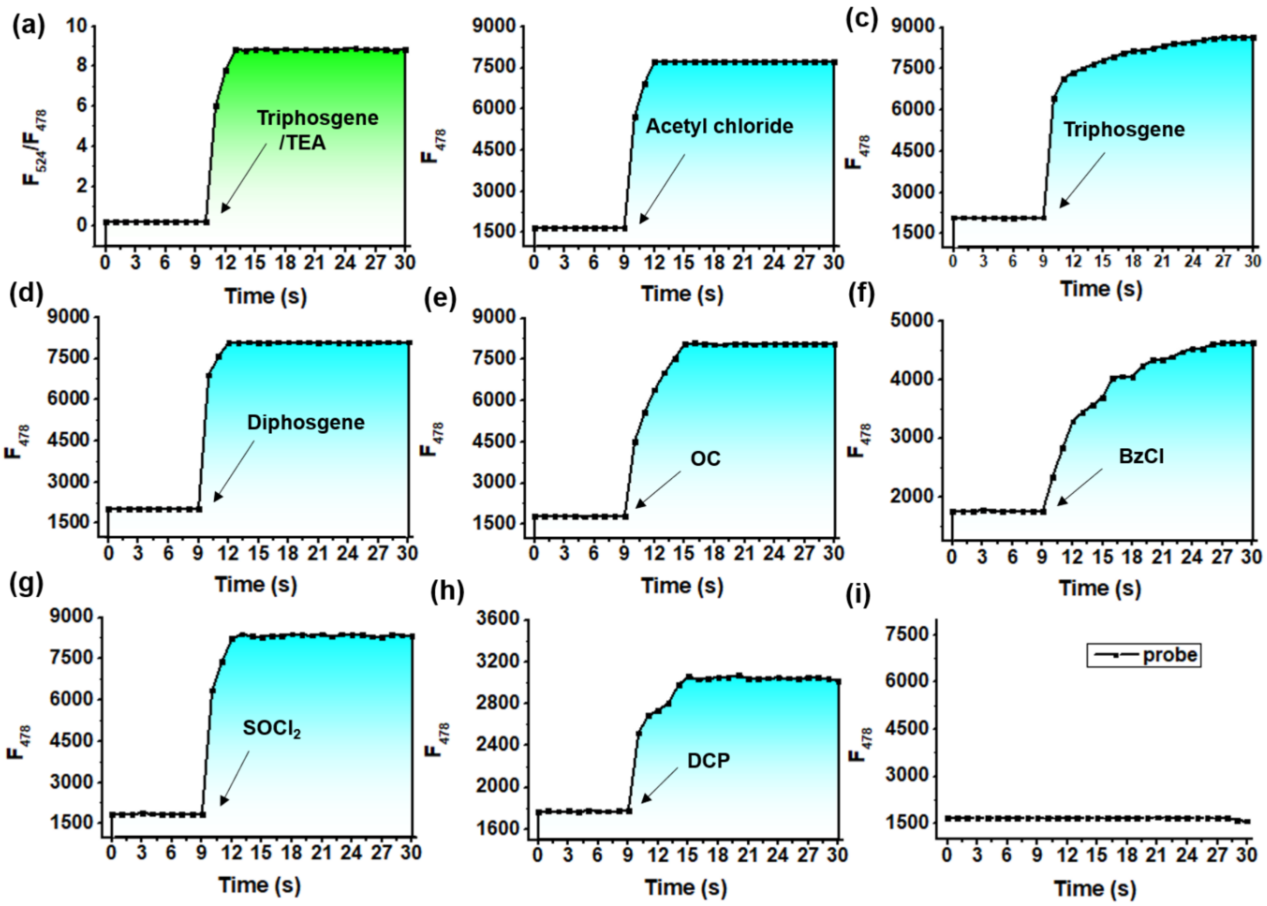

Figure S7. Time-resolved fluorescence response of ***trans*-BODIPY-DCH** solution (5.0 μM) to (a) triphosgene (10.0 μM)/TEA (100.0 μM), (b) acetyl chlorides, (c) triphosgene, (d) diphosgene, (e) OC, (f) BzCl, (g) $SOCl_2$, (h) DCP and (e) blank in $CHCl_3$. λ_{ex} = 430 nm, slits:2.5 nm/5.0 nm.

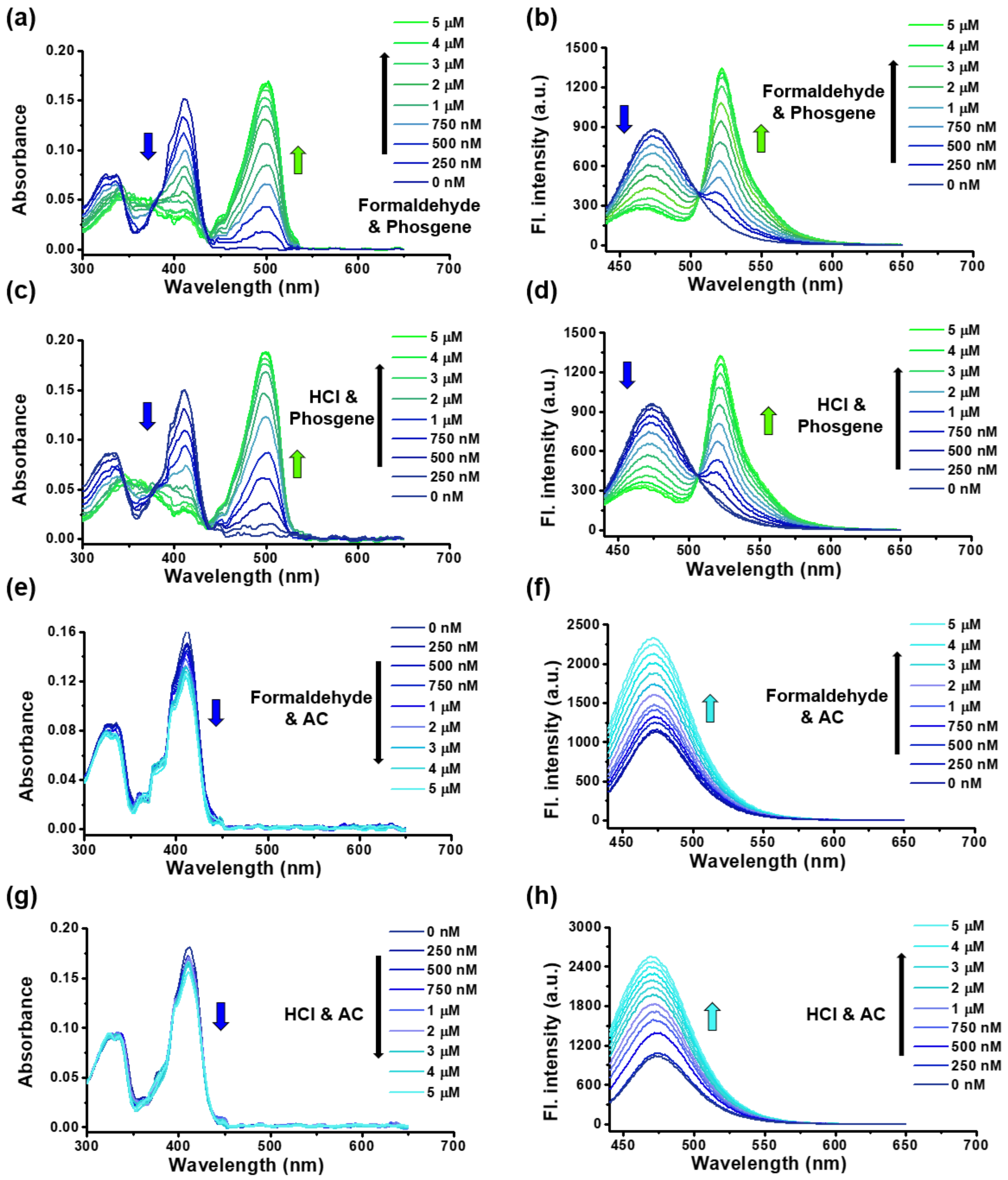

Figure S8. UV-vis absorption spectra and fluorescence spectra changes of ***trans*-BODIPY-DCH** (5.0 μM)/TEA (100.0 μM) in chloroform after the addition of incremental analytes mixture (0 - 5.0 μM). (a, b) Formaldehyde and phosgene, (c, d) HCl and phosgene, (e, f) formaldehyde and AC, (g, h) HCl and AC. Notes: analytes mixture solution contained triphosgene (0 – 1.7 μM, calculated to 0 - 5.0 μM of phosgene) and other analytes (0 - 5.0 μM). λ_{ex} = 430 nm, slits: 2.5 nm/2.5 nm.

4. The spectral response of *cis*-BODIPY-DCH towards some analytes

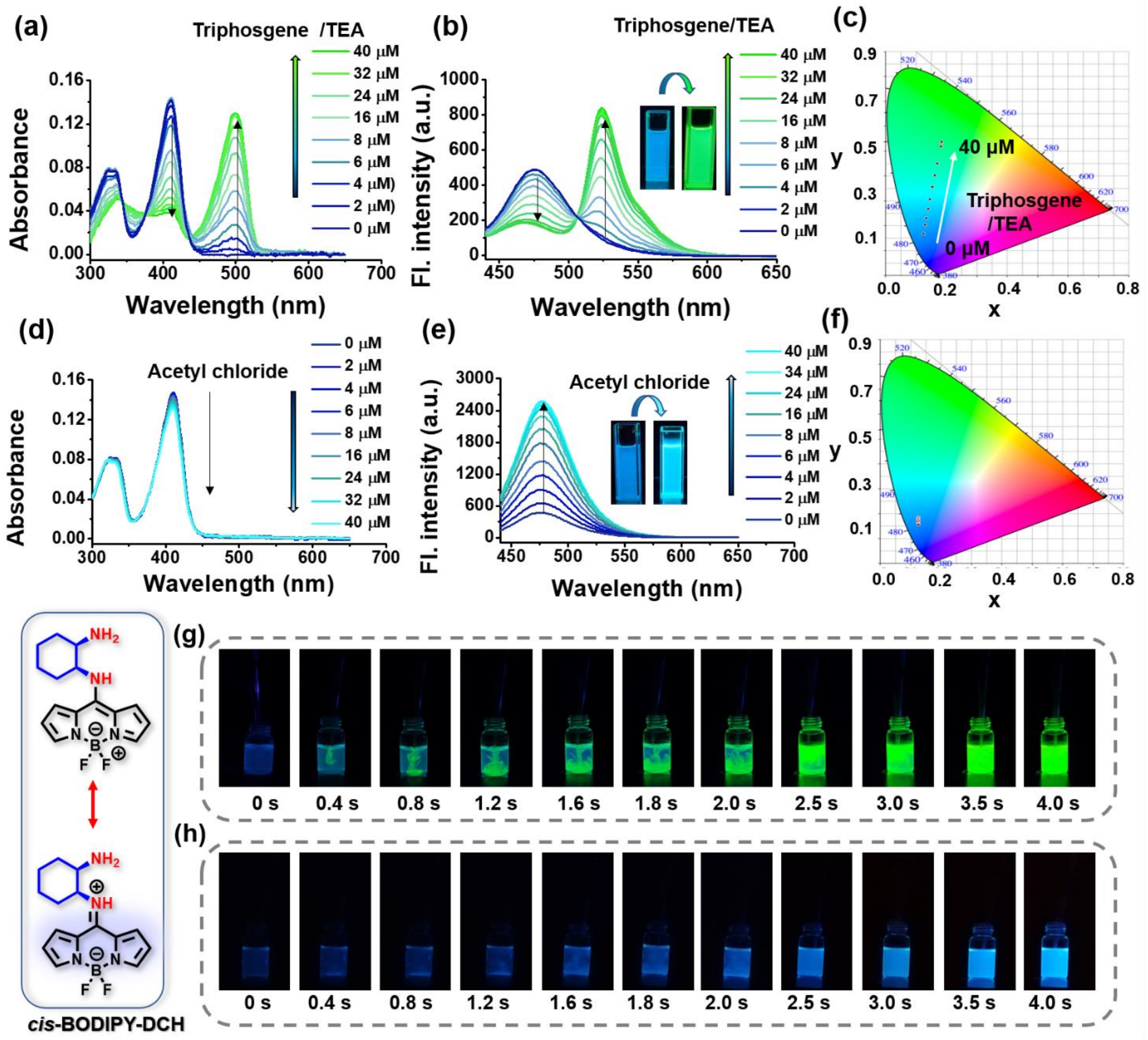

Figure S9. UV-vis absorption and fluorescence spectra changes of ***cis*-BODIPY-DCH** (5.0 μM) after the addition of incremental (a-b) triphosgene (0-40.0 μM)/TEA (100.0 μM) and (d-e) acetyl chloride (0-40.0 μM). (c) (f) CIE1931 coordinates of ***cis*-BODIPY-DCH** (5.0 μM) from (b) and (e), respectively. Time-lapsed fluorescence response images of ***cis*-BODIPY-DCH** (5.0 μM) towards (g) phosgene (50.0 μM) or (h) acetyl chloride (50.0 μM). λ_{ex} = 430 nm, slits: 2.5 nm/2.5 nm.

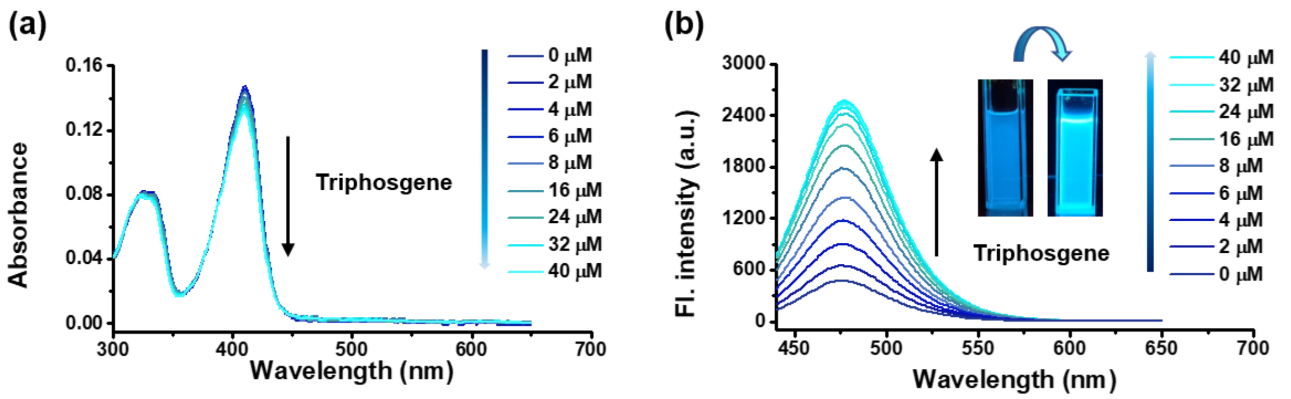

Figure S10. (a) UV-vis absorption spectra and (b) fluorescence spectra changes of ***cis*-BODIPY-DCH** (5.0 μM)/TEA (100 μM) in chloroform after the addition of incremental triphosgene (0 - 5.0 μM) in chloroform. λ_{ex} = 430 nm, slits: 2.5 nm/2.5 nm.

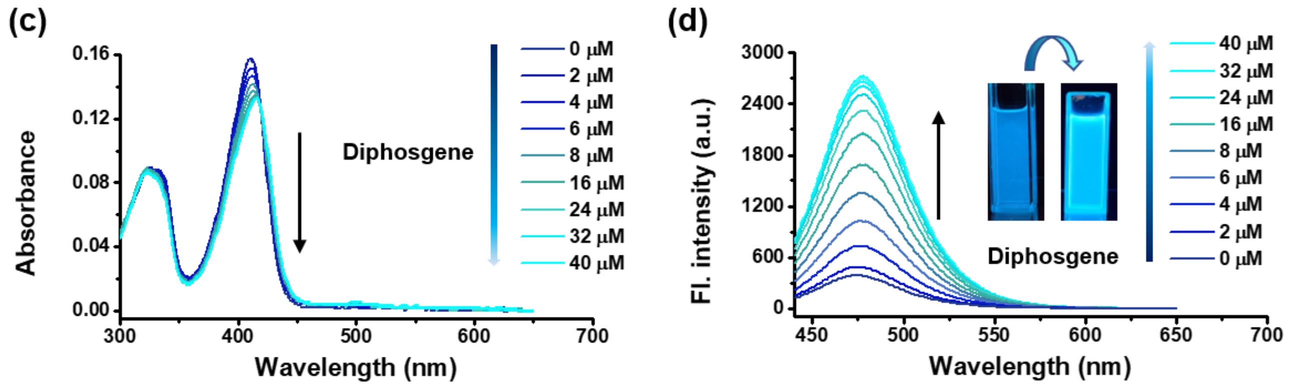

Figure S11. (a) UV-vis absorption spectra and (b) fluorescence spectra changes of ***cis*-BODIPY-DCH** (5.0 μM)/TEA (100 μM) after the addition of incremental diphosgene (0 - 5.0 μM) in chloroform. λ_{ex} = 430 nm, slits: 2.5 nm/2.5 nm.

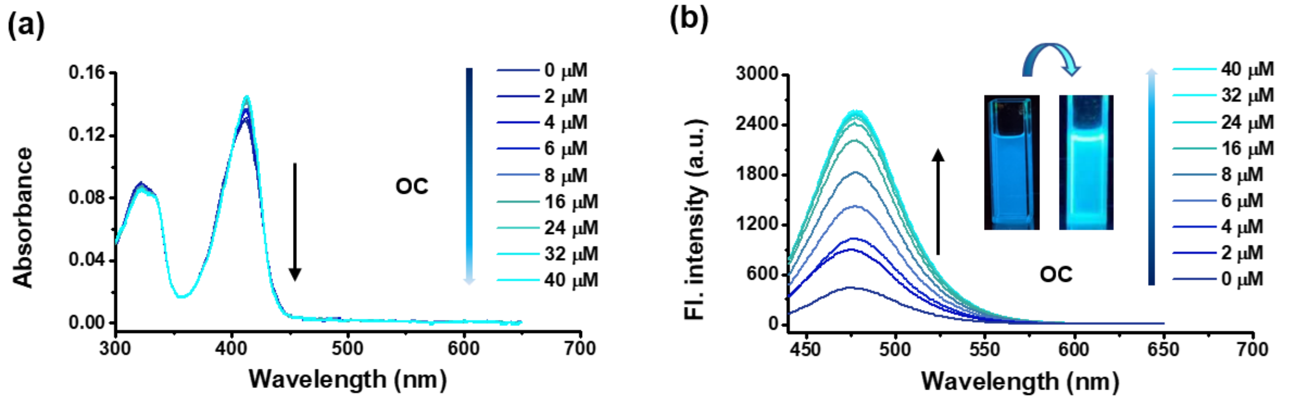

Figure S12. (a) UV-vis absorption spectra and (b) fluorescence spectra changes of ***cis*-BODIPY-DCH** (5.0 μM) after the addition of incremental oxalyl chloride (0 - 5.0 μM) in chloroform. λ_{ex} = 430 nm, slits: 2.5 nm/2.5 nm.

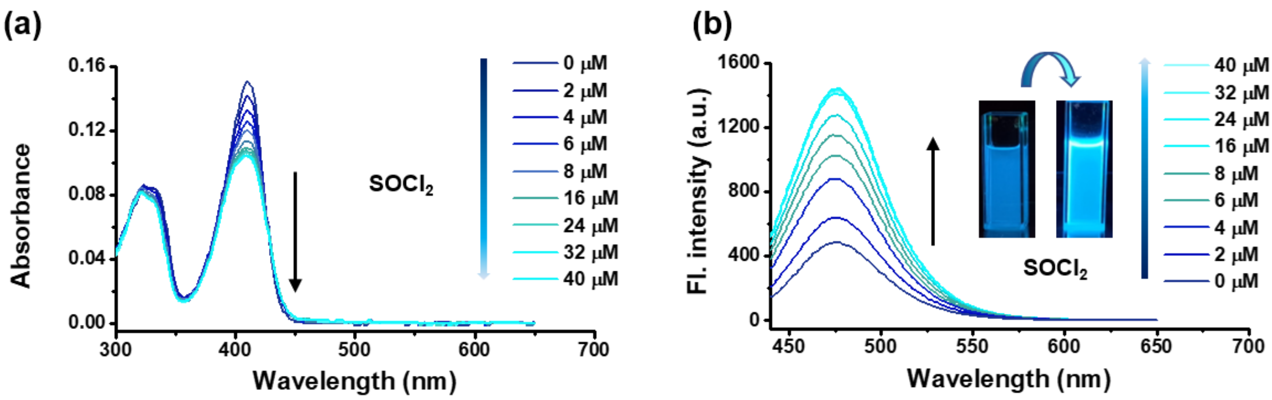

Figure S13. (a) UV-vis absorption spectra and (b) fluorescence spectra changes of ***cis*-BODIPY-DCH** (5.0 μM) after the addition of incremental $SOCl_2$ (0 - 5.0 μM) in chloroform. λ_{ex} = 430 nm, slits: 2.5 nm/2.5 nm.

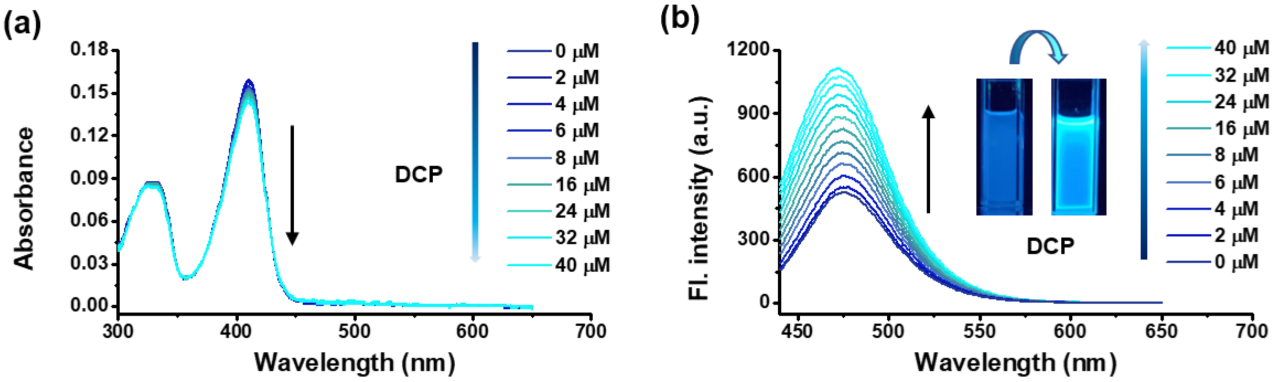

Figure S14. (a) UV-vis absorption spectra and (b) fluorescence spectra changes of ***cis*-BODIPY-DCH** (5.0 μM) after the addition of incremental DCP (0 - 5.0 μM) in chloroform. λ_{ex} = 430 nm, slits: 2.5 nm/2.5 nm.

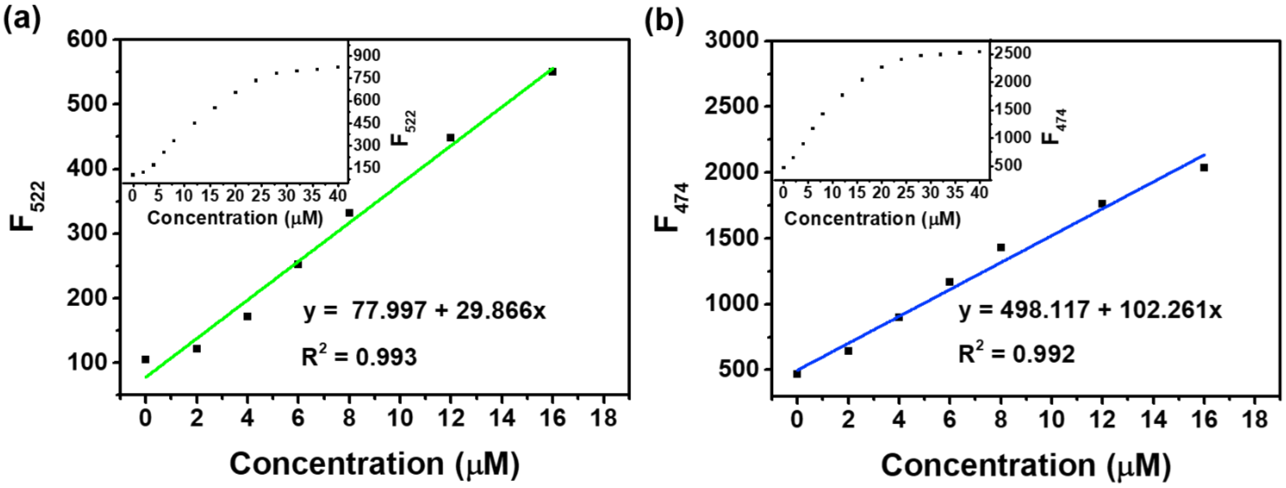

Figure S15. Fluorescence intensity linear correlation between ***cis*-BODIPY-DCH** (5.0 μM) and concentrations of (a) triphosgene (0 - 40.0 μM)/TEA (100.0 μM) and (b) acetyl chloride (0 - 40.0 μM) in $CHCl_3$. λ_{ex} = 430 nm, slits: 2.5 nm/2.5 nm, error bars are ± SD, n = 3.

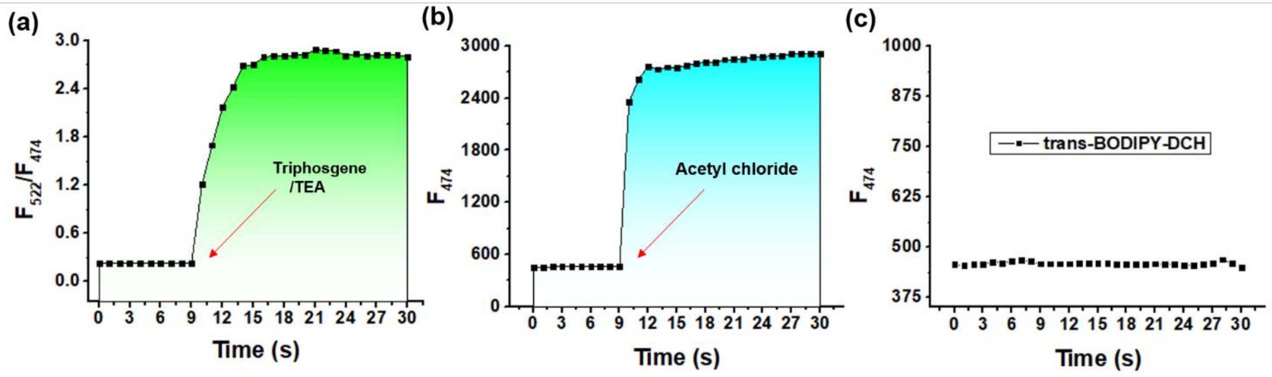

Figure S16. Time-resolved fluorescence response of ***cis*-BODIPY-DCH** (5.0 μM) towards (a) triphosgene (10.0 μM)/ TEA (100.0 μM), (b) acetyl chlorides (10.0 μM), and (c) blank in $CHCl_3$.

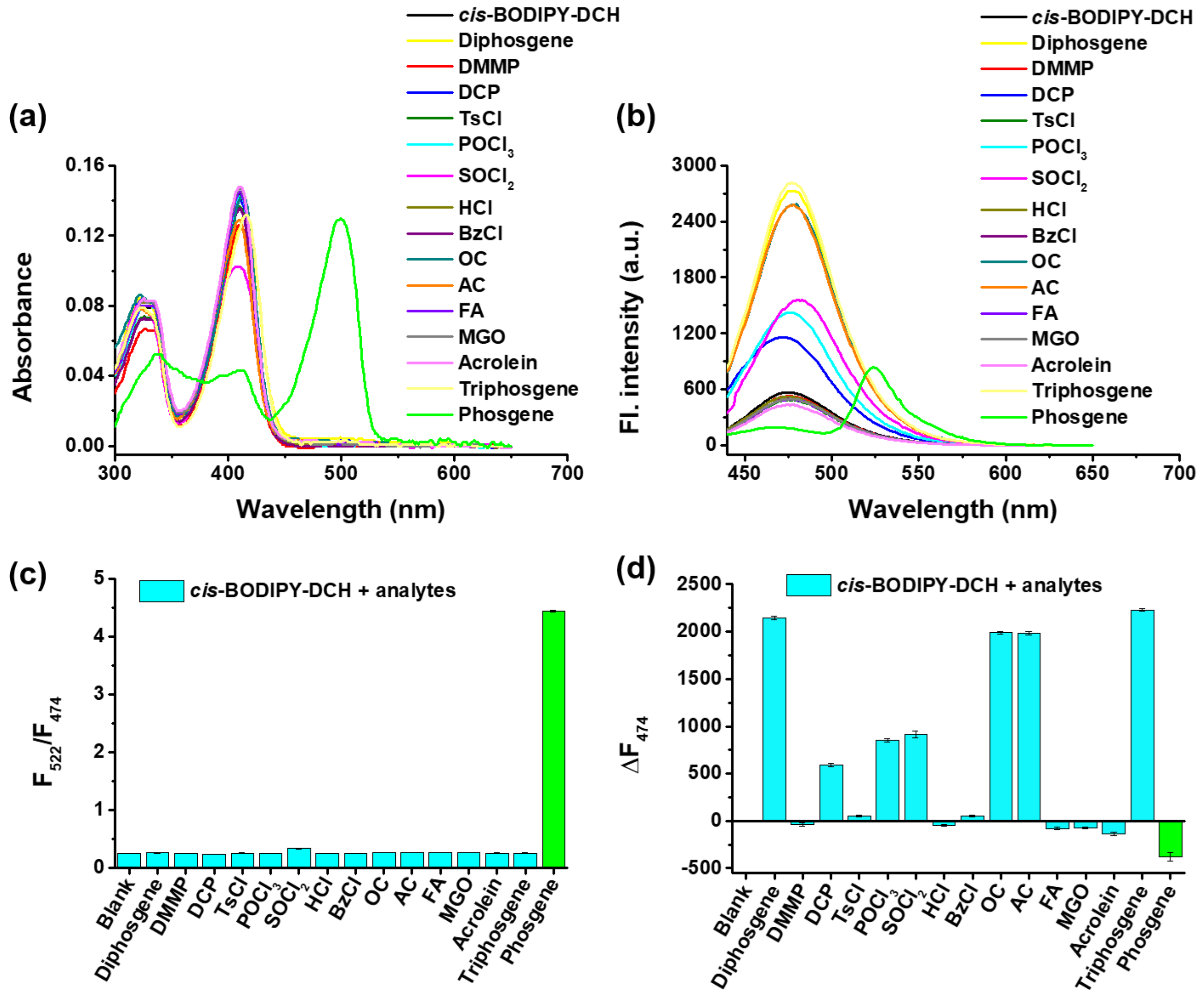

Figure S17. (a) UV-vis absorption and (b) fluorescence spectra of ***cis*-BODIPY-DCH** (5.0 μM) in the presence of different analytes in chloroform. (c) Ratio fluorescence intensity changes (F_{522}/F_{474}) of ***cis*-BODIPY-DCH** (5.0 μM) after the addition of analytes (10.0 μM). (d) Fluorescence intensity changes at 474 nm (ΔF_{474}) of ***cis*-BODIPY- DCH** (5.0 μM) after the addition of analytes (10.0 μM). λ_{ex} = 430 nm, slits: 2.5 nm/2.5 nm. Error bars are ± SD, n=3.

5. X-ray single crystallography

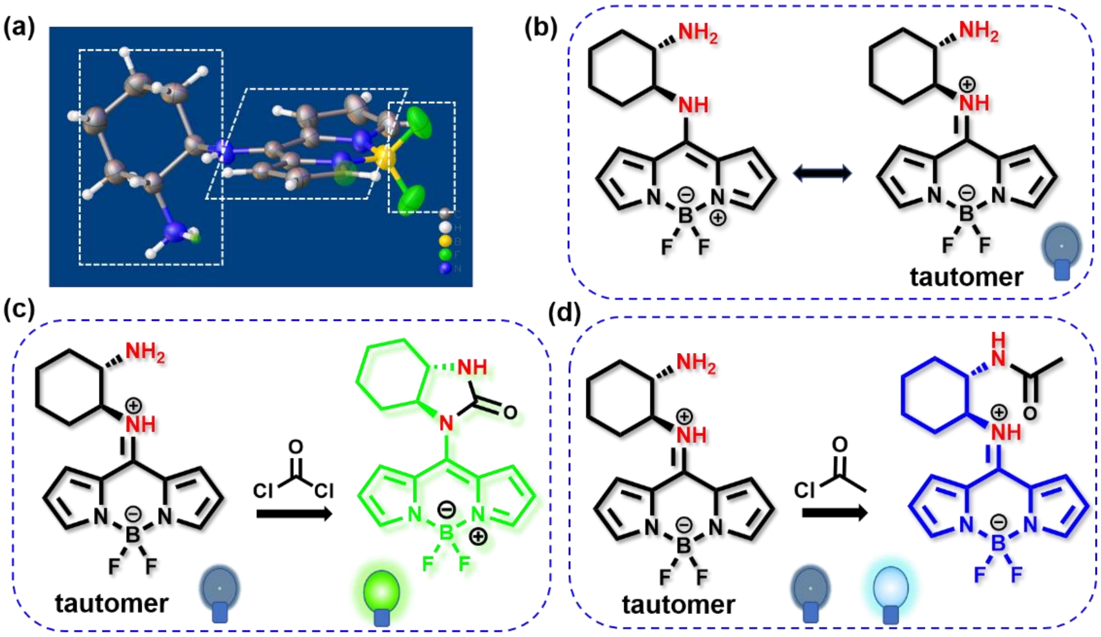

Figure S18. (a) The optimized geometry of ***trans*-BODIPY-DCH**. (b) ***Trans*-BODIPY-DCH** and its tautomer. (c) ***Trans*-BODIPY-DCH** coupled with phosgene by a nucleophilic substitution and isomerization from its tautomer. (d) ***trans*-BODIPY-DCH** coupled with acetyl chloride in the tautomer.

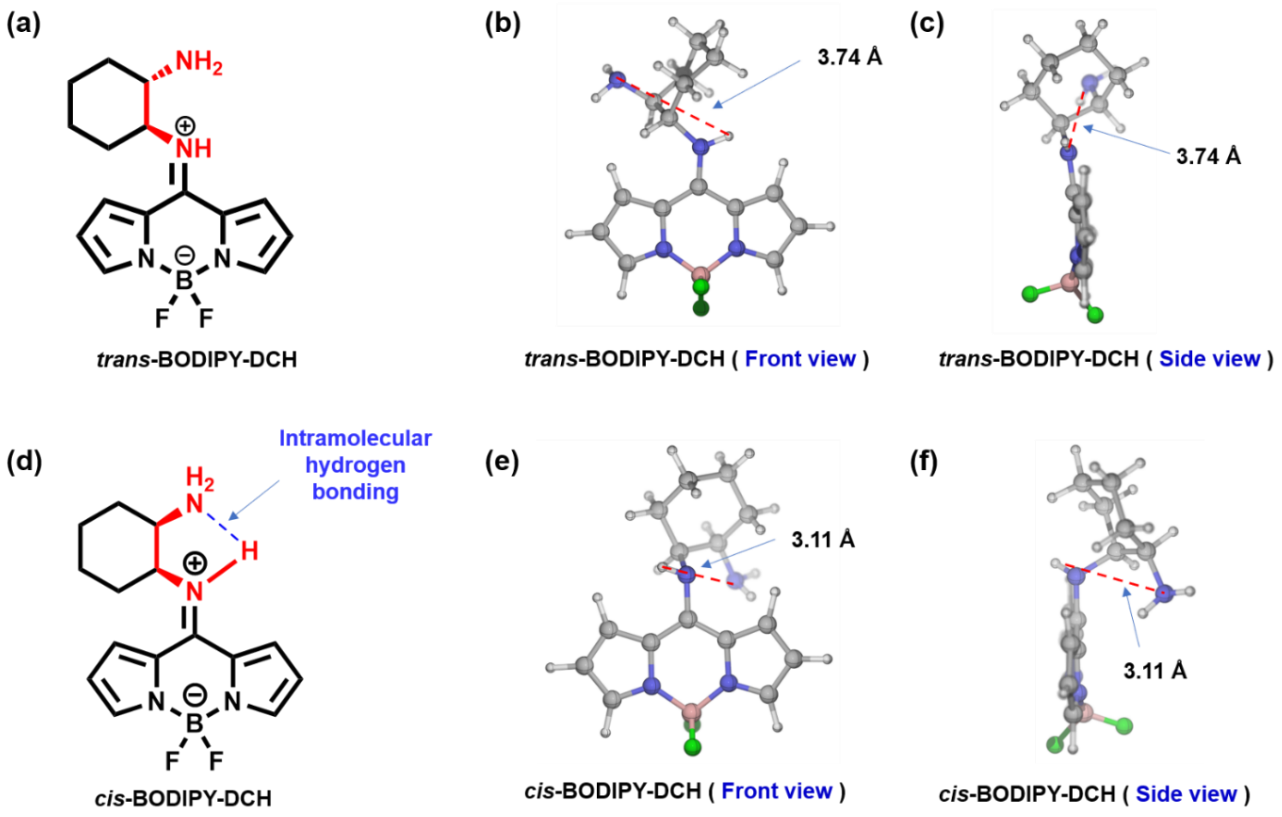

Figure S19. Optimized structures of ***trans*-BODIPY-DCH** and ***cis*-BODIPY- DCH**.

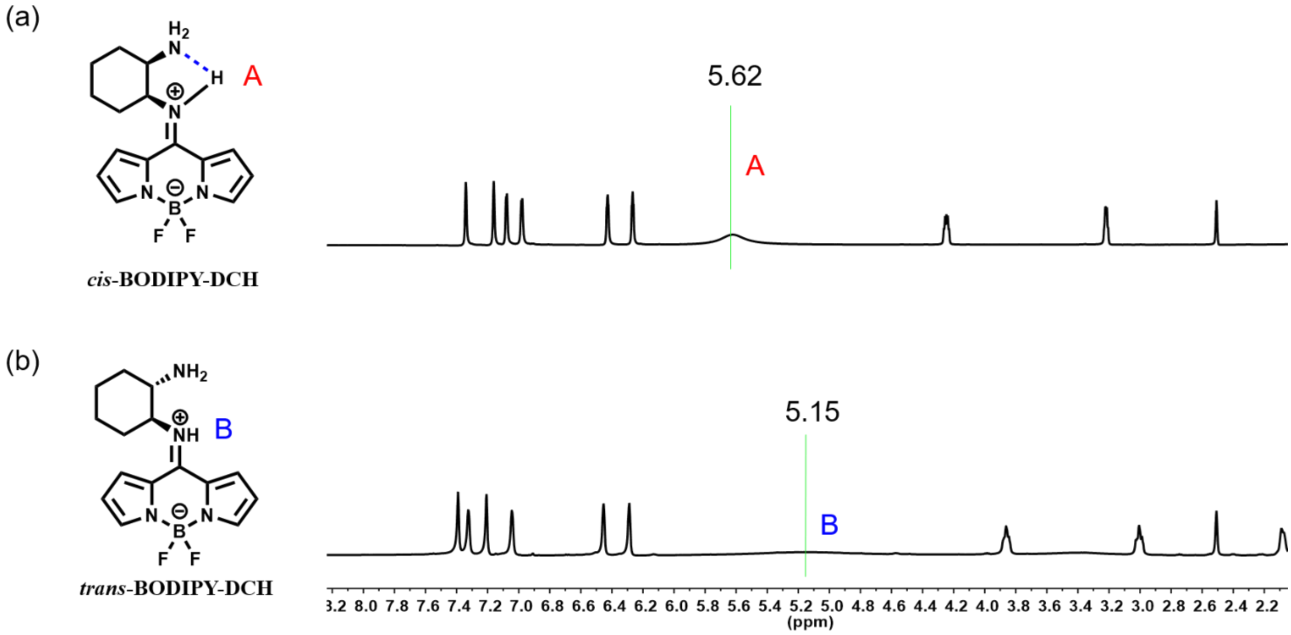

Figure S20. ^{1}H NMR spectrum of (a) ***trans*-BODIPY-DCH** and (b) ***cis*-BODIPY-DCH** in DMSO-d_6 (500 MHz).

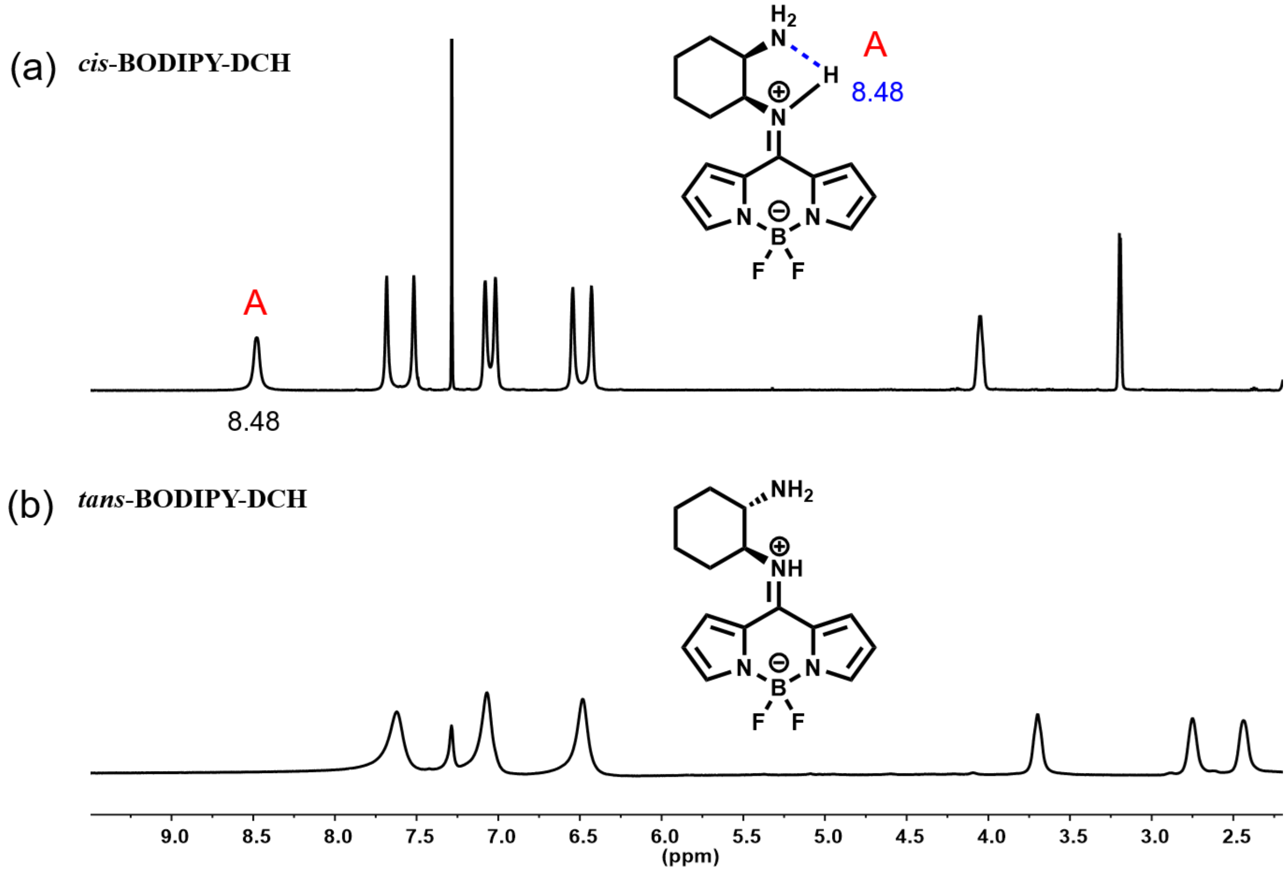

Figure S21. ^{1}H NMR spectrum of (a) ***trans*-BODIPY-DCH** and (b) ***cis*-BODIPY-DCH** in $CDCl_3$ (500 MHz).

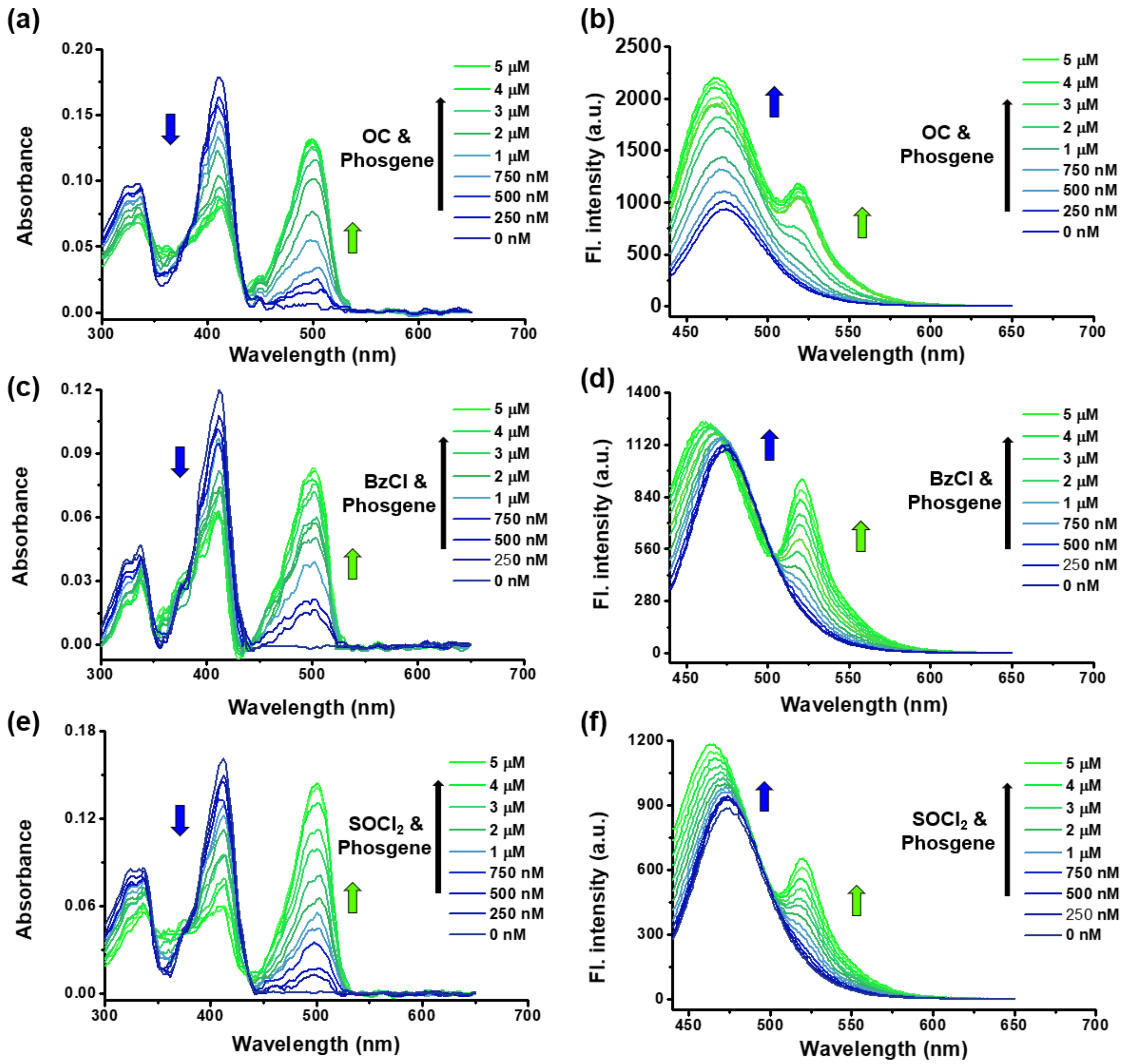

Figure S22. UV-vis absorption spectra and fluorescence spectra changes of ***trans*-BODIPY-DCH** (5.0 μM)/TEA (100.0 μM) in chloroform after the addition of incremental analytes mixture (0 - 5.0 μM). (a), (b) OC and phosgene, (c), (d) BzCl and phosgene, (e), (f) $SOCl_2$ and phosgene. Notes: analytes mixture solution contained triphosgene (0 – 1.7 μM, calculated to 0 - 5.0 μM of phosgene) and other analytes (0 - 5.0 μM). λ_{ex} = 430 nm, slits: 2.5 nm/2.5 nm.

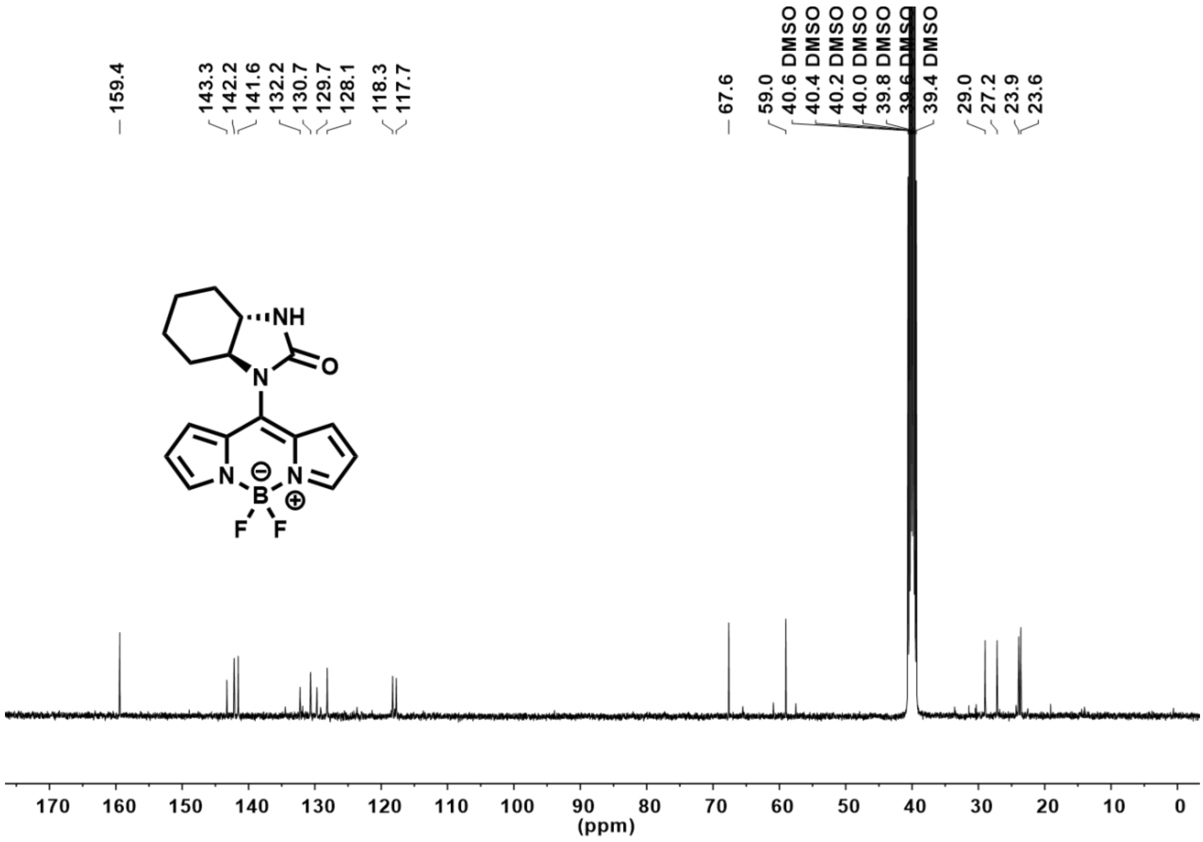

Figure 23. ^{13}C NMR spectrum of *trans*-BODIPY-ICO in DMSO-d_6 (125 MHz).

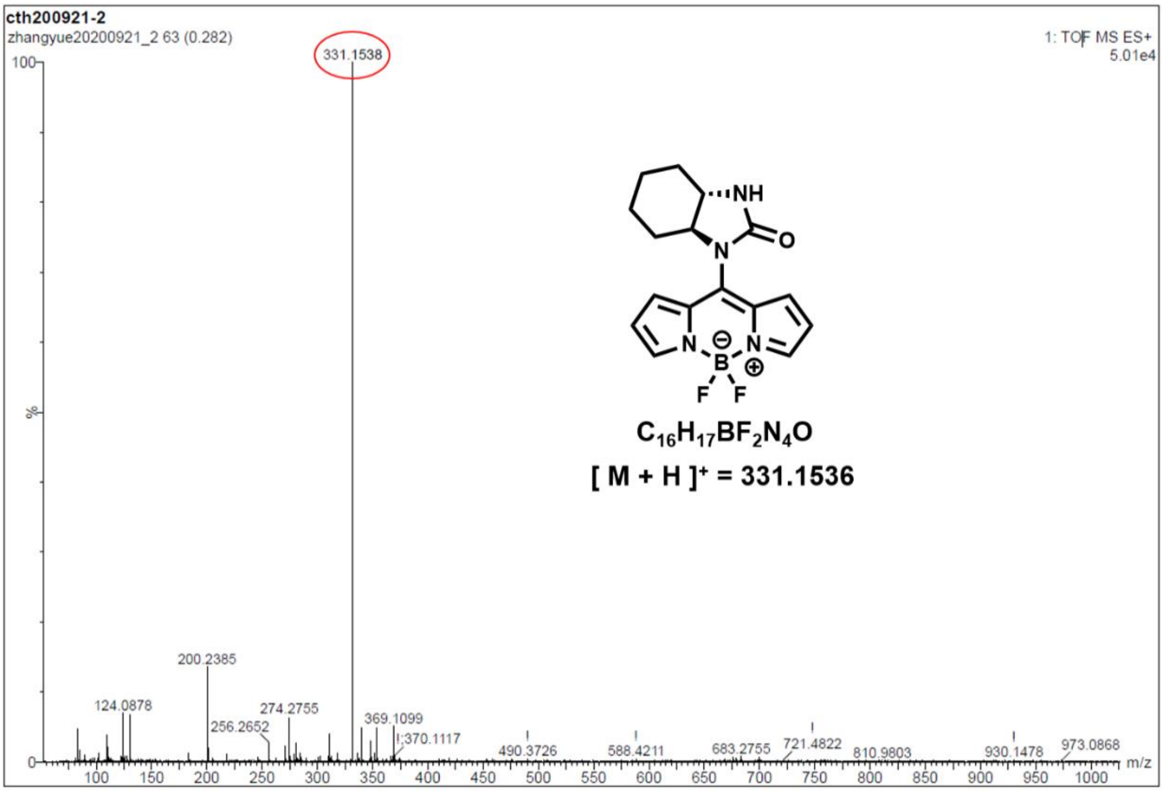

Figure S24. HR-MS spectra of *trans*-BODIPY-ICO.

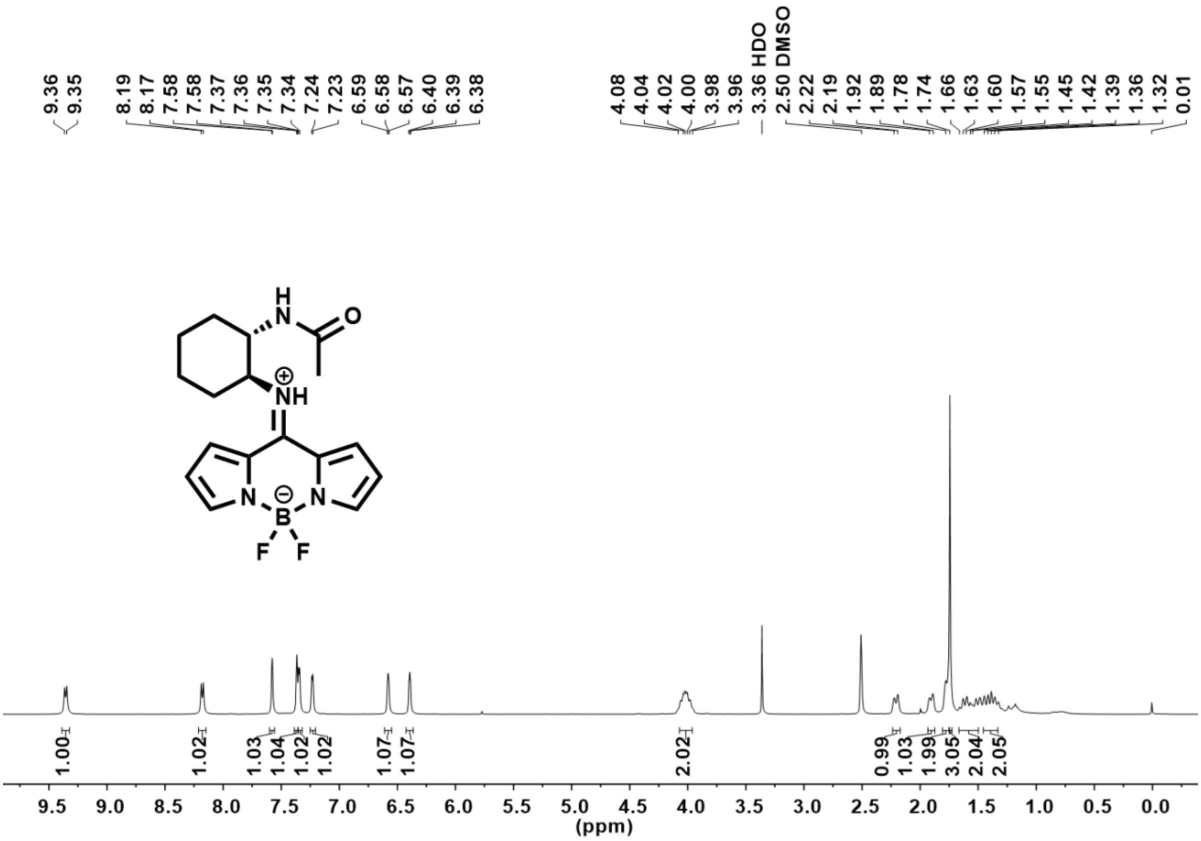

Figure S25. ^{1}H NMR spectrum of *trans*-BODIPY-DACO in DMSO-d_6 (500 MHz).

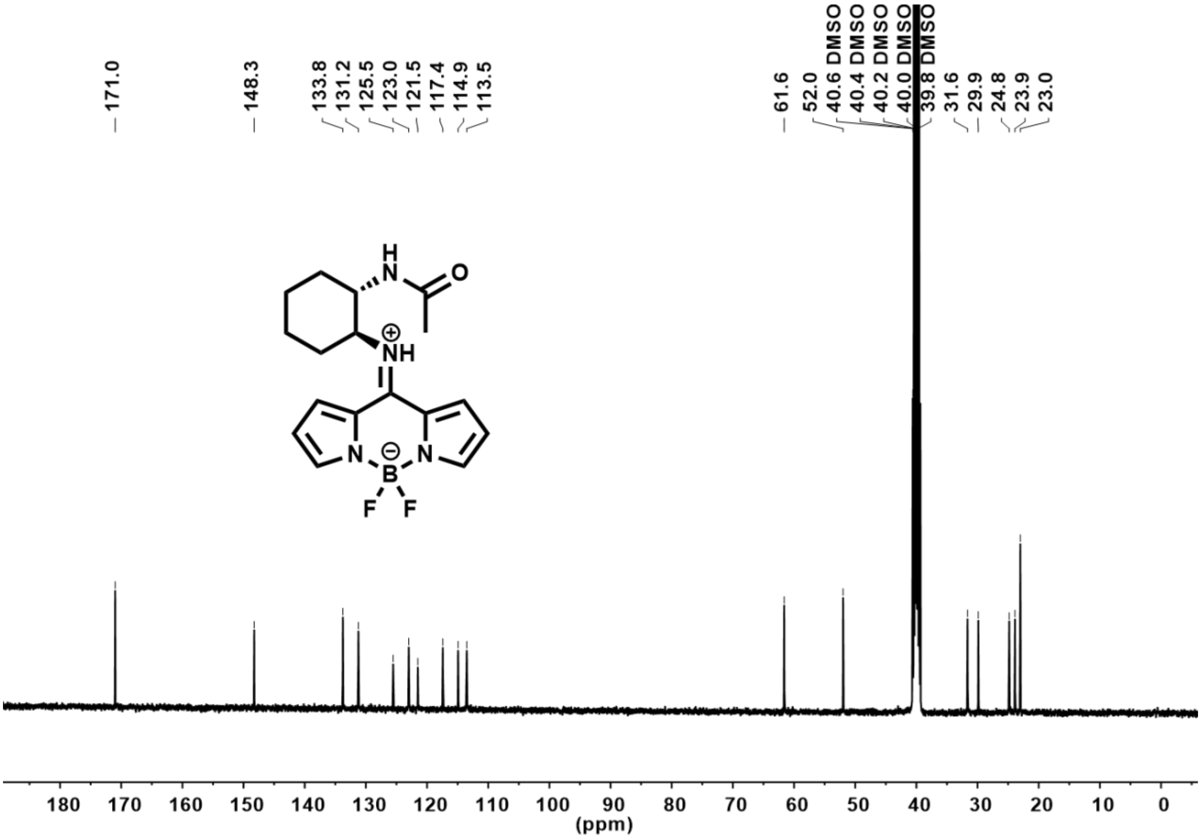

Figure S26. ^{13}C NMR spectrum of *trans*-BODIPY-DACO in DMSO-d_6 (125 MHz).

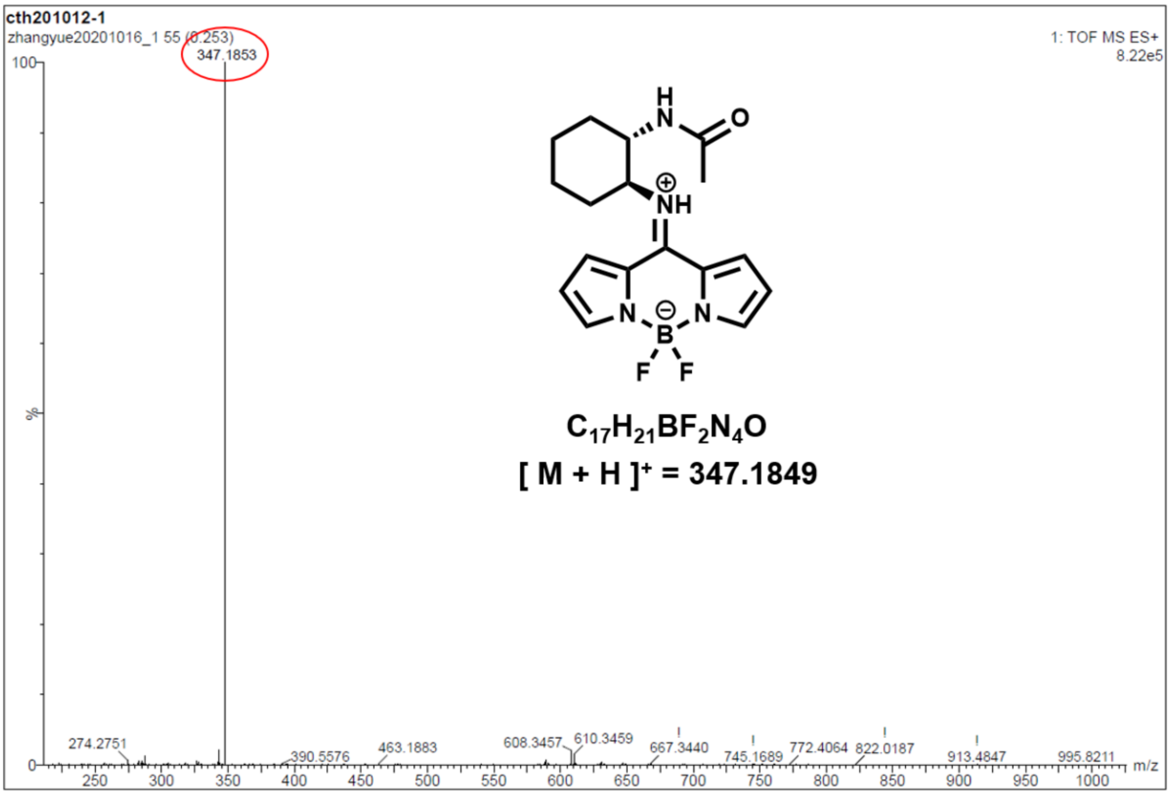

Figure S27. HR-MS spectra of *trans*-BODIPY-DACO.

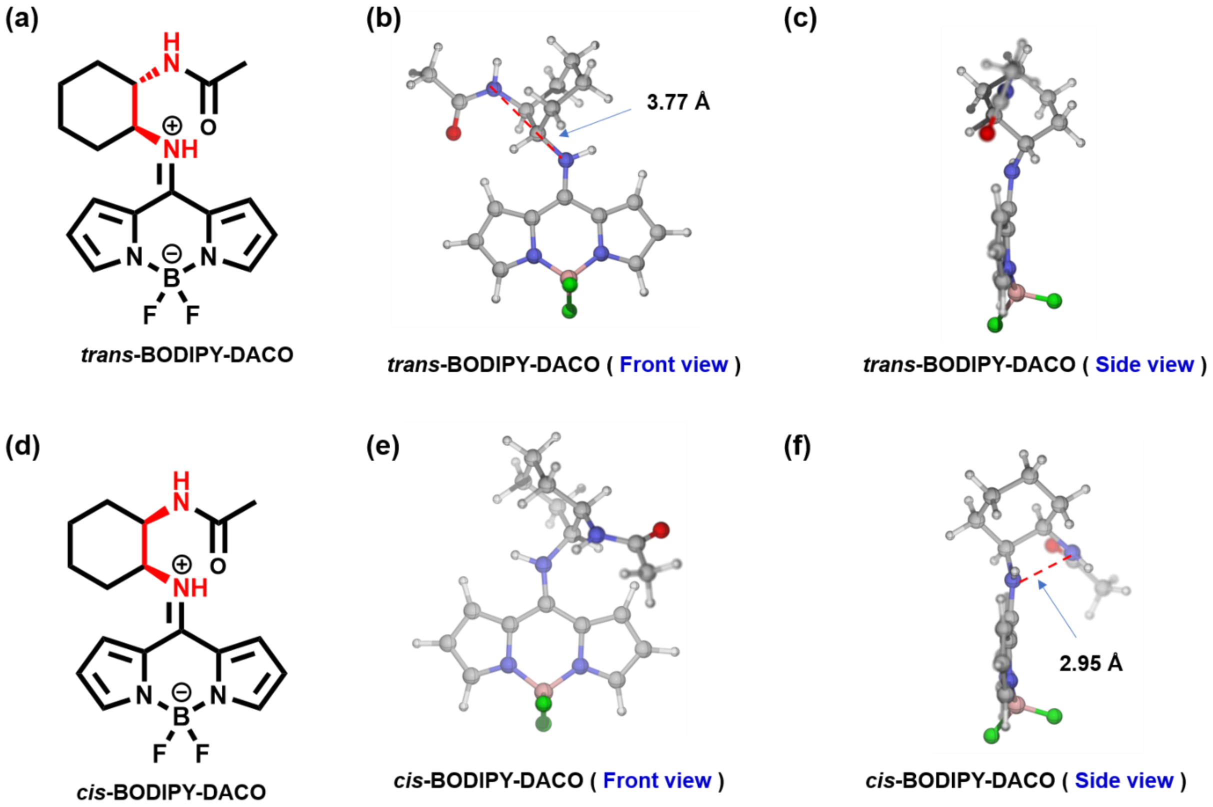

Figure S28. Optimized structures of *trans*-BODIPY-DACO and *cis*-BODIPY-DACO.

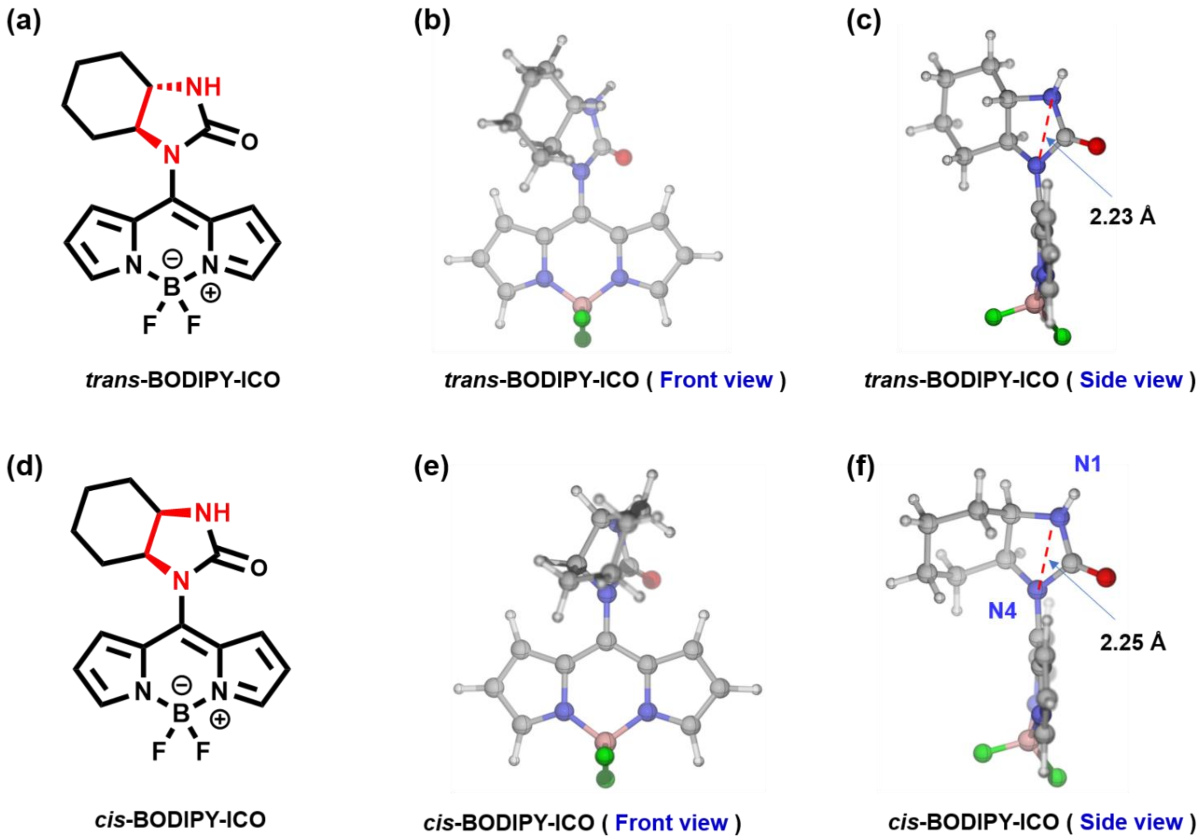

Figure S29. Optimized structures of *trans*-BODIPY-ICO and *cis*-BODIPY- DACO.

6. Density functional theory (DFT) calculations

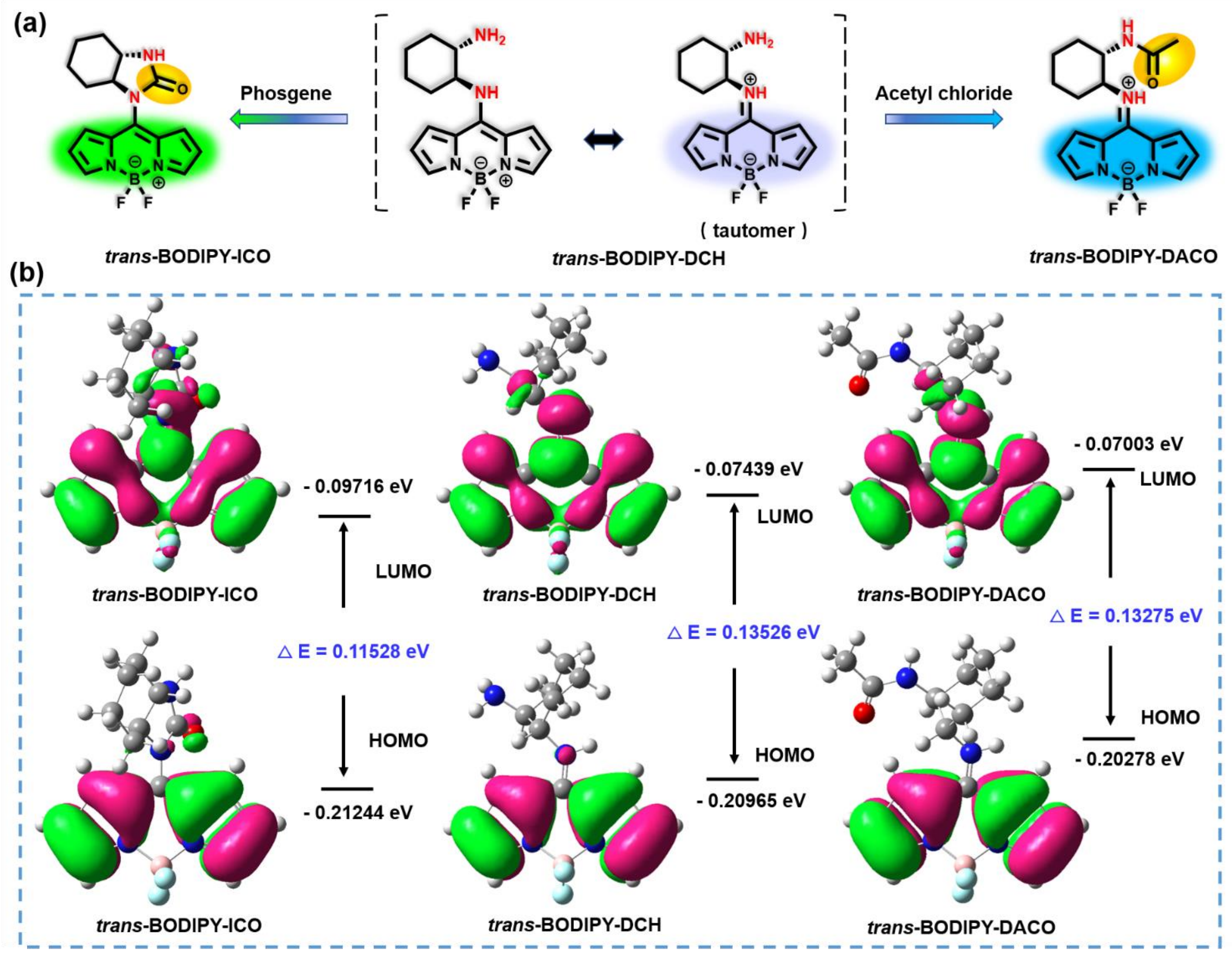

Figure S30. (a) Working mechanism of ***trans*-BODIPY-DCH** for phosgene and acetyl chloride. (b) Molecular amplitude plots the HOMO and LUMO of ***trans*-BODIPY-DCH**, *trans*-BODIPY-ICO and *trans*-BODIPY-DACO by using the Gaussian 09 program package.

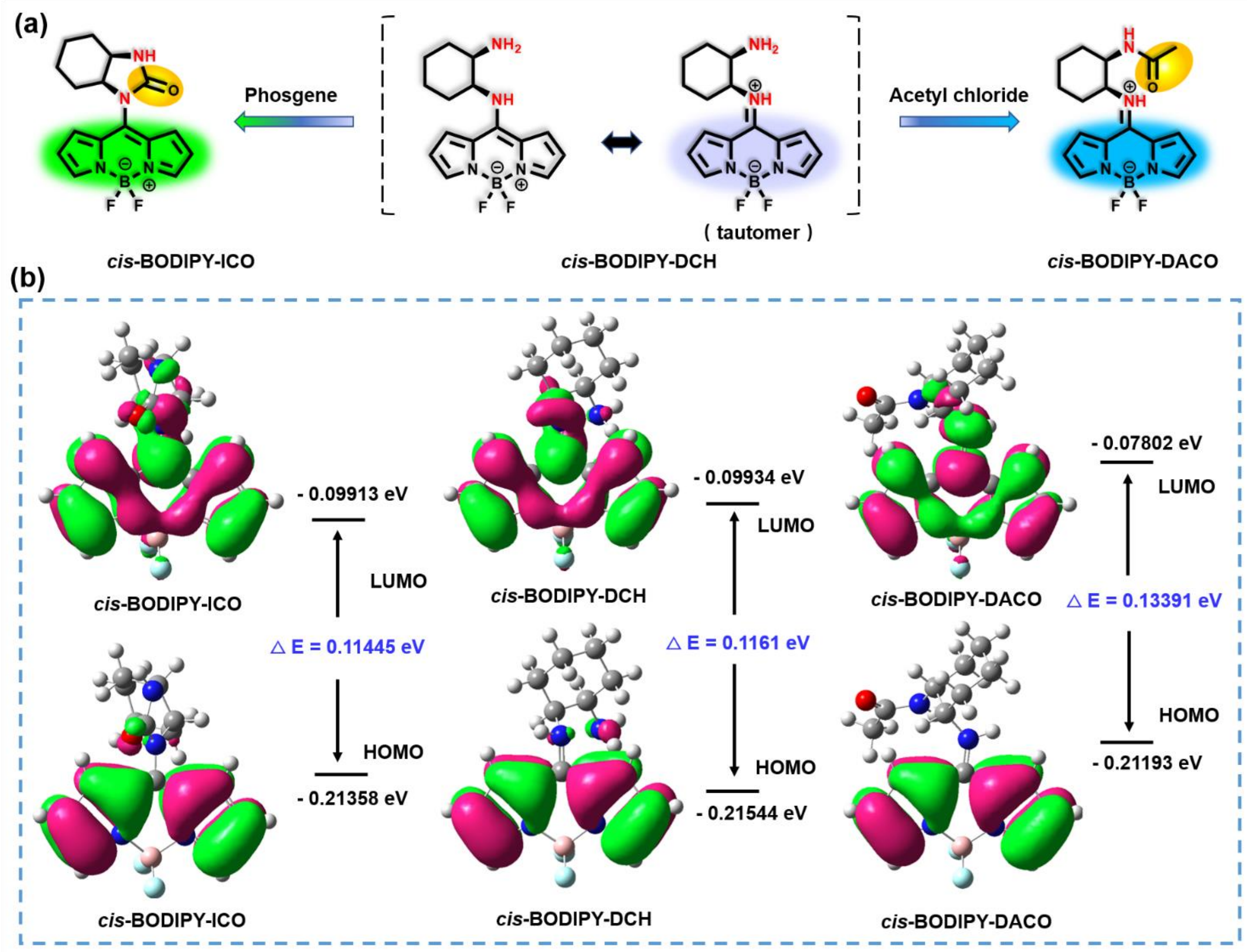

Figure S31. (a) Working mechanism of ***cis*-BODIPY-DCH** for phosgene and acetyl chloride. (b) Molecular amplitude plots the HOMO and LUMO of ***cis*-BODIPY-DCH**, *cis*-BODIPY-ICO and *cis*-BODIPY-DACO by using the Gaussian 09 program package.

7. Cytotoxicity of *trans*-BODIPY-DCH

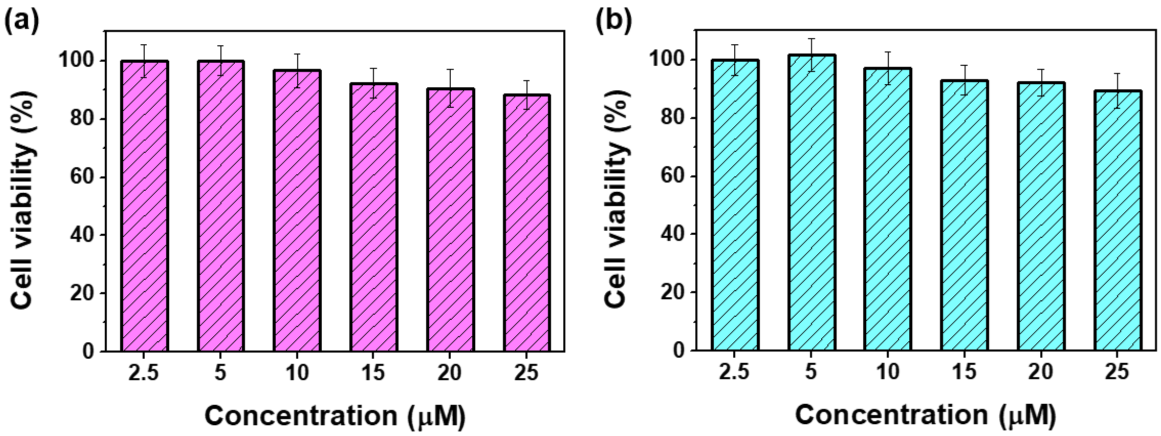

Figure S32. (a) L929 cells and (b) Hela cells viability after incubation with various concentrations of ***trans*-BODIPY-DCH** (0 – 25 μM) for 24 h. Error bars are ± SD, n = 5.

8. *trans*-BODIPY-DCH-loaded test strips for detection of analytes vapor

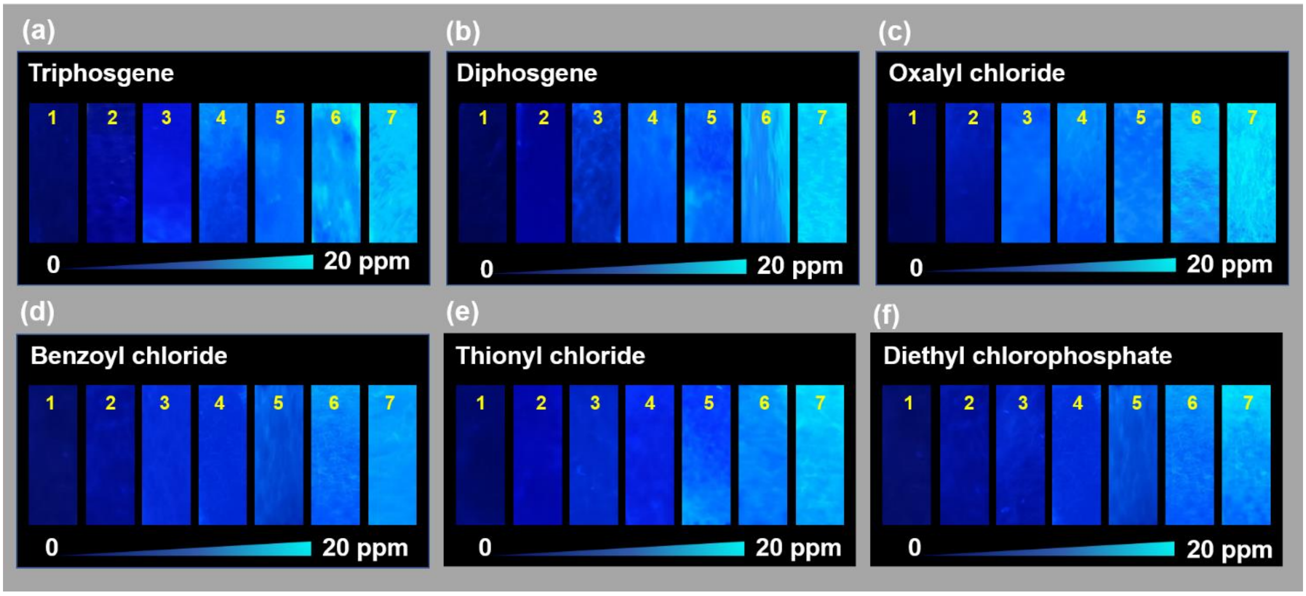

Figure S33. Fluorescence images of ***trans*-BODIPY-DCH**-loaded test strips after exposure to various analytes vapors at the concentration of 0 – 20.0 ppm. (1) 0, (2) 0.5, (3) 1.0, (4) 2.5, (5) 5.0, (6) 10.0, (7) 20.0 ppm.

9. Confocal fluorescence images of test strips for analytes vapor

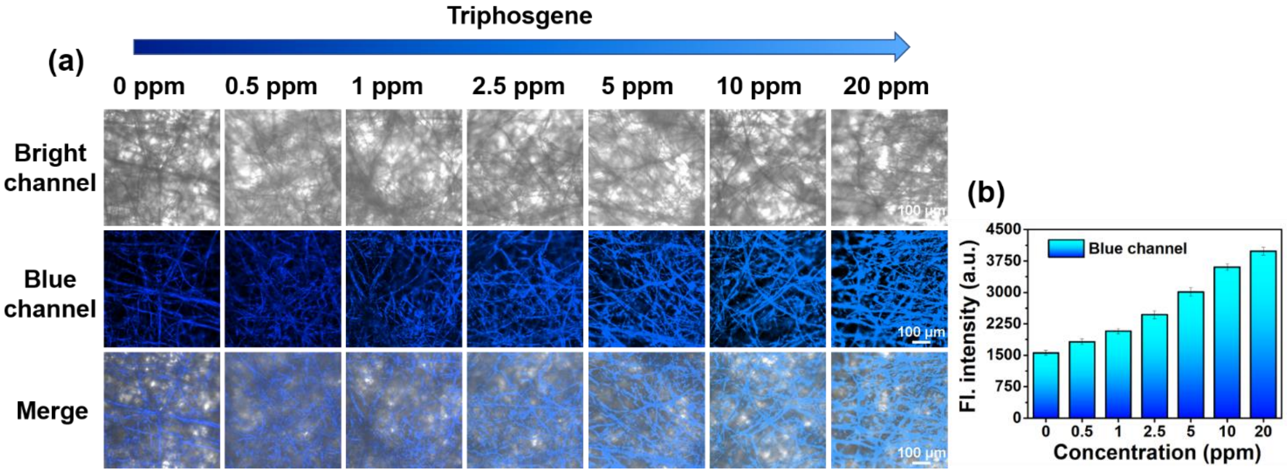

Figure S34. (a) Fluorescence images of ***trans*-BODIPY-DCH** test strips after exposure to triphosgene vapor. (b) Fluorescence intensity correlated to the level of triphosgene. λ_{ex} = 402 nm, λ_{em} = 425−475 nm. Error bars represent ± SD, n = 5. Scale bar: 100 μm.

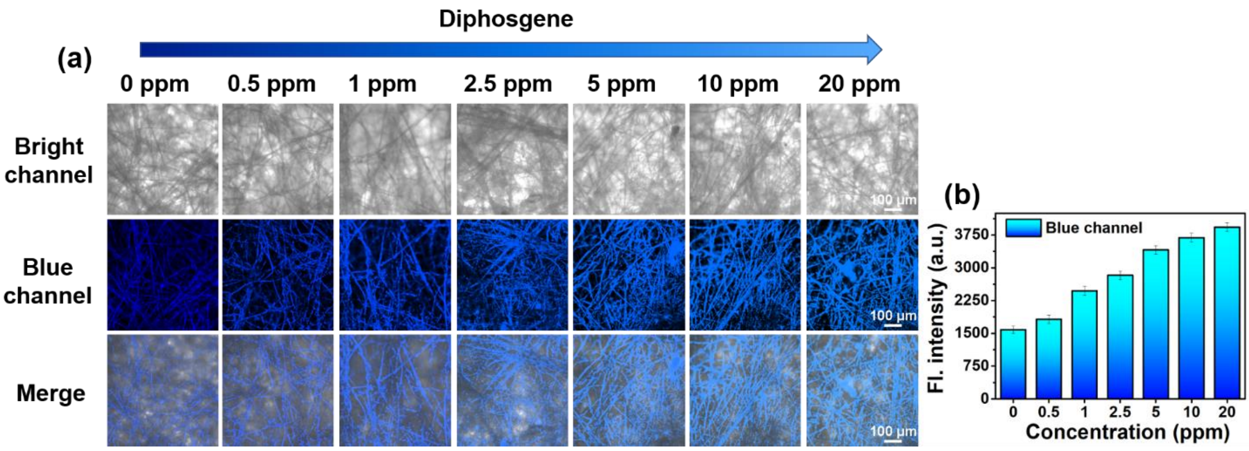

Figure S35. (a) Fluorescence images of ***trans*-BODIPY-DCH** test strips after exposure to diphosgene vapor. (b) Fluorescence intensity correlated to the level of diphosgene. λ_{ex} = 402 nm, λ_{em} = 425−475 nm. Error bars represent ± SD, n = 5. Scale bar: 100 μm.

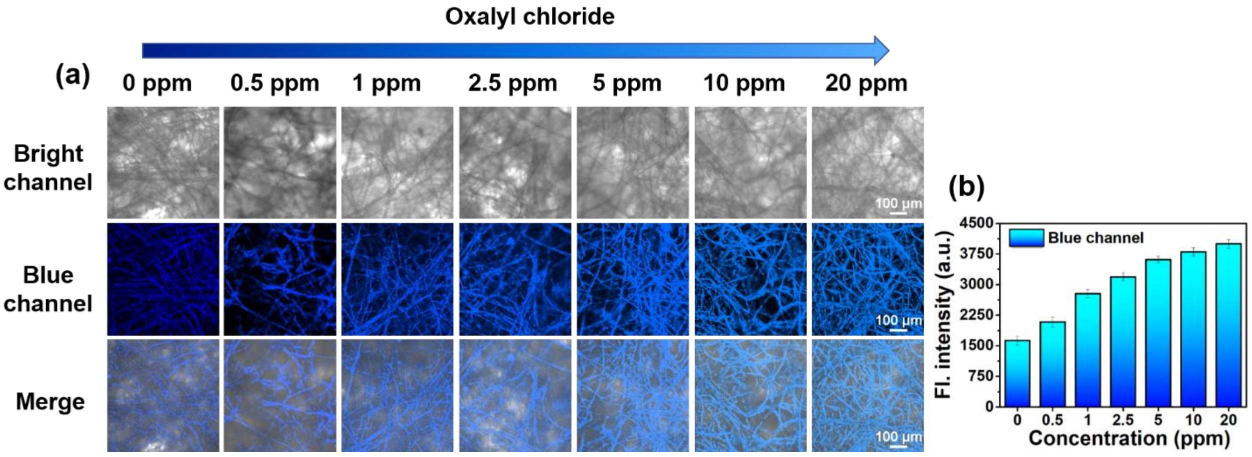

Figure S36. (a) Fluorescence images of ***trans*-BODIPY-DCH** test strips after exposure to oxalyl chloride vapor. (b) Fluorescence intensity correlated to the level of oxalyl chloride. λ_{ex} = 402 nm, λ_{em} = 425−475 nm. Error bars represent ± SD, n = 5. Scale bar: 100 μm.

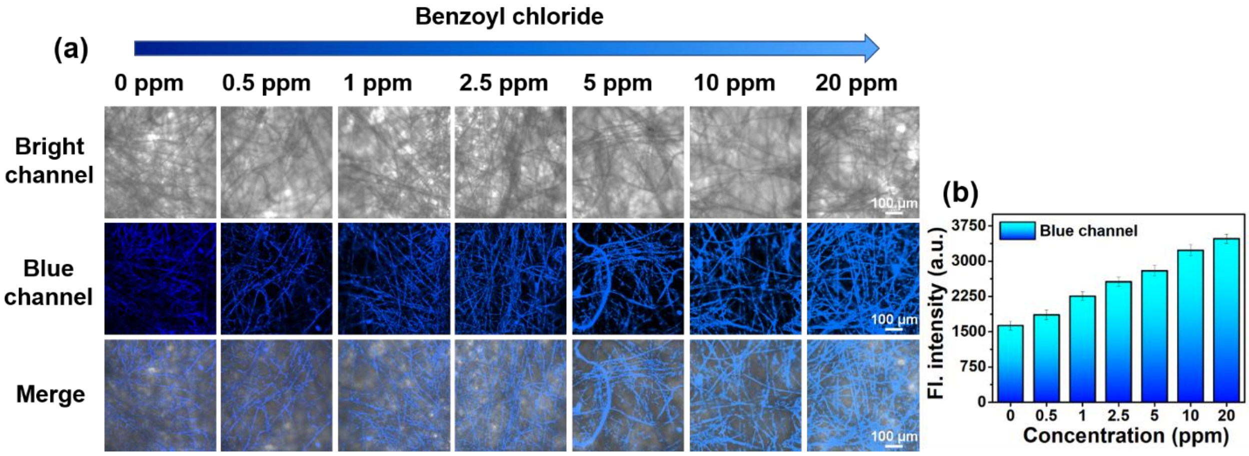

Figure S37. (a) Fluorescence images of ***trans*-BODIPY-DCH**-loaded test strips after exposure to benzoyl chloride vapor. (b) Fluorescence intensity correlated to level of benzoyl chloride. λ_{ex} = 402 nm, λ_{em} = 425−475 nm. Error bars represent ± SD, n = 5. Scale bar: 100 μm.

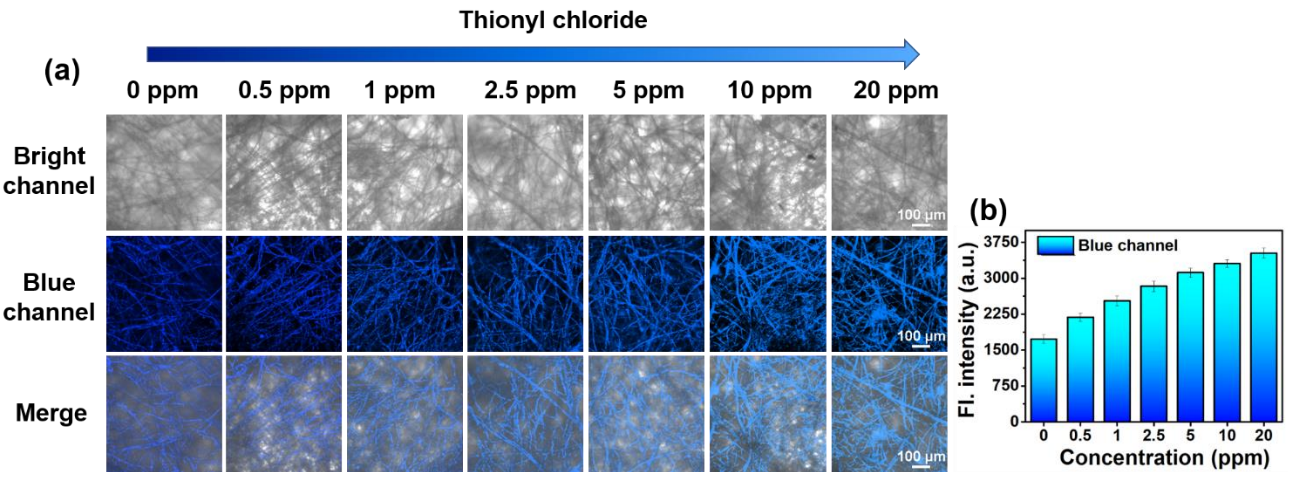

Figure S38. (a) Fluorescence images of ***trans*-BODIPY-DCH** test strips after exposure to thionyl chloride vapor. (b) Fluorescence intensity correlated to the level of thionyl chloride. λ_{ex} = 402 nm, λ_{em} = 425−475 nm. Error bars represent ± SD, n = 5. Scale bar: 100 μm.

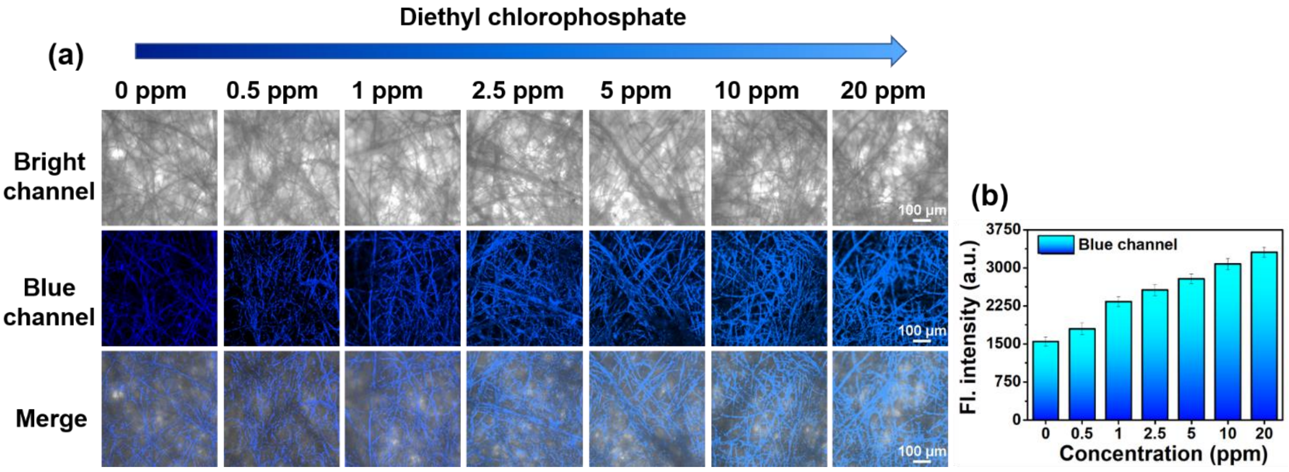

Figure S39. (a) Fluorescence images of ***trans*-BODIPY-DCH** test strips after exposure to diethyl chlorophosphate vapor. (b) Fluorescence intensity correlated to the level of diethyl chlorophosphate. λ_{ex} = 402 nm, λ_{em} = 425−475 nm. Error bars represent ± SD, n = 5. Scale bar: 100 μm.

10. Structure characterization

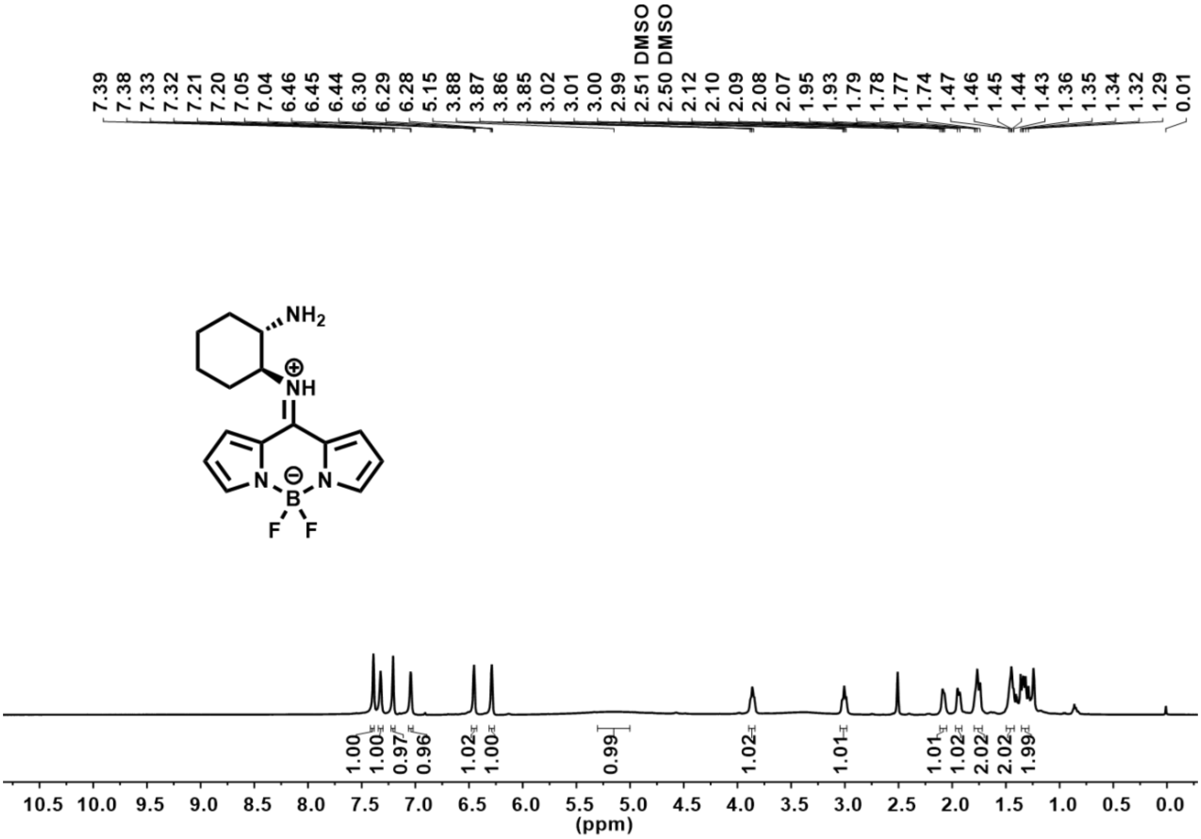

Figure S40. ^{1}H NMR spectrum of ***trans*-BODIPY-DCH** in DMSO-d_6 (500 MHz).

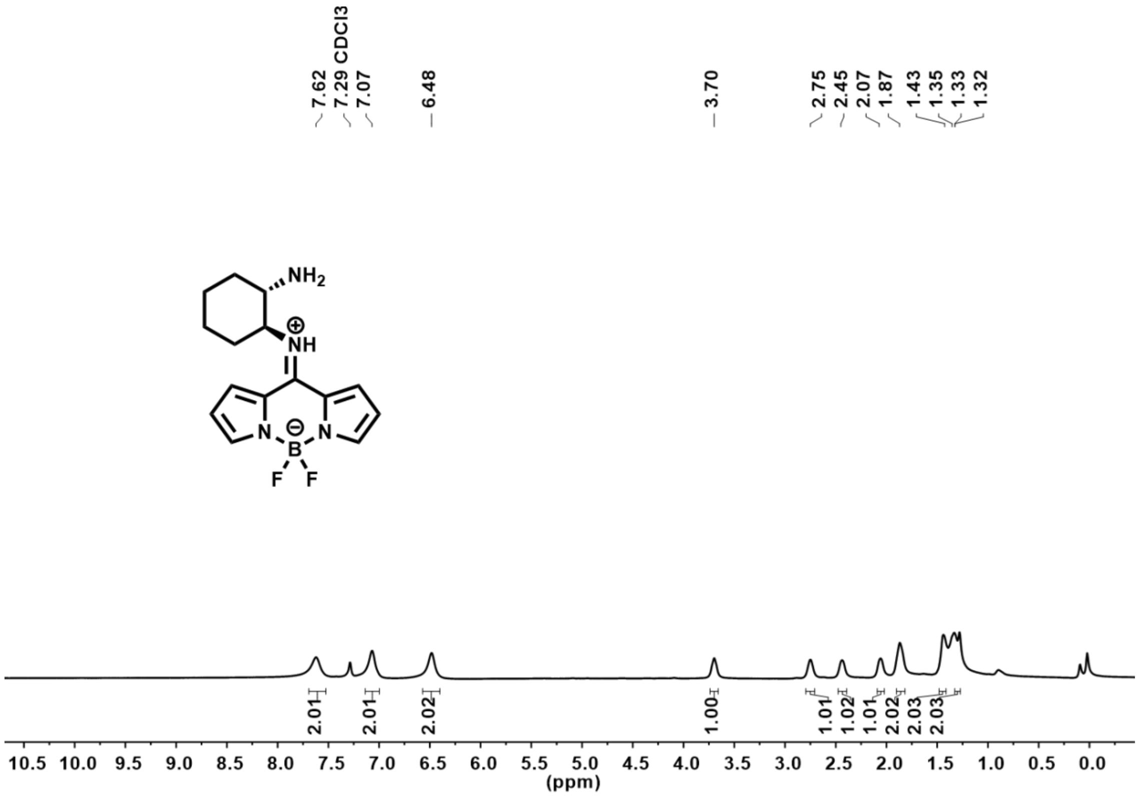

Figure S41. ^{1}H NMR spectrum of ***trans*-BODIPY-DCH** in $CDCl_3$ (500 MHz).

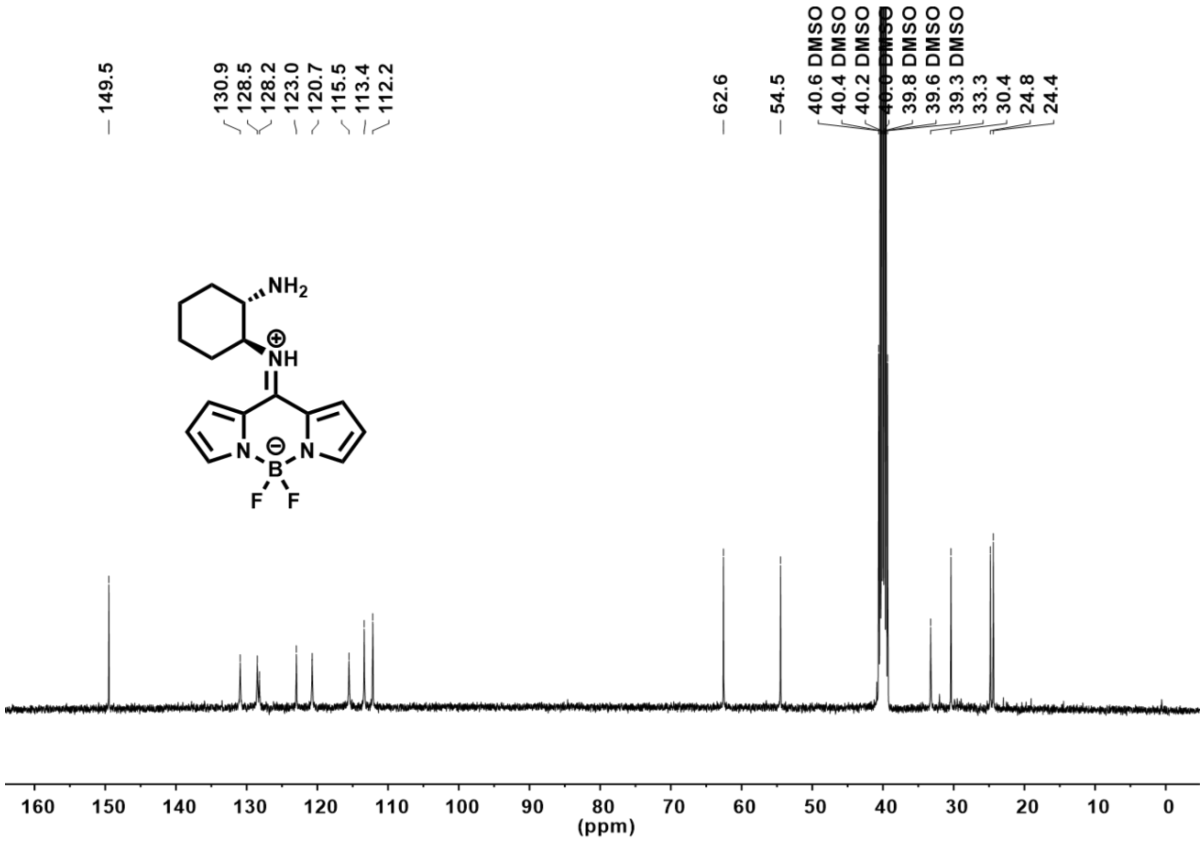

Figure S42. ^{13}C NMR spectrum of ***trans*-BODIPY-DCH** in DMSO-d_6 (125 MHz).

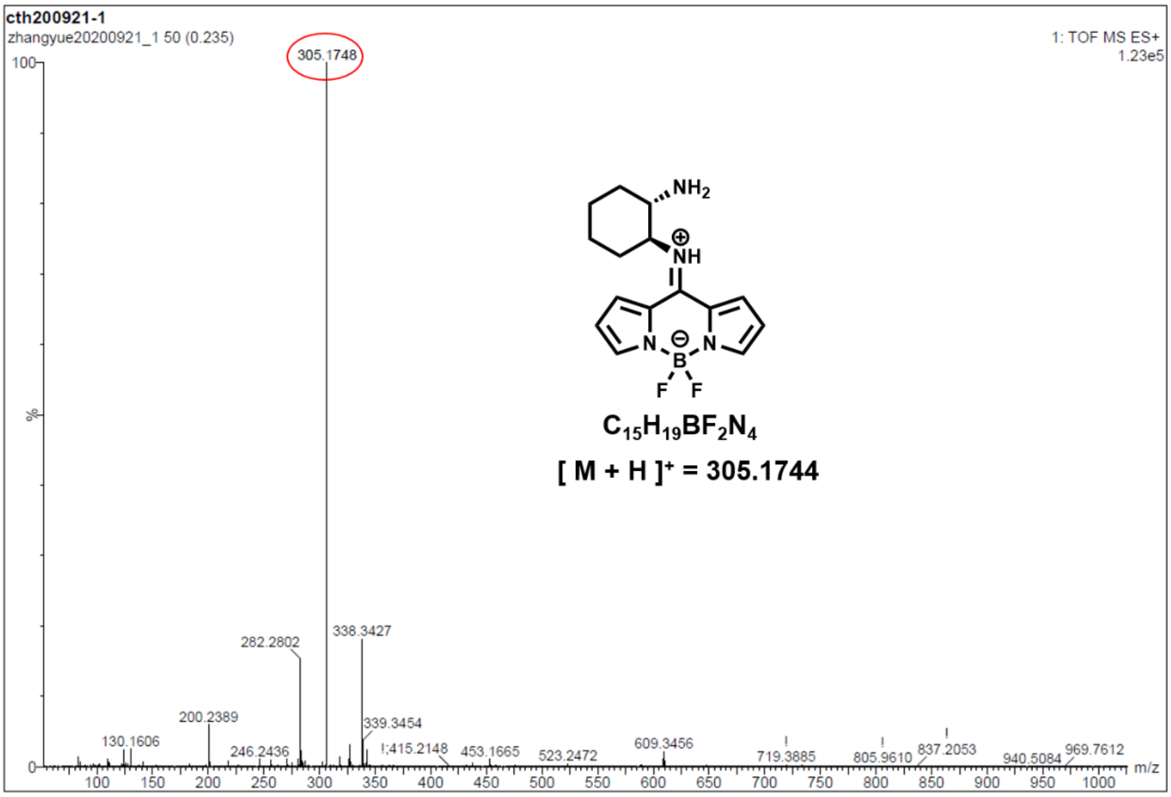

Figure S43. HR-MS spectra of ***trans*-BODIPY-DCH**.

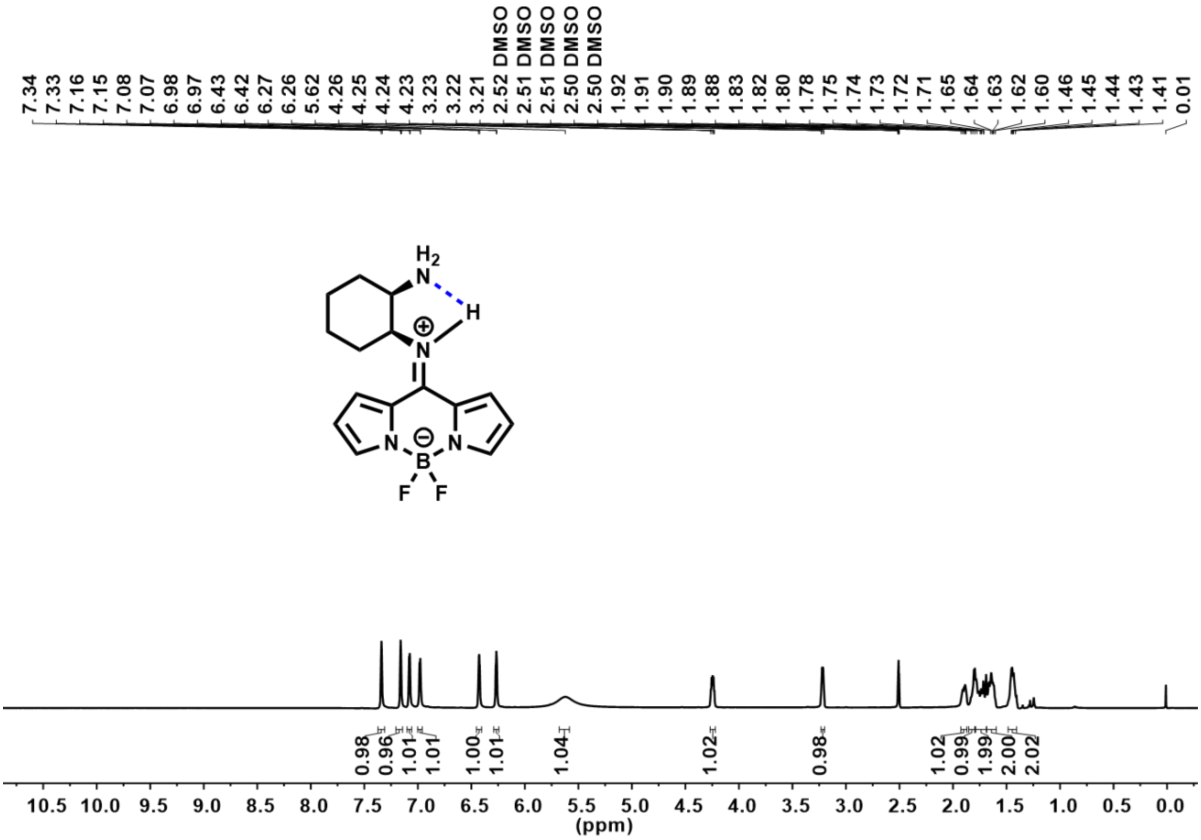

Figure S44. ^{1}H NMR spectrum of ***cis*-BODIPY-DCH** in DMSO-d_6 (500 MHz).

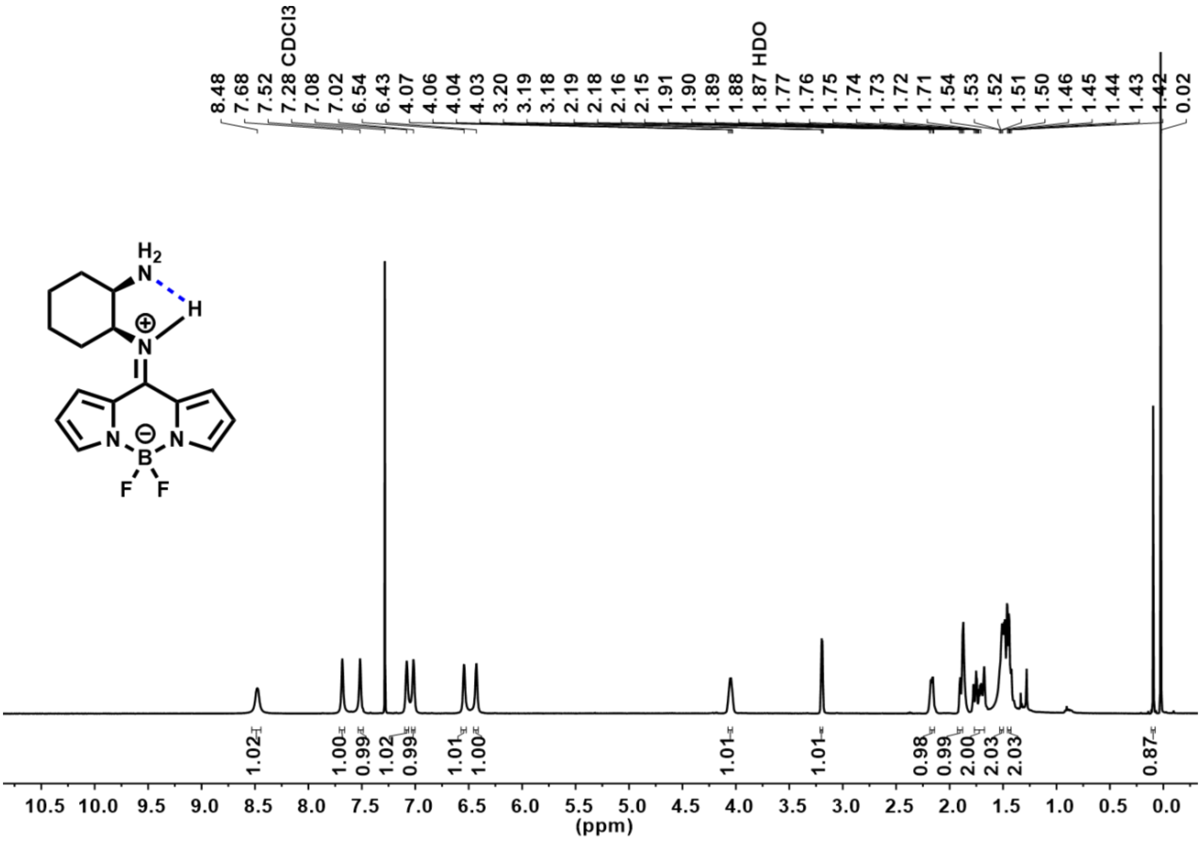

Figure S45. ^{1}H NMR spectrum of ***cis*-BODIPY-DCH** in $CDCl_3$ (500 MHz).

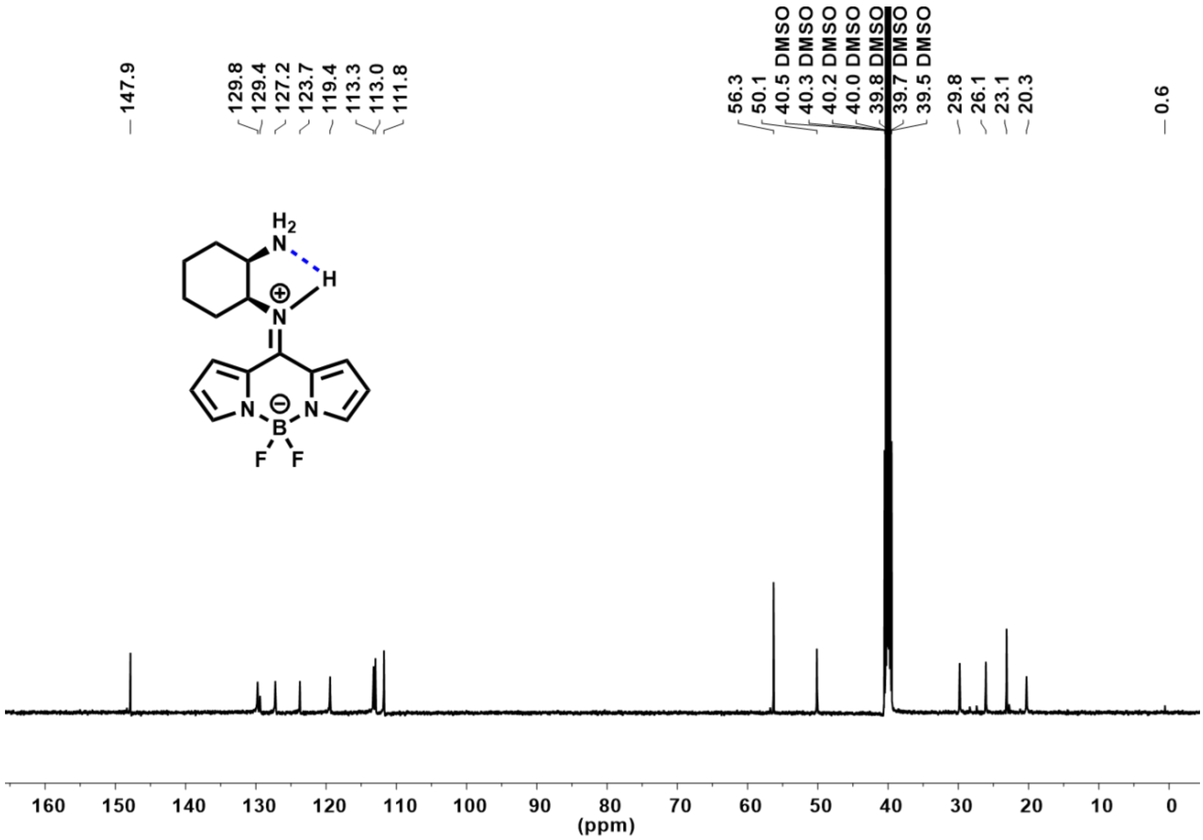

Figure S46. ^{13}C NMR spectrum of ***cis*-BODIPY-DCH** in DMSO-d_6 (125 MHz).

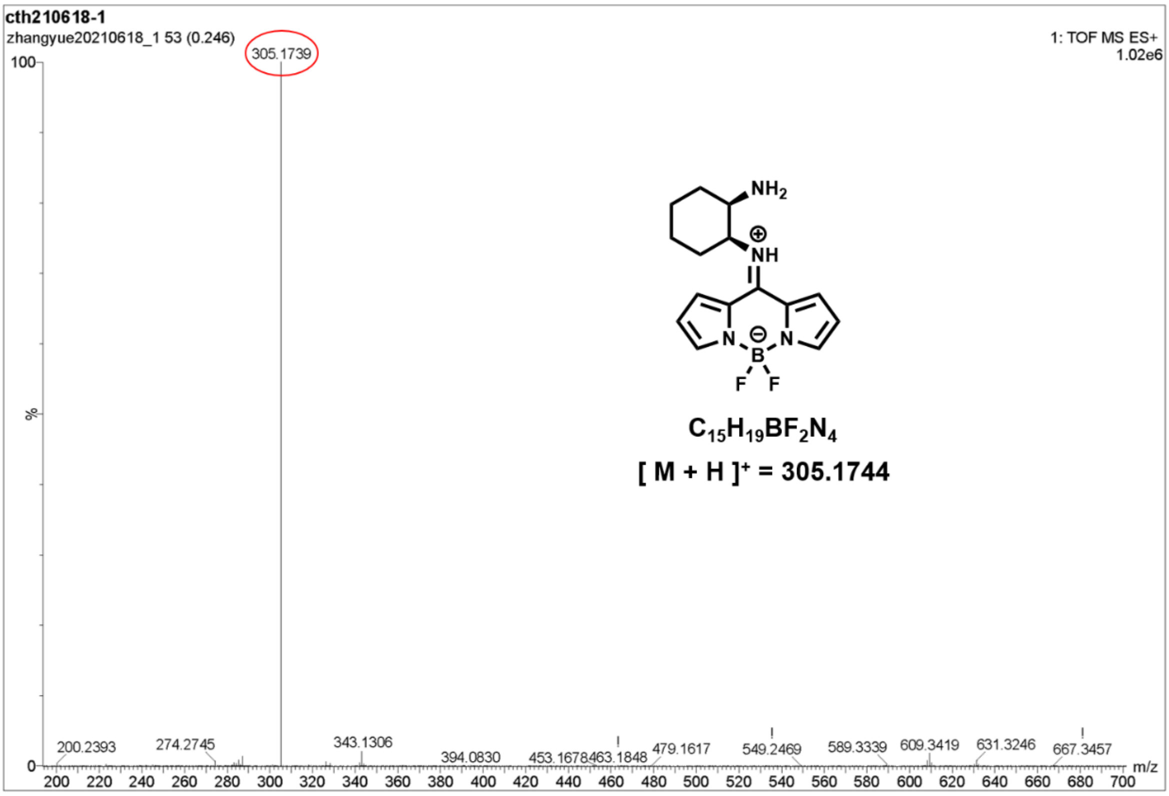

Figure S47. HR-MS spectra of ***cis*-BODIPY-DCH**.

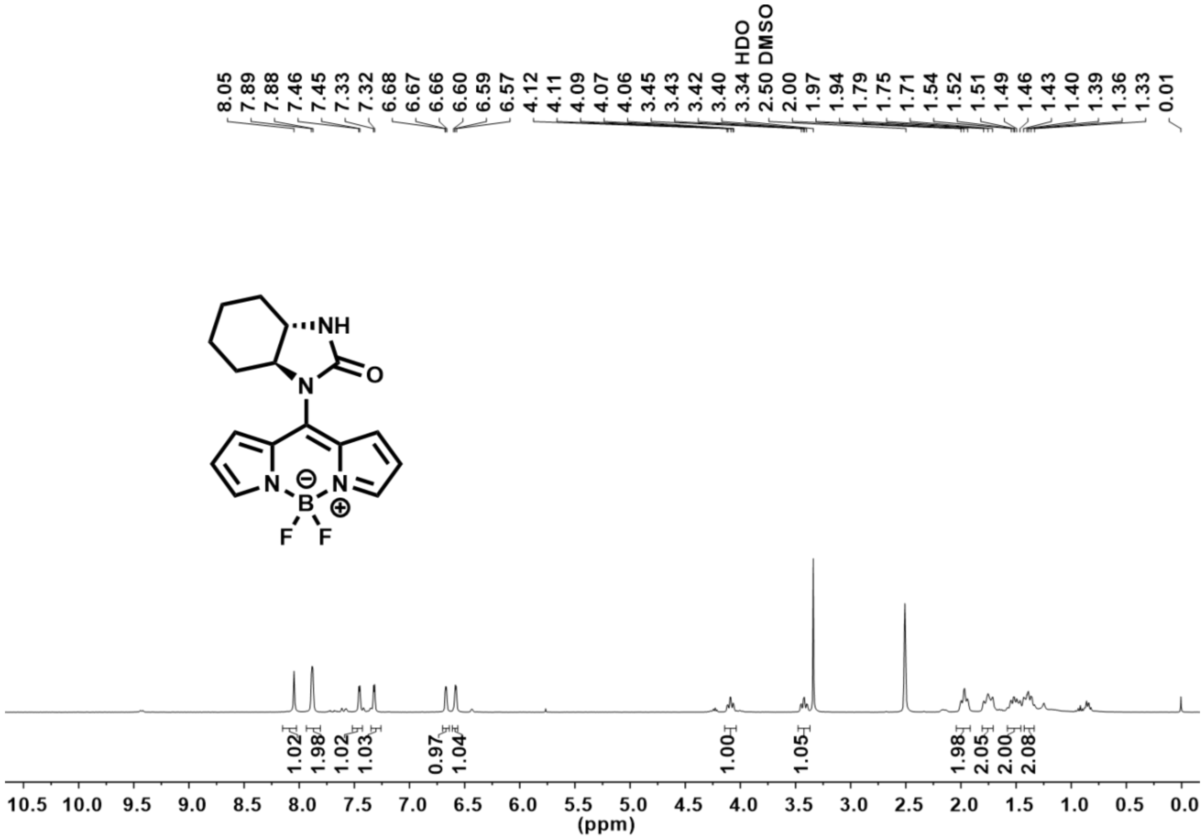

Figure S48. ^{1}H NMR spectrum of *trans*-BODIPY-ICO in DMSO-d_6 (500 MHz).

Table S1. The CIE1931 coordinates data of ***trans*-BODIPY-DCH** and ***cis*-BODIPY-DCH** for different concentrations of analytes.

CIE 1931 X/Y					
trans-BODIPY-DCH			*cis*-BODIPY-DCH		
Concentrations	Phosgene	Acetyl chloride	Concentrations	Phosgene	Acetyl chloride
0 nM	0.1274/ 0.1805	0.1251/ 0.1822	0 μM	0.1237/ 0.17	0.1253/ 0.1856
250 nM	0.1302/ 0.1969	0.1244/ 0.1796	2 μM	0.1294/ 0.2038	0.1221/ 0.1830
500 nM	0.1376/ 0.2317	0.1224/ 0.1759	4 μM	0.1335/ 0.2302	0.1255/ 0.1813
750 nM	0.1443/ 0.3001	0.1260/ 0.1734	6 μM	0.1395/ 0.2678	0.1254/ 0.1777
1 μM	0.1564/ 0.3261	0.1261/ 0.1711	8 μM	0.146/ 0.3071	0.1256/ 0.1732
1.5 μM	0.1697/ 0.3901	0.1261/ 0.1688	12 μM	0.1535/ 0.3557	0.1255/ 0.1713
2 μM	0.1848/ 0.4438	0.1265/ 0.1657	16 μM	0.1618/ 0.4026	0.1225/ 0.1662
2.5 μM	0.1974/ 0.5520	0.1261/ 0.1622	20 μM	0.1697/ 0.4513	0.1249/ 0.1629
3 μM	0.2020/ 0.5401	0.1261/ 0.1586	24 μM	0.1768/ 0.4932	0.1248/ 0.1602
3.5 μM	0.2031/ 0.5472	0.1261/ 0.1567	28 μM	0.1811/ 0.5184	0.1247/ 0.1581
4 μM	0.2036/ 0.5478	0.1237/ 1541	32 μM	0.1831/ 0.5317	0.1248/ 0.1572
4.5 μM	0.2038/ 0.5494	0.1261/ 0.1517	36 μM	0.1841/ 0.5361	0.1251/ 0.1566
5 μM	0.2046/ 0.5793	0.1260/ 0.1481	40 μM	0.1841/ 0.5371	0.1257/ 0.1556

Table S2. Fluorescence sensing properties of ***trans*-BODIPY-DCH** for different analytes.

Probe	Analytes	λ_{max} em [nm]	$\Phi_{FL}{}^{a,b}$	Response time [s]
Trans-BODIPY-DCH	--	478	0.21[a]	--
	Phosgene	524	0.46 [b]	3
	Triphosgene	478	0.68 [a]	15
	Benzoyl chloride	478	0.37 [a]	15
	Thionyl chloride	478	0.65 [a]	3
	Acetyl chloride	478	0.62 [a]	3
	Oxalyl chloride	478	0.64 [a]	6
	Diphosgene	478	0.66 [a]	3
	DCP	478	0.31 [a]	5

[a] Fluorescence quantum yields relative to quinine sulfate dihydrate in 0.5 M H_2SO_4 (Φ_f = 0.55). [b] Fluorescence quantum yields relative to fluorescein in 0.1 N NaOH (Φ_f = 0.90).

Table S3. Crystal data for ***trans*-BODIPY-DCH**, *trans*-BODIPY-ICO and *trans*-BODIPY-DACO.

Name	***trans*-BODIPY-DCH**	***trans*-BODIPY-ICO**	***trans*-BODIPY-DACO**
Identification code	9	210626-CTH	14
Empirical formula	$C_{15}H_{19}BF_2N_4$	$C_{16}H_{17}BF_2N_4O$	$C_{17}H_{21}BF_2N_4O$
Formula weight	304.15	330.14	346.19
Temperature/ K	293(2)	149.99(10)	293(2) K
Crystal system	tetragonal	triclinic	Orthorhombic
Space group	$P4_1$	P-1	P212121
a/Å	10.5675(8)	6.9141(2)	8.9883(16) Å
b/Å	10.5675(8)	9.2485(2)	11.1112(16) Å
c/Å	13.404(2)	12.7582(2)	17.485(3) Å
α/°	90	93.651(2)	90°.
β/°	90	101.649(2)	90°.
γ/°	90	97.353(2)	90°.
Volume/$Å^3$	1496.8(3)	789.08(3)	1746.2(5) Å3
Z	4	2	4
$\rho_{calc}g/cm^3$	1.350	1.390	1.317
μ/mm^{-1}	0.100	0.883	0.098
F(000)	640.0	344.0	728
Radiation	MoKα (λ = 0.71073)	CuKα (λ = 1.54184)	MoKα(λ=0.71076)
2Θ range for data collection/°	6.242 to 50.018	7.104 to 158.696	2.91 to 25.00
Index ranges	$-12 \le h \le 6$, $-12 \le k \le 12$, $-15 \le l \le 15$	$-8 \le h \le 8$, $-11 \le k \le 11$, $-16 \le l \le 15$	-10<=h<=10, -8<=k<=13, -17<=l<=20
Reflections collected	4917	8608	4429
Independent reflections	2626 [R_{int} = 0.0515, R_{sigma} = 0.0947]	3273 [Rint = 0.0384, Rsigma = 0.0430]	2833 [R(int) = 0.0411]
Data/restraints/parameters	2626/1/200	3273/0/235	2833 / 0 / 227
Goodness-of-fit on F^2	1.009	1.199	1.014
Final R indexes [I>=2σ (I)]	R_1 = 0.0450, wR_2 = 0.0832	R1 = 0.1281, wR2 = 0.2721	R1 = 0.0457, wR2 = 0.0866
Final R indexes [all data]	R_1 = 0.0936, wR_2 = 0.1069	R1 = 0.1335, wR2 = 0.2738	R1 = 0.0948, wR2 = 0.1166
Largest diff. peak/hole / e $Å^{-3}$	0.17/-0.22	0.40/-0.47	0.123/ -0.234

Table S4. Bond angles for ***trans*-BODIPY-DCH**.

Atom	Atom	Atom	Angle/°		Atom	Atom	Atom	Angle/°
C009	N003	C00C	130.9(4)		C00F	C00B	C009	134.1(5)
C00B	N004	B00M	127.1(4)		**N003**	**C00C**	**C008**	**108.9(4)**
C00D	N004	C00B	108.1(4)		N003	C00C	C00I	108.8(4)
C00D	N004	B00M	124.7(4)		C008	C00C	C00I	112.2(4)
C007	N006	B00M	125.9(4)		N004	C00D	C00J	110.8(5)
C00E	N006	C007	107.1(4)		N006	C00E	C00G	110.2(5)
C00E	N006	B00M	126.8(4)		C00J	C00F	C00B	108.2(5)
N006	C007	C009	120.9(4)		C00E	C00G	C00A	106.9(5)
N006	C007	C00A	108.9(4)		C00K	C00H	C008	113.8(4)
C00A	C007	C009	130.2(5)		C00C	C00I	C00L	112.7(4)
**N005**	**C008**	**C00C**	**109.5(4)**		C00D	C00J	C00F	106.1(5)
N005	C008	C00H	110.5(4)		C00H	C00K	C00L	110.3(4)
C00C	C008	C00H	110.6(4)		C00K	C00L	C00I	109.8(4)
N003	C009	C007	116.3(4)		F001	B00M	F002	107.6(5)
N003	C009	C00B	124.4(4)		F001	B00M	N004	110.7(5)
C00B	C009	C007	119.3(4)		F001	B00M	N006	112.2(5)
C007	C00A	C00G	106.9(4)		F002	B00M	N004	110.0(5)
N004	C00B	C009	119.0(4)		F002	B00M	N006	109.5(5)
N004	C00B	C00F	106.7(4)		N006	B00M	N004	106.8(4)

Table S5. Bond lengths for ***trans*-BODIPY-DCH**.

Atom - Atom	Length/Å		Atom	Atom	Length/Å
F001 - B00M	1.382(7)		C008 - C00C		1.523(6)
F002 - B00M	1.396(7)		C008 - C00H		1.531(6)
**N003 - C009**	**1.322(5)**		C009 - C00B		1.416(6)
**N003 - C00C**	**1.468(5)**		C00A - C00G		1.402(6)
N004 - C00B	1.402(6)		C00B - C00F		1.406(6)
N004 - C00D	1.346(6)		C00C - C00I		1.523(6)
N004 - B00M	1.537(7)		C00D - C00J		1.384(7)
N005 - C008	1.478(5)		C00E - C00G		1.368(7)
N006 - C007	1.374(5)		C00F - C00J		1.402(7)
N006 - C00E	1.363(6)		C00H - C00K		1.505(7)
N006 - B00M	1.526(7)		C00I - C00L		1.532(6)
C007 - C009	1.451(6)		C00K - C00L		1.506(7)
C007 - C00A	1.386(7)				

Table S6. Bond lengths for *trans*-BODIPY-DACO.

Atom - Atom	Length/Å		Atom - Atom	Length/Å
F001 - B1	1.399(5)		C00A - C00D	1.424(4)
O002 - C008	1.225(4)		C00B - C00F	1.513(5)
F003 - B1	1.372(5)		C00B - C00I	1.537(4)
N004 - C008	1.361(5)		C00C - C00K	1.381(5)
N004 - C00I	1.451(4)		C00D - C00E	1.428(4)
**N005 - C00D**	**1.330(4)**		C00E - C00G	1.392(4)
**N005 - C00B**	**1.472(4)**		C00F - C00N	1.529(4)
N006 - C00C	1.353(5)		C00G - C00H	1.400(5)
N006 - C00A	1.401(4)		C00H - C00L	1.374(5)
N006 - B1	1.538(5)		C00I - C00O	1.533(5)
N007 - C00L	1.366(4)		C00M - C00O	1.512(6)
N007 - C00E	1.380(4)		C00M - C00N	1.517(5)
N007 - B1	1.514(5)			
C008 - C00J	1.481(5)			
C009 - C00K	1.377(5)			
C009 - C00A	1.413(5)			

Table S7. Bond lengths for *trans*-BODIPY-ICO.

Atom - Atom	Length/Å		Atom - Atom	Length/Å
F001 - B00L	1.386(8)		C009 - C00A	1.381(7)
O002 - C00E	1.217(7)		C009 - C00D	1.415(7)
F003 - B00L	1.376(8)		C00B - C00C	1.360(8)
N004 - C008	1.394(6)		C00B - C00G	1.382(8)
N004 - C00G	1.350(7)		C00D - C00F	1.375(8)
N004 - B00L	1.545(7)		C00F - C00H	1.409(9)
N005 - C009	1.398(6)		C00I - C00K	1.460(8)
N005 - C00H	1.341(7)		C00I - C00O	1.521(12)
N005 - B00L	1.551(8)		C00I - C00P	1.420(12)
N006 - C00E	1.399(7)		C00J - C00M	1.509(10)
**N006 - C00A**	**1.411(6)**		C00J - C00O	1.418(12)
**N006 - C00K**	**1.422(7)**		C00J - C00P	1.671(13)
N007 - C00E	1.353(7)		C00K - C3	1.398(17)
N007 - C00I	1.414(8)		C00K - C7	1.458(16)
C008 - C00A	1.392(7)		C00M - C3	1.434(17)
C008 - C00C	1.419(7)		C00M - C7	1.467(14

Table S8. Summary of fluorescent chemosensors for phosgene and DCP.

	Phosgene			DCP			
Structures	Response time	LOD	Emission wavelength	Response time	LOD	Emission wavelength	References
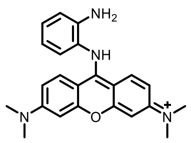	Seconds	20 nM	593 nm	> 2 min	147 nM	538 nm	Angew. Chem. In. Ed. 2016, 55, 4729-4733
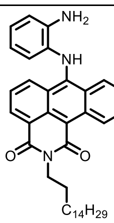	< 2 min	72 nM	600 nm	6 s	88 nM	588 nm	Anal. Chem. 2019, 91, 12070-12076
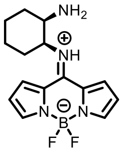	4 s	23.49 nM	522 nm	5 s	6.92 nM	474 nm	This work
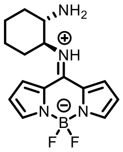	3 s	0.52 nM	524 nm	5 s	0.77 nM	478 nm	This work

11. References

[S1] Y. H. Lee, S. Jana, H. Lee, S. U. Lee, M. H. Lee, *Chem. Commun*. **2018**, *54*, 12069−12072.

[S2] J. L. Tan, M. X. Zhang, F. Zhang, T. T. Yang, Y. Liu, Z. B. Li, H. Zuo, *Spectrochim. Acta A* **2015**, *140*, 489−494.

[S3] A. M. Brouwer, *Pure Appl. Chem.* **2011**, *83*, 2213−2228.

[S4] Y. Zhang, X. Shao, Y. Shao, F. Pan, R. Kang, F. Peng, Z. Huang, W. Zhang, W. Zhao, *Chem. Commun*, **2015**, *51*, 4245−4248.

[S5] Z. Mao, M. Ye, W. Hu, X. Ye, Y. Wang, H. Zhang, C. Li, Z. Liu, *Chem. Sci.* **2018**, *9*, 6035−6040.
